# USP9X integrates TGF-β and hypoxia signalings to promote ovarian cancer chemoresistance via HIF-2α-maintained stemness

**DOI:** 10.1038/s41419-025-07646-5

**Published:** 2025-04-18

**Authors:** Zhenlei Zhang, Xiujie Yu, Liqi Wen, Jia’nan Wang, Zhufeng Li, Yu Zhang, Jiayu Cheng, Ronglin Kan, Wanting Zhang, Yan Shen, Shukai Yuan, Li Zhao

**Affiliations:** 1 Department of Thyroid and Neck Oncology, Key Laboratory of Cancer Prevention and Therapy, Tianjin’s Clinical Research Center for Cancer, National Clinical Research Center for Cancer, Tianjin Medical University Cancer Institute and Hospital, Key Laboratory of Immune Microenvironment and Disease (Ministry of Education), Department of Biochemistry and Molecular Biology, Tianjin Medical University, Tianjin, China; 2 Tianjin Key Laboratory of Human Development and Reproductive Regulation, Department of Pathology, Tianjin Central Hospital of Gynaecology Obsterics, Tianjin, China; 3 Department of General Surgery, Tianjin Medical University General Hospital, Tianjin, China

**Keywords:** Cancer stem cells, Ubiquitylation, Ovarian cancer, Cancer therapeutic resistance

## Abstract

Widespread intraperitoneal metastases and chemoresistance render ovarian cancer the leading cause of gynecological malignancy-related deaths, wherein TGF-β signaling plays the pivotal role by promoting cancer stem cells (CSCs) activity. The activation mechanism and key protumorigeneic events downstream of TGF-β signaling remain incompletely understood. Here, we identify hypoxic tumor microenvironment as an initiator of TGF-β signaling to promote HIF-2α positive CSC-mediated chemoresistance in high-grade serous ovarian cancer (HGSOC). Mechanistically, deubiquitinase USP9X, as a TGF-β downstream effector, stabilizes HIF-2ɑ in a hydroxylation- and ubiquitylation-dependent manner, thus promoting stemness reprogramming. Hypoxia and TGF-β signals converge on USP9X-HIF-2ɑ axis via multi-level regulations, which in turn facilitates Smad/HIF responses. Clinically, USP9X expression correlates with TGF-β signatures, CSCs characteristics, EMT behaviors, and chemotherapy responsiveness, along with HIF-2ɑ. Antagonizing USP9X efficiently represses tumor formation, metastasis, CSCs occurrence, while increasing chemosensitivity in orthotopic tumors, patient-derived xenograft (PDX), organoid, and chemoresistant cell models, in part via restricting TGF-β and hypoxia activities. This study deciphers the critical role of hypoxic niche in stimulating TGF-β signaling, and a downstream USP9X-HIF-2ɑ proteostatic regulatory axis in priming the HGSOC stemness, thereby provides novel targeting venues to counteract TGF-β signaling in CSCs and meliorate clinical chemoresistance.

## Introduction

Epithelial Ovarian cancer (EOC) is the leading cause of gynecologic malignancy-related deaths. As the most prevalent type, high-grade serous ovarian cancer (HGSOC) presents the advanced stage with malignant ascites and widespread metastases, and accounts for 70–80% of EOC mortalities [1, 2]. Although HGSOC is among the most chemosensitive malignancies at the time of initial treatment, most patients ultimately experience tumor relapse and recurrence, and succumb to chemoresistant disease. Accumulating evidence suggests that recurrence and resistance to chemotherapy of ovarian cancer are mainly attributed to the function of cancer stem cells (CSCs) [3, 4]. For example, the CD133, ALDH1A1, FZD7 enriched ovarian cancer stem cells showed platinum and adriamycin resistance and enhanced EMT properties [5–7]. High abundance of CD133 and ALDH1 CSCs markers in ovarian cancer patient tumor samples predict a worse outcome [8]. Thus, elucidating key pathways and molecular mechanisms in modulating CSCs function holds the promise to identify reliable biomarkers for diagnosis stratification as well as for therapeutic targeting.

CSCs are enriched with more mesenchymal gene signatures that coincide with stem cell-like features. Transforming growth factor-β (TGF-β) signaling, as the key trigger for metastasis, induces EMT and dedifferentiation to promote stemness in ovarian cancers [9–12]. Importantly, chemotherapeutic agents, such as doxorubicin, paclitaxel, and cisplatin activate TGF-β signaling to induce EMT and CSCs enrichment in human ovarian cancer and cervical cancer [13]. The latest HGSOC single-cell RNA sequencing again shows that chemotherapy induces alterations in tumor characteristics and significant upregulation of the TGFβ pathway [14]. Indeed, by analyzing EOC patients treated with platinum-based chemotherapy, single nucleotide polymorphisms (SNPs) associated with survival and platinum-free interval (PFI) mostly enrich to TGF-β pathway [15]. Combined therapy with a novel TGF-β trap RER and cisplatin neutralizes cisplatin-stimulated TGF-β thus lead to more efficacious inhibition of ovarian cancer growth [13]. Likewise, TGF-β signaling also induces CSCs-driven platinum, paclitaxel, 5-fluorouracil, and docetaxel resistance in breast cancer, gastric cancer, and prostate cancer [16–19]. Theoretically, targeting TGF-β signaling would become a promising strategy to conquer the clinical chemoresistance cancers. Whereas, systemic inhibition of TGF-β leads to unexpected side effects and safety concerns due to highly pleiotropic functions, thus hindering the translational application of TGF-β antagonists into clinic [20, 21]. In this case, to seek targetable upstream initiators and downstream effectors of TGF-β signaling is of great importance for managing metastatic and chemoresistant EOC.

USP9X, a structurally and functionally conserved deubiquitinase, functions as an essential modulator of TGF-β signaling by controlling SMAD4 monoubiquitination [22]. The vital interaction of USP9X and SMAD4 mediates free fatty acids (FFA)-induced aberrant TGF-β activation and obesity-promoted breast cancer metastasis [23]. USP9X-mediated KDM4C deubiquitylation promotes lung cancer radioresistance by epigenetically inducing TGF-β2 transcription [24]. Additionally, TGF-β signaling promotes Usp9X phosphorylation, which enhances its stabilization of ankyrin-G protein and leads to spine enlargement [25]. Thus, USP9X plays critical roles downstream of TGF-β signaling during pathological malignancy and physiological organ formation. As ovarian malignancy is concerned, USP9X was found to be mutated in 27% of patients and could be the key driver and therapeutic vulnerability of low-grade serous ovarian carcinoma cancer (LGSOC) [26, 27]. And also, USP9X gene variants significantly relate to premature ovarian failure (POF), a heterogeneous ovary developmental defect [28]. Whether USP9X participates in TGF-β signaling controlled pathological events and therapeutic response in ovarian cancer progression is thus worthy exploring.

Herein, we found USP9X, as an important downstream effector, mediates TGF-β-induced stemness and chemoresistance of HGSOC, thus highly correlates with primary CSCs contents. Antagonizing USP9X efficiently represses tumor formation, metastasis, while increases chemo-sensitivity through patient derived xenograft (PDX), organoid models and chemoresistant cell models. Systematic screening identifies HIF-2α as the direct DUB substrate of USP9X that maintains CSCs function and promotes HGSOC development. More importantly, we found that the hypoxia tumor microenvironment activates the TGF-β-USP9X-HIF-2α CSCs regulatory axis. Our current study demonstrates the key oncogenic role of USP9X that coordinates TGF-β and hypoxia signalings to mediate HGSOC CSCs function and chemoresistance, thus highlights the promising diagnostic and therapeutic prospects.

## Materials and methods

### Ethics

Deidentified OC surgical specimens were collected from Tianjin Central Hospital of Gynecology Obstetrics in accordance with the Institutional Review Board-approved protocol (2023KY005). All mice experiment procedures and protocols were evaluated and authorized by the Regulations of Tianjin Laboratory Animal Management and strictly followed the guidelines under the Institutional Animal Care and Use Committee of Tianjin Medical University (Approval number: SYXK-(Jin) 2020-0010).

### Mice

The mice used in the study were all female. Orthotopic tumor model mice used in this study were maintained as specific-pathogen-free (SPF) mice on a C57BL/6 genetic background. And, patient-derived xenografts (PDX) model was established on BALB/c nude mice.

### Cell lines

HEK293T cells (ACS-4500) and ovarian cancer cell lines SKOV3 were purchased from the American Type Culture Collection (ATCC). High-grade serous ovarian cancer (HGSOC) cell line CAOV3 and mouse ovarian cancer cell line ID8 were obtained from Nankai University with STR profiles. All cell lines are free of mycoplasma contamination. HEK293T, CAOV3 and ID8 cell lines were maintained in DMEM medium supplemented with 10% fetal bovine serum (FBS). SKOV3 were maintained in RPMI 1640 medium supplemented with 10% FBS. Ovarian cancer primary cells were isolated from HGSOC patients and cultured in DMEM: F12 (1:1) supplemented with 10% FBS, 100 μg/mL streptomycin, 10 ng/mL epithelial growth factor (EGF), 5 μg/mL insulin, and 0.5 mg/mL hydrocortisone. All the cells were cultured in a humidified incubator equilibrated with 5% CO_2_ at 37 °C. For hypoxia experiments, cells were cultured in a humidified incubator equilibrated with 94% N_2_, 5% CO_2_ and 1% O_2_ at 37 °C.

### Plasmids

The Flag-tagged full length of USP9X wild-type and DUB-dead mutant USP9X^C1566S^ plasmids were kindly gifted from Dr. Lei Shi. The plasmid constructs expressing Flag-tagged mutants of USP9X (N, U, C, and D) were cloned into vector pCDH-CMV-puro. The plasmid constructs expressing HA-tagged full-length and truncated mutants HA-HIF-2α (H1, H2, and H3) were cloned into vector pLV-EF1α-BSD. The full length Smad2 or Smad3 CDS was cloned into pCDNA3.1 vector. The full length (2192 bps) for USP9X promoter or the full length (2072 bps) for TGF-β1 promoter was cloned into pGL4.23 vector. For prokaryotic expression, USP9X-N, C, or D was cloned into pGEX-6P-2 vector, and HIF-2α was cloned into pET-28a vector. Recombinant lentiviruses expressing different shRNAs were obtained by cloning designed shRNA into pLKO.1-puro. shRNA sequences are listed in Supplementary Table S1.

### Antibodies and reagents

Antibodies used for immunoblot (IB), immunoprecipitation (IP), immunohistochemistry (IHC) and immunofluorescence (IF) were as follows. Anti-Flag (#3165, 1:1000), Anti-α-Tubulin (T5168, 1:15000) was purchased from Sigma. Anti-HA (#3724, 1:1500), Anti-Ubiquitin (#3936, 1:500), Anti-GST (#2622, 1:1000), was purchased from Cell Signaling Technology. Anti-USP9X (#55054-1-AP, Proteintech, 1:1000), Anti-Ki-67 (#ab15580, Abcam, 1:200), Anti-PAX8 (#ab53490, Abcam, 1:10), Anti-p-Smad3 (#ab52903, Abcam, 1:1000), Anti-HIF-1α (#NB100-134, NOVUS, 1:1000), Anti-HIF-2α (#NB100-122, NOVUS, 1:1000), Anti-HIF-2α (#GTX30123, Genetex, 1:200), Anti-HIF-2α (#ab243861, Abcam, 1:200), Anti-E-Cadherin (#YM0207, ImmunoWay, 1:1000), Anti-N-Cadherin (#YM0465, ImmunoWay, 1:1000), Anti-Slug (#GTX128796, Genetex, 1:1000), Anti-Twist (#ab175430, Abcam, 1:1000), Anti-Snail (#GTX638370, Genetex, 1:200) were employed according to the instructions. Reagents used in this study included MG132 (#S2619, Selleck), Puromycin (#S7417, Selleck), CHX (#S7418, Selleck), Blasticidin (#S7419, Solario), WP1130 (#S1267, MCE), Cisplatin (#S1166, Selleck), Carboplatin (#S1215, Selleck), Paclitaxel (#S1150, Selleck), Beetle luciferin potassium salt (#E1605, Promega), Accutase (#40506ES60, Yeason).

### Mass spectrometry analysis

Flag-tagged USP9X or HIF-2α plasmid was transfected into HEK293T cells for 48 h, lysed with lysis buffer (50 mM Tris-HCl, pH = 7.4, 150 mM NaCl, 5 mM EDTA, 0.5% NP-40) freshly supplemented with protease inhibitor cocktail. Whole-cell lysates were then incubated with Flag-M2 beads to precipitate USP9X or HIF-2α and associated partners. Beads were washed five times using the lysis buffer, then eluted with 100 μg/mL FLAG peptides (Sigma-Aldrich) in cold PBS. The eluted proteins were resolved in 5 × SDS loading buffer, boiled at 99 °C for 10 min. The eluted proteins were separated by SDS-PAGE Gel, stained using Coomassie brilliant blue, and then in-gel tryptic digestion, subjected to Thermo Scientific TM Q Exactive^TM^ Plus. *n* = 2 biological replicates. Secondary mass spectrometry data were retrieved using Proteome Discoverer 1.3.

### Immunoprecipitation

For immunoprecipitation, 60 μl of 50% protein A/G Agarose beads (Invitrogen) were incubated with control or specific antibodies (2–4 μg) for 8 h at 4 °C with constant rotating, then centrifuged at 2300 rpm for 5 min at 4 °C. Whole-cell lysates were re-suspended by prepared cold lysis buffer (50 mM Tris-HCl, pH = 7.4, 150 mM NaCl, 5 mM EDTA, 0.5% NP-40 supplemented with fresh protease inhibitors cocktail) for 1 h at 4 °C, centrifuged at 12,000 rpm for 15 min at 4°C. Five hundred micrograms of protein was then added and the incubation was continued for overnight at 4 °C. Beads were then washed five times using the lysis buffer, eluted in 2 × SDS loading buffer and boiling for 10 min. The boiled sample complexes were subjected to SDS-PAGE followed by immunoblotting with appropriate antibodies.

### Protein half-life assay

For HIF-2α half-life assay, cancer cell lines stably infected with indicated lentiviruses, or HEK293T cells transfected with indicated constructs were treated with the protein synthesis inhibitor cycloheximide (CHX, 100 μg/mL) for indicated time before harvesting. Immunoblotting was carried out to detect HIF-2α protein level.

### In vivo deubiquitylation assay

Cells transfected by indicated constructs for 48 h were subjected to in vivo ubiquitylation assays. After treated with the proteasome inhibitor MG132 (20 µM; Selleck) for 8 h, cells were collected in lysis buffer, then the lysates were pre-cleared by centrifugation at 12,000 rpm for 15 min. Whole-cell lysates (500 µg) were then incubated with HA beads overnight at 4 °C. After six times washes, western blot was carried out to detect ubiquitylated HIF-2α with anti-myc (or anti-Ub) monoclonal antibody.

### In vitro deubiquitylation assay

Flag-fusion deubiquitylation enzyme USP9X protein was purified from HEK293T cells, binding with Flag-M2 beads and eluted with FLAG peptides buffer. GST-tagged HIF-2α and HA-tagged ubiquitin were co-transfected into HEK293T cells for 48 h, and then treated with 20 μM MG132 for 8 h. HEK293T cells were then lysed with denaturing buffer (10 mM Tris-HCl, pH 8.0; 100 mM NaCl; 1 mM EDTA; 0.5% NP-40; 2% SDS; 20 mM NEM) freshly supplemented with protease inhibitor cocktail. The lysates were boiled for 10 min at 95 °C, diluted in NETN buffer and incubated with HA-agarose beads, then eluted with HA-peptide, and collecting elution binding with GST-4B sepharose were collected. Purified Flag-USP9X protein was then add into the same amounts of beads rotated in the reaction buffer (50 mM Tris-HCl, pH = 8.0; 50 mM NaCl, 1 mM EDTA; 10 mM DTT; 5% glycerol) for 8 h at 37 °C, and then the samples were eluted with 2× SDS loading buffer. Western Blot was carried out to detect ubiquitylated HIF-2α with anti-HA monoclonal antibody.

### GST pull-down assay

For GST pull-down assay, 2 µg GST-USP9X-N, C or D protein were incubated with 10 µg of 6×His-HIF-2α protein in binding buffer (50 mM Tris-HCl 7.5, 500 mM NaCl, 1% NP-40, 0.5 mM EDTA, 1 mM PMSF plus protease inhibitors (Roche) at 4 °C overnight, followed by an additional 1 h Glutathione Sepharose beads incubation. The beads were then washed six times and analyzed using SDS-PAGE and western blotting.

### Immunohistochemistry, immunofluorescence and PLA

Tissues were dissected, fixed in 4% paraformaldehyde (PFA) overnight. For immunohistochemistry, samples were embedded in paraffin and sectioned at 5 µm. Primarily sections were deparaffinized, rehydrated and subjected to antigen retrieval in either sodium citrate (pH = 6.0). After dislodging endogenous peroxidase with 3% H_2_O_2_, sections were permeabilized in 0.1% TritonX-100 for 30 min, and blocked in 10% goat serum for 1 h. Sections were incubated overnight at 4 °C in primary antibodies, washed in PBS-T, developed using DAB Substrate Kit (ZSGB BIO) and counterstained with Haematoxylin. Protein expression levels of all the samples were scored as five grades (negative, +, ++, +++, ++++) by multiplying the percentage of positive cells and immunostaining intensity. The percentage was scored as following: non-positive cells as 0 point, 1–30% as 1 point, 31–60% as 2 points, 61–80% and 81–100% as 3 and 4 points, respectively. Scoring was performed using a double-blind method. The intensity of staining was scored: no positive staining as 0 point, weak staining as 1 point, moderate staining as 2 points, strong staining as 3 points, and stronger staining as 4 points. The final scores were obtained according to above terms: 0 point was no expression, 1–3 was low expression, 4–8 was moderate expression, and 9–16 was high expression.

For immunofluorescence, tissues were embedded in paraffin and cells were fixed in 4% paraformaldehyde for 15 min. Samples, as described above, were incubated in primary antibodies, followed by incubation for 1 h at room temperature with secondary antibodies conjugated with different fluorochromes. Nuclei were counterstained with DAPI. Images were acquired with a Leica THUNDER Imager system.

For the PLA assays (Duolink), we followed the manufacturer’s instructions (Sigma, DUO92102) with minor modifications. In brief, cells were seeded in glass bottom cell culture dish. After 24 h, cells were washed with PBS and fixed with 4% PFA for 15 min at room temperature. Cells were then sequentially washed twice with PBS, permeabilized with 0.5% Triton X-100 for 10 min at room temperature, washed three times with PBS, blocked, incubated with antibodies at 4 °C overnight and processed according to the manufacturer’s instructions. Images were acquired with a Leica THUNDER Imager system. Red spots indicate protein–protein interactions, and blue indicates DAPI-stained nuclei.

### Orthotopic ovarian cancer model

Female mice (8 weeks of age) were anaesthetized by inhalation of isoflurane (5% in oxygen) in an induction chamber and anesthesia maintained at 2.5–3.0% isoflurane delivered via nosecone during all procedures. A small incision was made at the dorso-medial position directly above the ovarian fat pad, with a secondary small incision through the peritoneal wall. The ovarian fat pad was externalized and stabilized with a bull clip, and a dissecting microscope used to locate the oviduct in the exposed ovary. ID8-luc cells (1 × 10^6^) or different numbers of spheroid cells (1 × 10^5^, 1 × 10^4^ or 1 × 10^3^) were injected underneath the right ovarian bursa. The peritoneal wall was sutured closed using 6/0 suture and closure of the incision with surgical staples. Analgesia (Carprofen 5 mg/kg body weight) was provided in drinking water for 3 days thereafter. Mice were killed under experimental endpoints 6–8 weeks post-ID8 injection.

### In vivo imaging of mice

D-fluorescein solution was injected into mice intraperitoneally, 8 min later, mice were anaesthetized by inhalation of isoflurane (5% in oxygen) in an induction chamber and anesthesia maintained at 2.5–3.0% isoflurane delivered via nosecone during imaging. Images were acquired with a small animal in vivo imaging system (IVIS SPECTRUM, PE).

### Tumor patient-derived xenografts (PDX)

Six-eight-week-old female BALB/c nude mice were purchased from Beijing Vital River Laboratory Animal Technology Company (Vital River, Beijing, China). The patient’s tumor pieces were planted subcutaneously into the dorsal flanks of the mice (*n* = 6). Tumor volumes were measured approximately every 10 days and calculated according to the following formula: Volume (mm^3^) = 1/2 × length × width^2^. Mice were randomized prior to treatment. Mice were sacrificed and tumor tissues were collected after treatment completed. All procedures were approved by the Animal Care Committee of Tianjin Medical University.

### Primary cell culture

Cancer tissues obtained from HGSOC were minced in lysis buffer [1 mg/mL Collagenase I (Sigma), 0.05 mg/mL Dispase II (Sigma), 0.25 mg/mL Hyaluronidase (Sigma) and 0.01 mg/mL DNase I (Sigma) in DMEM: F12 (1:1) supplemented with 2% FBS] and shaked on the orbital shaker at 37 °C for 60 min. Dissociated tumor cells were filtered with 40 μm cell strainer (#352340, BD Falcon) and centrifuged at 500 × *g* for 5 min, then the pellet was resuspended in PBS, and washed 3 times. Then the isolated tumor cells were cultured in DMEM: F12 (1:1) supplemented with 10% FBS, 100 μg/mL streptomycin, 10 ng/mL epithelial growth factor (EGF), 5 μg/ml insulin, and 0.5 mg/mL hydrocortisone. Primary cells were used to perform stem cell and organoid formation experiments.

### Cell viability assay and calculation of IC50

The viability of cells exposed to the drugs was measured by CCK-8 assay. Cells were seeded in 96-wellplates at a density of 1000 cells/well, after overnight incubation, cells were treated with varying concentrations of drug for 72 h at 37 °C in an atmosphere of 5% CO_2_. After incubation, 10 μL of CCK-8 solution was added to each well and incubated at 37 °C for 2 h. The absorbance of formazan was measured at 450 nm using a microplate reader. The absorbance and logarithm of drug concentration were used for nonlinear fitting to calculate IC50 using GraphPad Prism 9 software.

### Wound-healing assay

The wound-healing assay was carried out according to a standard protocol using a sterilized 200 μL pipette tip to create a straight scratch on the cell monolayer. Cells were plated in 6-well plates and allowed to attach and form a cell monolayer. After creating the straight scratch, the cells were cultured in DMEM or RPMI 1640 medium supplemented with 2% FBS. The scratch closure in each group was observed under a microscope. The microscopy images at 0, 24, 48, 96 h were obtained. Image J software (version 3.2) was used to determine and calculate the wound-healing rate in each group.

### Sphere culture and assay

The cells were harvested and cultured in a stem cell culture medium in an ultralow attachment 6-well plate with a density of 1000 cells per well. The cell cultures supplemented with 10 ng/mL EGF, 2.5 g/mL amphotericin, 100 IU/mL penicillin, 100 g/mL streptomycin, and B-27 Supplement (50×) (Life Technologies, without serum). Fresh stem cell culture medium was added every two days. Spheroids were used to isolate mRNA or perform limiting dilution assays after treatment with Accutase (#40506ES60, Yeason).

### Organoid culture

Organoid culture was performed with High-Grade Serous Ovarian Cancer Organoid Kit (#K2167-HS, BioGenous, China), and follow the instructions. Briefly, the primary cell suspension and Matrigel (#354230, BioCoat) were mixed in a 1:1 ratio and placed on ice, seeded in the center of the 24-well plate as soon as possible, and then placed at 37 degrees for 30 min (2500 cells/well). After that, each well was supplemented with 500 μl of organoid medium, and the size of the organoids was observed after 5 days of culture, and when the diameter of the organoids was greater than 75 μm, drug treatment was performed after randomization. For USP9X-knocked primary cell organoid formation experiments, primary cells were infected with USP9X-knocked lentivirus and then organoid culture was performed.

### Transwell migration assay

For transwell assay, 8-μm Transwells (BD Biosciences) were used to detect the migration capacity of ovarian cancer cells. The cells were harvested by treatment with 0.25% trypsin, and resuspended in serum-free media. The lower chambers were filled with 200ul serum-free media, 4 × 10^4^ cells were seeded in each top chamber. The lower chambers were filled with 10% FBS complete media. Cell chambers were incubated for 24 h in a 37 °C, incubator supplemented with 5% CO_2_. Using a cotton swab remove the non- on the upper surface, and the invaded cells on the lower surface were fixed with methanol for 30 min and stained with the Crystal violet. Migrated cells on each insert were pictured with a microscope.

### Luciferase reporter construct and reporter activity assay

USP9X promoter (2192 bps) or TGF-β1 promoter (2072 bps) was cloned into pGL4.23 vector. HEK293T were seeded in 24-well plates and then co-transfected with different doses of Smad2 or Smad3 expression plasmids and pGL4.23-USP9X promoter after 48 h. For TGF-β1 promoter, HEK293T were co-transfected with HIF-1α, HIF-2α, or HIF-1α and HIF-2α, or incubated under 1% O_2_ for 48 h. Luciferase activities were determined with the Dual-Luciferase^®^ Reporter Assay System (Promega, USA).

### RT-qPCR

A quantitative real-time RT-PCR method was used to measure the amount of RNA. Briefly, total RNA was extracted using TRIzol (Invitrogen). Reverse transcription of RNA was performed using the RevertAid First Strand cDNA Synthesis Kit (Thermo Fisher Scientific) and qPCR was performed using SYBR Green Master Mix (Vazyme) according to the manufacturer’s instructions. Primers sequences are listed in Supplementary Table S1.

### RNA-Seq

TRIzol (Invitrogen) was used to isolate total RNA from cells. Total RNA samples were submitted for sequencing to the Genedenovo Biotechnology Co., Ltd (Guangzhou, China), and Illumina NovaSeq 6000 (Illumina). The sequence reads were mapped to mouse reference genome (GRCm38-mm10). StringTie v1.3.1 and DESeq2 were used to analyzed results with cutoff (p-value < 0.05, fold change>1.5).

### Chromatin immunoprecipitation (ChIP)

Approximately 5 × 10^7^ cells were used for each ChIP assay according to a previous study [29]. Chromatin was sonicated using a Bio-Red sonicator for several rounds on ice until the de-crosslinked DNA fragment was about 200–500 bp. Protein A/G beads was incubated with primary antibody for 8 h, and then incubated with the RIPA150 (50 mM Tris-HCl [pH = 8.0], 150 mM NaCl, 1 mM EDTA [pH = 8.0], 0.1% SDS, 0.5% Triton X-100) overnight at 4 °C. Immune complexes were then washed twice with RIPA150 buffer, twice with RIPA500 and once with TE buffer (10 mM Tris-HCl [pH = 8.0], 1 mM EDTA [pH = 8.0]). Then purified DNA was subject to qPCR assays with specific primers. The primers used were listed in Supplementary Table S1.

### Statistical analysis

All results are shown as the mean ± s.d. of multiple independent experiments. In vivo limiting dilution assays were performed with Extreme Limiting Dilution Analysis (ELDA, https://bioinf.wehi.edu.au/software/elda/index.html), the other statistical analyses were performed with GraphPad Prism 9 software. All statistical tests were two-sided, and *P* values < 0.05 were considered to be statistically significant.

## Results

### USP9X is synergistically enriched with TGF-β signaling in chemoresistant HGOSC and predicts poor progression

**Fig. 1:**
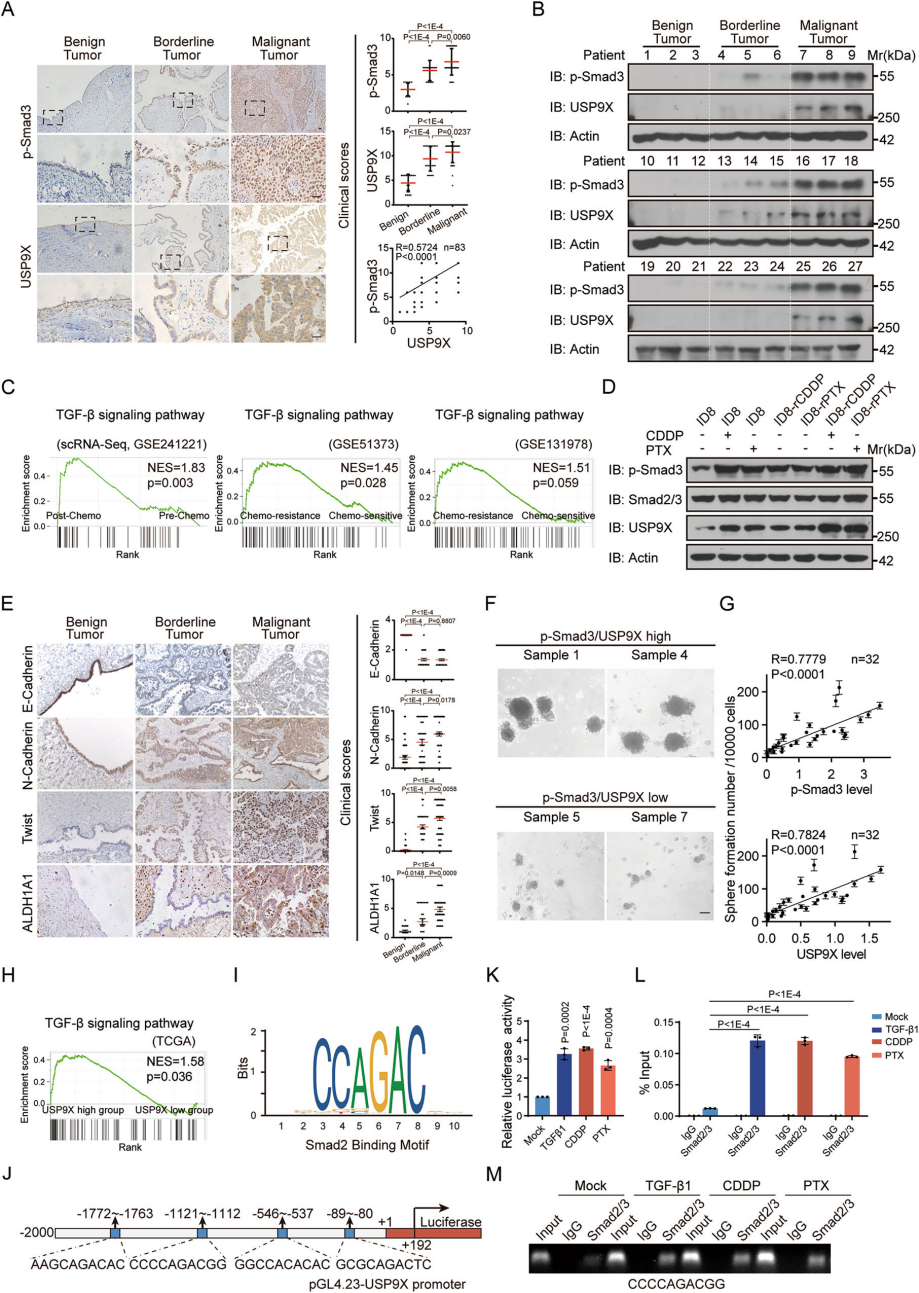
USP9X is highly correlated with TGF-β signaling during HGOSC progression and chemoresistance. **A** Representative images of p-Smad3 and USP9X IHC stainings on differently progressed HGSOC tissues (*n* = 83), including benign (*n* = 17), borderline (*n* = 25), and malignant tumors (*n* = 41). Scale bars, 50 µm. The images under are the zoom-in images for the upper images in each group. The right panels were clinical scores, and correlation analysis between p-Smad3 and USP9X. **B** Immunoblotting (IB) analysis of p-Smad3 and USP9X on freshly collected tumor tissues of serous OC with varying progression, including benign (*n* = 9), borderline (*n* = 9), and malignant tumors (*n* = 9). **C** GSEA of TGF-β signaling pathway in chemotherapy or platinum-resistant patients based on GSE241221 (scRNA-sequencing), GSE51373 and GSE131978. **D** IB analysis of p-Smad3, Smad2/3 and USP9X in ID8, chemoresistant cells ID8-rCDDP or ID8-rPTX with or without short treatment (24 h) of CDDP (10 μM) or PTX (5 nM). **E** Representative images of E-Cadherin, N-Cadherin, Twist, and ALDH1A1 IHC stainings on different progress HGSOC tissues (*n* = 83), including benign (*n* = 17), borderline (n = 25), and malignant tumors (*n* = 41). Scale bars, 50 µm. The right panels were clinical scores. **F** Representative images of spheroids in fresh HGOSC samples (*n* = 32). Spheroids larger than 50 μm in diameter were used for analysis. Scale bars, 75 µm. **G** Correlation analysis between spheroid numbers and p-Smad3 or USP9X expression based on (**F**). **H** GSEA analysis of TGF-β signaling pathway between USP9X high and low groups based on TCGA. Smad2 binding motif (**I**) and potential SREs on USP9X promoter (**J**) based on Jasper database. **K** Dual-luciferase assays were performed to analyze the transcriptional activation of USP9X promoter with TGF-β1 (5 ng/mL), CDDP (10 μM) or PTX (5 nM) for 24 h in CAOV3 cells. ChIP-qPCR was used to detect the Smad2/3 binding onto −1121 bp site of USP9X promoter, the % Input data was shown in (**L**) and the agarose gelatin plot was shown in (**M**). Data are shown as the mean ± s.d (**A**, **E**, **K**, **L**). *P* values were calculated by unpaired two-tailed Student’s *t* test (**A**, **E**, **K**, **L**). Correlation analysis was performed by Pearson correlation, *P* values (two-tailed) were calculated by Pearson *r* (**A**, **G**). *n* = 3 biological independent samples (**D**, **K**, **L**, **M**).

Based on the scattered reports about TGF-β signaling involvement in ovarian cancer (OC) progression and chemoresistance, we analyzed TCGA and GEO databases interactively to define the pathological significance. Indeed, TGF-β signaling activation is highly correlated with the progression and poor prognosis of HGSOC (Supplementary Fig. S1A). Consistently, pathological intensity of p-Smad3, as the direct readout of TGF-β signaling, was significantly elevated through malignant progression according to IHC profilings of our OC specimen cohort, composed mainly of HGSOC (Fig. 1A and Supplementary Fig. S1B). Blottings on freshly collected tumor tissues further showed that p-Smad3 achieved highest abundance in the malignant tissues, sharply contrasted to the neglectable level in benign tissues, while borderline tissues presented in between, indicating the critical oncogenic role of TGF-β signaling within HGSOC (Fig. 1B). More importantly, the gene set enrichment analysis (GSEA) showed that TGF-β signaling was enriched in patients received chemotherapy based on GSE241221 scRNA-seq dataset, and platinum-resistant patients based on GSE51373 and GSE131978 transcriptomic profiles (Fig. 1C). Across different OC cells, short applications of chemotherapeutic agents resulted in efficient p-Smad3 increase, corresponding to TGF-β signaling activation (Fig. 1D and Supplementary Fig. S1C). We were further curious whether long-term and repeated pulsatile exposure (short duration/high dose) of OC cells to chemotherapeutic agents, simulating the clinical therapy procedures administered to the patients, would also induce TGF-β signaling [30]. CDDP-resistant (rCDDP) and PTX-resistant (rPTX) ID8 cell lines were generated after six months of pulsatile exposure to cisplatin (CDDP) or paclitaxel (PTX), which achieved more than 5 times of IC50 increase, respectively, accompanying with acquired stemness as well as elevated drug transporters (Supplementary Fig. S1D, E). As expected, we observed dramatically increased p-Smad3 in the two lines of chemoresistant cells (Fig. 1D). Therefore, activated TGF-β signaling could participate in HGOSC chemo-resistance.

In accordance with the key function of USP9X in TGF-β signaling, USP9X was markedly elevated in response to chemotherapeutic treatments, pointing at its potential involvement (Fig. 1D and Supplementary Fig. S1C). Notably, the expression of USP9X was further increased in chemo-resistant cells upon CDDP or PTX re-treatment, which supports the significance of USP9X in tumor cells against chemotherapy (Fig. 1D). Indeed, Kaplan–Meier plotter (www.kmplot.com) dataset analyses showed that USP9X is not only more abundant in highly advanced and poorly differentiated HGOSC, but also correlated with poor prognosis in CDDP or PTX treated patients (Supplementary Fig. S1F, G). Additionally, the transcript level of USP9X was significantly correlated with TGF-β signature in OC (Supplementary Fig. S1H). Through our stained and blotted OC cohorts, USP9X was parallelly expressed with p-Smad3 and positively correlated with tumor grading (Fig. 1A, B and Supplementary Fig. S1B). Collectively, these bioinformatics and clinical findings support the key oncogenic role of USP9X during HGSOC development and chemoresistance, presumably integrated in TGF-β signaling.

As we mentioned, cancer stem cells (CSCs) contribute to clinical chemoresistance in ovarian cancer [4], we therefore asked whether the function of TGF-β in mediating chemoresistance in OC is attributed to its regulation of CSCs. Indeed, TGF-β signaling was enriched with CSCs in the GSEA study of ovarian cancer GSE25191 transcriptomic data (Supplementary Fig. S1I). On the same clinical cohort (Fig. 1A), EMT-related markers, particularly mesenchymal markers, Twist and N-cadherin, and CSC marker, ALDH1A1, showed synergistic expression tendencies with p-Smad3 and USP9X. Whereas, epithelial marker, E-cadherin, declined with tumor grading, sharply contrasting those of p-Smad3 and USP9X (Fig. 1E). Further, we obtained more direct evidences from the followed spheroid formation and capacity comparison study. Across about 40 patient samples, quantitatively we found the spheroid formation efficiencies were positively correlated with the p-Smad3, USP9X and CSC marker levels (Fig. 1F, G and Supplementary Fig. S1J–M). Together, these findings strongly confirmed that activated TGF-β signaling, probably through the key modulator USP9X, defines the stemness status during OC development and chemoresistance.

### TGF-β up-regulates USP9X expression through Smad2/3-mediated transcriptional regulation

In line with the expression and function compatibility between USP9X and p-Smad3, TGF-β signaling was significantly enriched in USP9X^high^ OC tissues in comparison with USP9X^low^ tissues (Fig. 1H). We therefore aimed to explore the mechanisms causing USP9X upregulation accompanying TGF-β signaling activation during OC development. Similar as observed in other lineages of malignant cells [22], TGF-β treatment augmented USP9X expression at both protein and mRNA levels in a dose-dependent manner (Supplementary Fig. S1N, O). Whereas, pharmacological inhibition of TGF-β with SB431542 functioned the opposite (Supplementary Fig. S1P, Q), supporting that USP9X is under TGF-β pathway control. As we know, p-Smad2/3 mediates transcriptional activation, post forming complex with Smad4 and translocating into the nuclei, thus transduces the main biological function downstream TGF-β pathway. We then transiently overexpressed Smad2 and Smad3 in ovarian cancer cells, CAOV3 and SKOV3, respectively, and found that only the overexpression of Smad2 increased the mRNA levels of USP9X (Supplementary Fig. S1R). We thus analyzed potential Smad response elements (SREs) on USP9X promoter through the Jasper database (Fig. 1I, J). Multiple SRE consensus sites were located at −1772 bp, −1121 bp, −546 bp, and −89 bp. Luciferase activity assay actually showed that overexpressed Smad2 activated USP9X promoter in a dose-dependent manner (Supplementary Fig. S1S). In addition, TGFβ1, CDDP or PTX treatment enhanced the promoter reporter activity of the USP9X in HGSOC CAOV3 cells (Fig. 1K). Further, endogenous ChIP experiments also showed that Smad2/3 were recruited to −1121 to 1112 bp site (CCCCAGACGG) in CAOV3 cells, which was even enhanced by TGFβ1, CDDP, or PTX treatments (Fig. 1L, M, and Supplementary Fig. S1T). Therefore, TGF-β signaling elevated during HGOSC progression and chemoresistance may activate USP9X expression to form functional cascade.

### USP9X mediates TGF-β-induced CSCs occurrence and chemoresistance

**Fig. 2:**
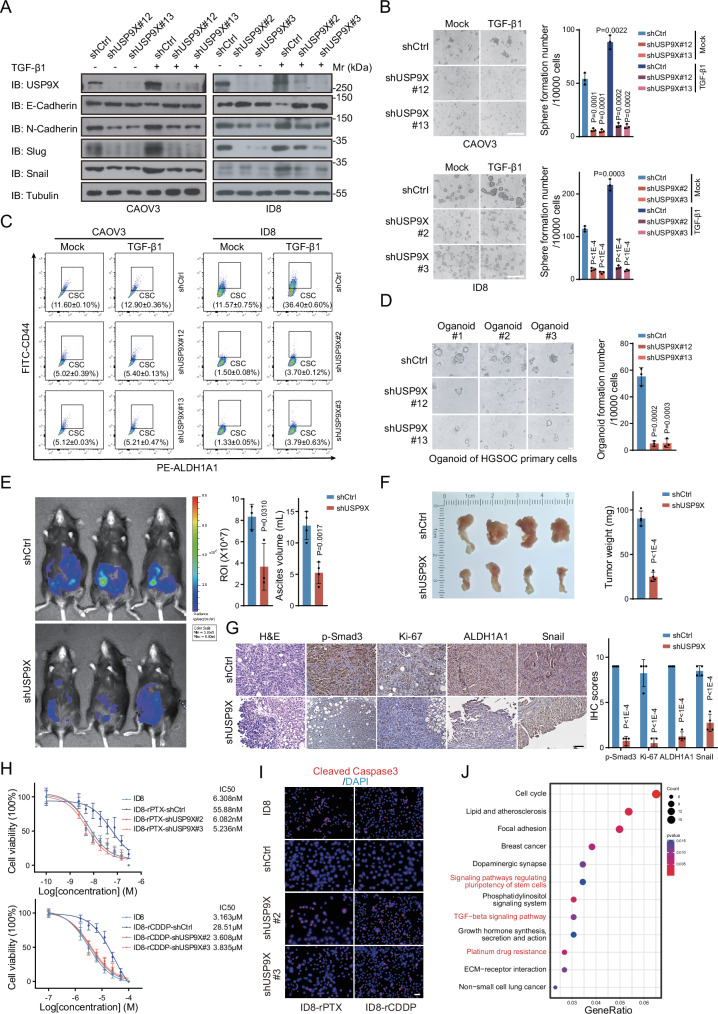
USP9X mediates TGF-β function in promoting CSCs occurance and chemoresistance. **A** IB analysis of USP9X, E-Cadherin, N-Cadherin, Slug, and Snail in USP9X knocked-down CAOV3 or ID8 cells treated with or without TGF-β1 (5 ng/mL), CAOV3 was treated for 96 h and ID8 was treated for 48 h from wound-healing assay (Fig. S2A). **B** Spheroid formation assay of USP9X knocked-down CAOV3 or ID8 cells treated with or without TGF-β1 (5 ng/mL) for 2 weeks. Scale bars, 100 µm. The right panels were the quantization chart. **C** FACS analysis of stem cell populations (ALDH1A1+/CD44+) in sphere-forming cells from USP9X knocked-down CAOV3 or ID8 cells treated with or without TGF-β1 (5 ng/mL) for 2 weeks. **D** Representative images of organoids formed in USP9X knocked-down primary HGSOC cells for 2 weeks. Organoids larger than 75 μm in diameter were used for analysis. The right panels were the quantization chart. Scale bars, 100 µm. **E** Luciferin bioluminescence signal intensities were monitored from orthotopic and intraperitoneal tumors in transplanted mice. *n* = 3 mice per group. The right panels were the quantization chart. **F** Orthotopic tumors from (**E**) were harvested and weighted, *n* = 4 mice per group. The right panels were the quantization chart. **G** Representative images of Hematoxylin and eosin (H&E), p-Smad3, Ki-67, ALDH1A1, and Snail IHC stainings on orthotopic tumors from (**F**). The right panels were the quantization chart. Scale bars, 50 µm. **H** Dose–response curve and IC50 values of ID8, ID8-rPTX (upper), ID8-rCDDP (below), USP9X knocked-down ID8-rPTXor ID8-rCDDP cells for 72 h. **I** Representative images of Cleaved-Caspase3 immunofluorescence (IF) stainings on ID8, ID8-rPTX, ID8-rCDDP, USP9X knocked-down ID8-rPTX or ID8-rCDDP cells. Scale bars, 50 µm. **J** KEGG pathway analysis based on USP9X knocked-down ID8 cells RNA-seq. Data are shown as the mean ± s.d (**B**–**H**). *P* values were calculated by unpaired two-tailed Student’s *t* test (**B**, **D**–**G**). *n* = 3 biological independent samples (**A**–**I**).

Next, cell behaviors were systematically analyzed to address the biological significance of USP9X within TGF-β signaling in terms of HGOSC development. First, USP9X is indispensable for HGOSC metastasis, exemplified with reduced cell migration through different assays upon USP9X knockdown (Supplementary Fig. S2A, B). As reported before, TGF-β1 treatment significantly promoted EMT activity and augmented cell migration, as important features of CSCs. While USP9X depletion inhibited TGF-β1-induced EMT function and counteracted the shifts of related molecular markers (Fig. 2A and Supplementary Fig. S2A, B). Moreover, TGF-β1 treatment promoted CSCs expansion, resulting in larger and more spheroids in the spheroid formation assay, with increased CSCs markers expression (Fig. 2B and Supplementary Fig. S2C). Fluorescence-activated cell sorting (FACS) assays also confirmed that the proportion of CSCs was increased dramatically upon TGF-β1 treatment (Fig. 2C and Supplementary Fig. S2D). However, USP9X knockdown greatly prohibited the function of TGF-β1 in promoting HGOSC CSCs occurrence, thus reduced CSC developing rates from about 90 (CAOV3) or 200 (ID8) spheres/10,000 cells to nearly none (Fig. 2B, C and Supplementary Fig. S2C, D). Back to clinical samples, organoid developing capability corresponds to CSCs function. Remarkably, the quantities of organoids derived from human OC primary cells were also significantly decreased by removing USP9X (Fig. 2D). Thus, USP9X may mediate the function of TGF-β1 on promoting CSCs occurrence and EMT transition in vitro.

In vivo, orthotopic tumor models were established via injecting the luciferase-expressing ID8 cells (ID8-luc) into the ovarian bursal cavity of C57BL/6 mice and visualized with Caliper IVIS Spectrum System. Compared with the ID8-luc cells, USP9X deficiency significantly reduced the bioluminescence signal intensities from orthotopic local and intraperitoneal tumors in transplanted mice (Fig. 2E and Supplementary Fig. S2E). Both the volumes and quantities of orthotopic and metastatic tumors in the peritoneum and small intestines were significantly reduced (Fig. 2F and Supplementary Fig. S2E). Defected tumor growth was accompanied with reduced cell proliferation, CSCs properties, EMT potentials, and TGF-β signaling activity (Fig. 2G). Therefore, USP9X is required for tumor development and metastasis in vivo, possibly due to its critical role downstream of TGF-β.

As aforementioned, CSCs are key mediators not only for cancer metastasis, also for drug resistance, we were thus curious whether USP9X participates in OC chemoresistance. Remarkably, USP9X knockdown recovered the IC50 values of CDDP-resistant and PTX-resistant ID8 cells to parental cell levels, and conferred the cells with sensitivities to CDDP or PTX (Fig. 2H, I and Supplementary Fig. S2F–I). The TGF-β signaling activity was simultaneously tampered, accompanied with attenuated EMT, CSCs, and drug resistance markers (Supplementary Fig. S2G). To further decipher the molecular mechanisms how USP9X integrates in TGF-β function within CSCs occurrence and chemoresistance, transcriptomes were profiled between USP9X knockdown and control ID8 cells. Consistent with the cell behaviors, Kyoto Encyclopedia of Genes and Genomes (KEGG) pathway analysis showed a clear down-regulation of the gene sets related to TGF-β pathway. Represented genes, such as Rock1, Bmpr2, Acvr2a, Thbs1, were significantly diminished in USP9X deficient cells (Fig. 2J). In addition, the gene sets for platinum resistance and CSCs-related pathways were also markedly reduced (Fig. 2J). Functionally and molecularly, these data indicate that USP9X mediates TGF-β function in promoting drug resistance in HGOSC, via maintaining CSCs.

### USP9X specifically interacts with HIF-2α

**Fig. 3:**
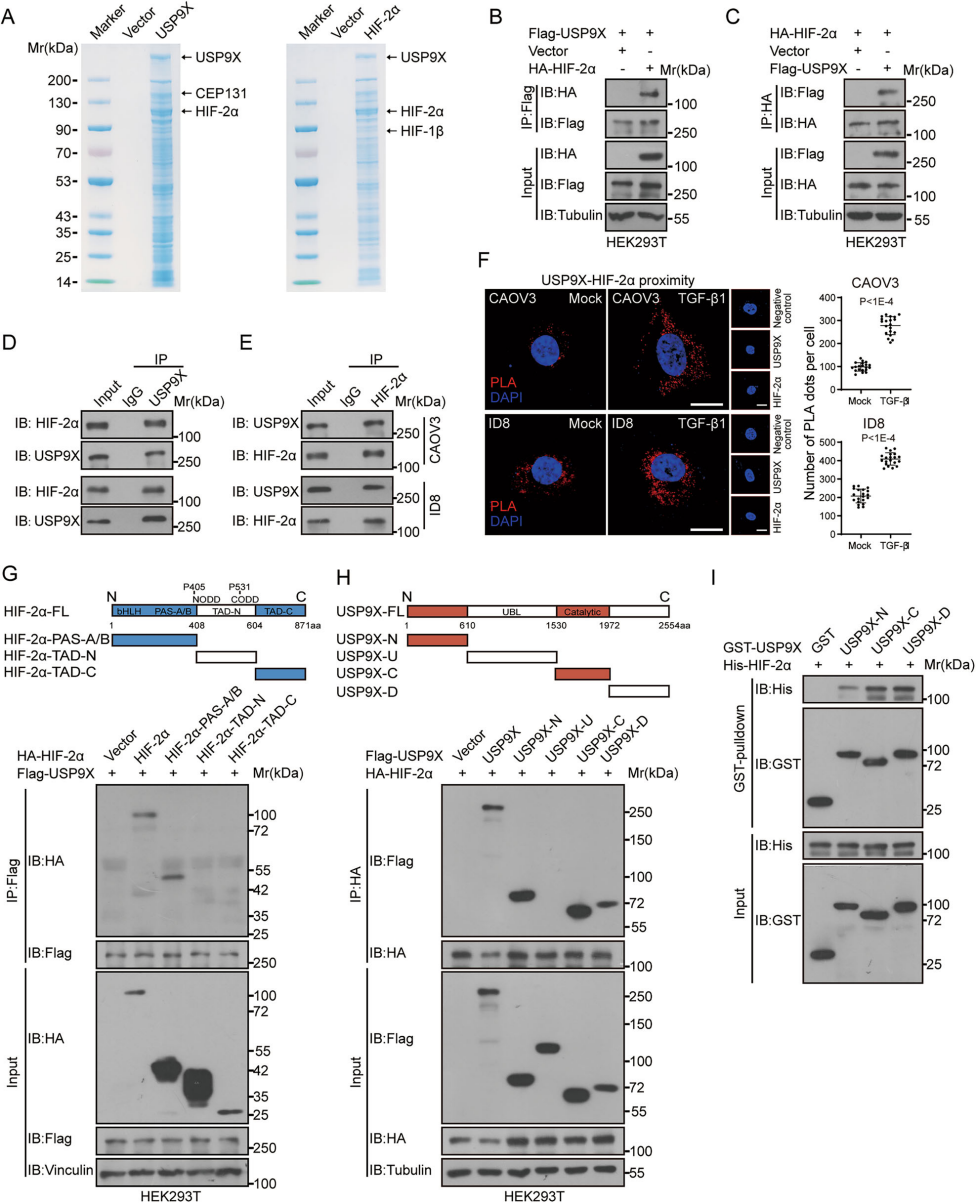
USP9X specifically interacts with HIF-2α in vivo and in vitro. **A** Affinity purification and mass spectrometry (AP-MS) was conducted on HEK293T cells over-expressing Flag-tagged-USP9X (left) and HIF-2α (right) to analyze USP9X and HIF-2α-associated protein complexes. **B**, **C** Exogenous Co-IP were performed to examine the interaction between over-expressed Flag-USP9X and HA-HIF-2α in HEK293T cells. **D**, **E** Co-IP were performed to validate endogenous binding of USP9X and HIF-2α in CAOV3 and ID8 cells. **F** PLA was performed to validate colocalization of USP9X and HIF-2α in CAOV3 and ID8 cells treated with or without TGF-β1 (5 ng/mL) for 24 h. Scale bars, 20 µm. The right panels were the quantization chart. **G** Co-IP were performed to check interaction between different HA-tagged-HIF-2α truncation mutants (PAS-A/B, TAD-N and TAD-C) and Flag-tagged-USP9X. **H** Co-IP were performed to check interaction between different Flag-tagged-USP9X truncation mutants (N, U, C and D) and HA-tagged-HIF-2α. **I** GST pull-down assay was performed to check interaction of purified GST-tagged domain of USP9X (N, C and D) and His-HIF-2α in vitro. Data are shown as the mean ± s.d (**F**). *P* values were calculated by unpaired two-tailed Student’s *t* test (**F**). *n* = 3 biological independent samples (**B**–**I**).

As an important DUB, USP9X regulates multi-types of cancer development, such as glioblastoma, lung cancer, colorectal cancer, and breast cancer, through mediating the stability of ALDH1A3, KDM4C, FBW7, CEP131, and Snail1 [24, 31–34]. To further explore the specifically targeted deubiquitylation by USP9X, we performed co-immunoprecipitation (Co-IP) coupled with Mass-spec using FLAG-tagged USP9X. Besides the previously identified DUB substrates such as CEP131, we found HIF-2α, the critical hypoxia mediator and tumor driver, was among the binding list of USP9X (Fig. 3A and Supplementary Fig. S3A). Accordingly, when HIF-2α was employed as the bait, USP9X was also efficiently pulled down through Co-IP Mass-spec, supporting their potent affinity (Fig. 3A and Supplementary Fig. S3A). The specific interaction between USP9X and HIF-2α was confirmed by Co-IP with exogenously transduced USP9X and HIF-2α (Fig. 3B, C and Supplementary Fig. S3B). More convincingly, endogenous HIF-2α was present in the USP9X immunoprecipitates from multi species-derived ovarian cancer cells (Fig. 3D, E and Supplementary Fig. S3C, D). We also adopted Proximity Ligation Assays (PLA) and immunofluorescence stainings (IF), which further showed that USP9X and HIF-2α were largely colocalized in the nuclei and cytoplasm across different ovarian cancer cells with or without TGF-β1 treatment, and the interaction between USP9X and HIF-2α was strengthened by TGF-β1 treatment (Fig. 3F, Supplementary Fig. S3E and Movies S1–10). These data convinced us that USP9X interacts with HIF-2α directly during the ovarian tumorigenesis, which could be a conserved molecular event across species.

Next, we searched for regions in USP9X and HIF-2α accounting for their interaction. Structurally, HIF-2α is composed of three functional domains, the bHLH (basic helix-loop-helix) region, two PAS (Per-ARNT-Sim) domains (PAS-A/B), N-terminal transcriptional activation domain (TAD-N) and a C-terminal transcriptional activation domain (TAD-C) (Fig. 3G). A series of truncation and immunoprecipitation experiments suggested that the PAS-A/B region (contains a bHLH region, two PAS domains) of HIF-2α plays the primary role for USP9X binding (Fig. 3G). USP9X, as a fairly big protein, utilizes different domains to bind with specific substrates. For instance, N-terminal sequences (amino acids 1–600) domain mediates the interaction with ALDH1A3 factor, amino acid 611 to 1,553 domain mediates the interaction with CEP131 factor. In this case, domain mapping experiments suggest that the USP9X-N (the N-terminus containing the α-α supercoil structure), USP9X-C (the ubiquitin-specific protease domain, contains DUB catalytic site C1566) and USP9X-D (the ubiquitin-specific protease domain) domains participate in the physical interaction with HIF-2α (Fig. 3H). Within in vitro GST pull-down system, USP9X-C and USP9X-D exhibited higher affinity to HIF-2α, indicating the C-terminal region undertakes the main binding activity (Fig. 3I). Therefore, specific functional domains within USP9X and HIF-2α mediate the direct molecular binding, which could be involved in ovarian cancer regulation.

### USP9X is a HIF-2α deubiquitinase

**Fig. 4:**
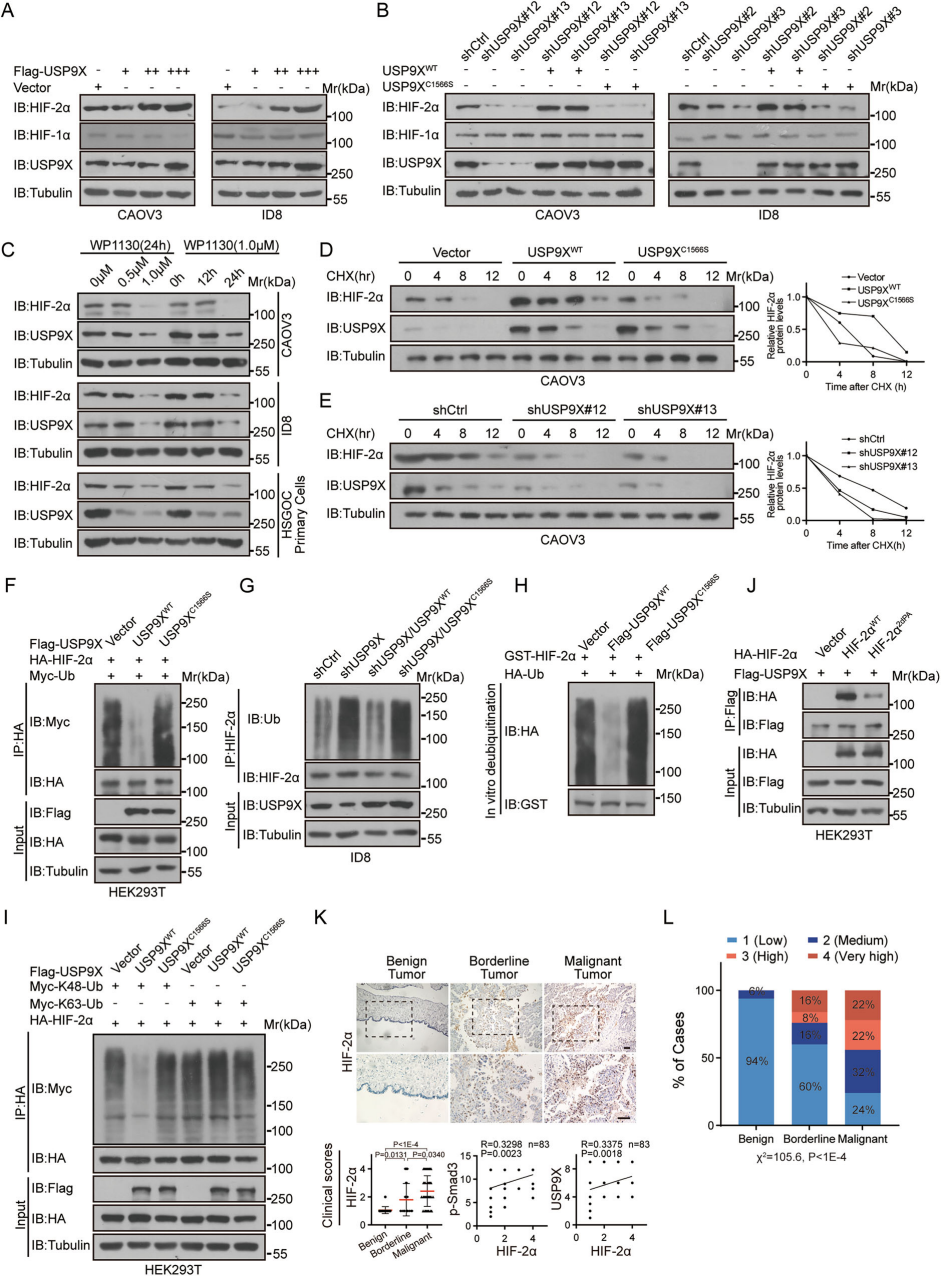
USP9X deubiquitylates and stabilizes HIF-2α. **A** HIF-2α expression was detected after over-expression of different doses of Flag-tagged-USP9X (1 µg, 2 µg or 3 µg) in CAOV3 and ID8 cells. **B** HIF-2α expression was detected after USP9X knocked down in CAOV3 and ID8 cells with or without wild-type (USP9X^WT^) or the catalytically dead USP9X (USP9X^C1566S^) re-expression. **C** CAOV3, ID8 and HGSOC primary cells were treated with different concentrations of WP1130 (USP9X inhibitor, 0, 0.5 or 1.0 µM) or 1.0 µM for different times (0, 12, or 24 h), and IB were used to detect HIF-2α expression. **D** Half-lives of HIF-2α were analyzed after over-expression of USP9X^WT^ or USP9X^C1566S^ in CAOV3 cells treated with the protein synthesis inhibitor cycloheximide (CHX, 100 μg/mL) for indicated time (0, 4, 8 or 12 h) before harvesting. **E** Half-lives of HIF-2α were analyzed after USP9X knocked down in CAOV3 cells treated with CHX (100 μg/mL) for indicated time (0, 4, 8 or 12 h) before harvesting. **F** Ubiquitylation analyses of HA-tagged-HIF-2α after co-transfection with Flag-tagged-USP9X^WT^ or USP9X^C1566S^ and Myc-tagged-ubiquitin (48 h). **G** Ubiquitylation analyses of endogenous HIF-2α in USP9X knocked-down of ID8 cells with or without Flag-tagged-USP9X^WT^ or USP9X^C1566S^ re-expression. **H** In vitro deubiquitylation analyses on GST-tagged-HIF-2α incubated with Flag-tagged-USP9X^WT^ or mutant USP9X^C1566S^ as well as HA-tagged-ubiquitin. **I** In vivo deubiquitylation analyses on HIF-2α conjugated with indicated ubiquitin chains and incubated with Flag-tagged-USP9X^WT^ or USP9X^C1566S^ in HEK293T cells. **J** Co-IP to determine the interaction between Flag-tagged-USP9X and HA-tagged-HIF-2α^WT^ or the P405A/P531 A (2dPA) HIF-2α mutant that lacks hydroxylation. **K** Representative images of HIF-2α IHC stainings on differently progressed HGSOC tissues (*n* = 83), including benign (*n* = 17), borderline (*n* = 25), and malignant tumors (*n* = 41), the same cohort as in Fig. 1A. The images below were a zoom in on the image above. Scale bars, 50 µm. The panels below showed clinical scores of HIF-2α, and correlation analysis between HIF-2α and p-Smad3 or USP9X. **L** The HIF-2α IHC staining results of serous OC patients were scored and the histogram showed the percentage of cases, statistically analysis was performed by χ^2^-test. Data are shown as the mean ± s.d, *P* values were calculated by unpaired two-tailed Student’s t test **K**. Correlation analysis was performed by Pearson correlation, P values (two-tailed) were calculated by Pearson r (**K**). *n* = 3 biological independent samples (**A**–**I**).

To explore whether USP9X accounts for the stability of HIF-2α protein, deubiquitylation and stability assays were performed under normoxic conditions for convenience. Indeed, USP9X overexpression increased HIF-2α levels in a dose-dependent manner, without affecting the HIF-1α levels which has been reported as a USP9X substrate in breast cancer [35] (Fig. 4A and Supplementary Fig. S4A). However, HIF-2α level was markedly reduced upon independent USP9X knockdown through lentivirus-mediated shRNA (Fig. 4B). This stabilization of HIF-2α depends on USP9X’s DUB activity, since wild-type USP9X, but not the DUB-dead mutant USP9X^C1566S^, restored HIF-2α loss caused by USP9X depletion (Fig. 4B). Moreover, pharmaceutic repression of USP9X DUB activity with specific inhibitor WP1130 decreased HIF-2α protein efficiently as well (Fig. 4C). MG132 treatment similarly restored HIF-2α loss caused by USP9X knockdown (Supplementary Fig. S4B). The observed expression changes corresponded to prolonged or shortened half-life of HIF-2α upon USP9X overexpression or depletion/inhibition (Fig. 4D, E and Supplementary Fig. S4C–F). Indeed, the USP9X^C1566S^ mutant lost the function to prolong HIF-2α half-life (Fig. 4D, E and Supplementary Fig. S4C–E). These experiments together suggest that the DUB USP9X stabilizes HIF-2α likely by counteracting UPS-mediated degradation.

We next verified whether USP9X targets HIF-2α for deubiquitylation. As expected, ectopic expression of wild-type USP9X but not USP9X^C1566S^ reduced the poly-ubiquitylation of HIF-2α, while USP9X knockdown or repression augmented HIF-2α poly-ubiquitylation (Fig. 4F, G). Similar as the half-life analyses, ectopic expression of wild-type USP9X removed the elevated poly-ubiquitylation from HIF-2α caused by USP9X depletion, while USP9X^C1566S^ failed to do so (Fig. 4G). Importantly, wild-type USP9X, but not USP9X^C1566S^, purified from HEK293T cells removed the ubiquitin chain from recombinant HIF-2α in vitro (Fig. 4H). K48-linked and K63-linked poly-ubiquitylations are common ubiquitylation events, wild-type USP9X, but not DUB dead USP9X^C1566S^, efficiently removed K48-linked poly-ubiquitylation from HIF-2α, which is the canonical signal that marks proteins for degradation (Fig. 4I). The 2dPA (P405A/P531A) residues of HIF-2α, necessary for binding with the ubiquitin E3 ligase VHL [36], are located within N-and C-terminal oxygen-dependent degradation domains (NODD and CODD), mutation of which indeed abrogated the interaction with USP9X (Figs. 3G and 4J). Therefore, USP9X may deubiquitylate HIF-2α that has undergone E3 ligase VHL-mediated ubiquitylation.

To validate the clinical significance of USP9X stabilized HIF-2α, we determined the expression of HIF-2α on the same cohort of OC specimens as for USP9X analysis. Similar as USP9X, HIF-2α abundance was increased with tumor progression, that is malignant > borderline > benign, and HIF-2α preferred to be distributed in the area of papillary or stratifying structure far from vascular vessels (Fig. 4K, L and Supplementary Fig. S4G, H). Remarkably, the distribution and intensity of HIF-2α correlates positively with USP9X, as well as p-Smad3 (Fig. 4K). These findings indicate that the pathological increase of USP9X, promoted by TGF-β-p-Smad2/3 possibly, could result in elevated HIF-2α.

### USP9X promotes CSCs occurrence by stabilizing HIF-2α

**Fig. 5:**
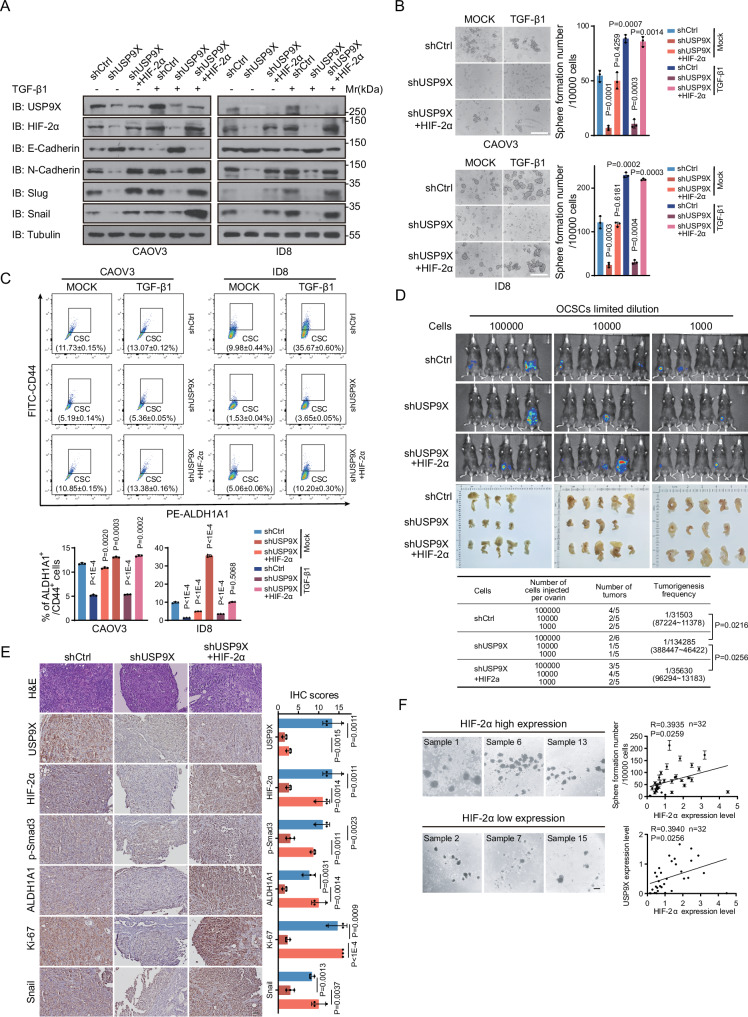
USP9X promotes CSCs function in a HIF-2α-dependent manner. **A** IB analysis of USP9X, HIF-2α, E-Cadherin, N-Cadherin, Slug and Snail in USP9X knocked-down and HIF-2α overexpressed CAOV3 or ID8 cells treated with or without TGF-β1 (5 ng/mL), CAOV3 was treated for 96 h and ID8 was treated for 48 h from wound-healing assay (Fig. S5A). **B** Spheroid formation assay of USP9X knocked-down and HIF-2α overexpressed CAOV3 or ID8 cells treated with or without TGF-β1 (5 ng/mL) for 2 weeks. Scale bars, 100 µm. The right panels were the quantization chart. **C** FACS analysis of stem cell populations (ALDH1A1+/CD44+) in sphere-forming stem cells from USP9X knocked-down and HIF-2α overexpressed CAOV3 or ID8 cells treated with or without TGF-β1 (5 ng/mL) for 2 weeks, The lower panels were the quantization chart. **D** Luciferin bioluminescence signal intensities from orthotopic and intraperitoneal tumors in USP9X knocked-down and HIF-2α overexpressed ID8-luc mouse model by limited dilution assays. Tumorigenesis frequency statistics were performed using the ELDA (Extreme Limiting Dilution Analysis) method. n ≥ 5 mice per group. (**E**) Representative images of H&E, USP9X, p-Smad3, HIF-2α, ALDH1A1, Ki-67 and Snail IHC stainings on orthotopic tumors from (**D**). The right panels were the quantization chart. Scale bars, 50 µm. **F** Representative images of spheroids of freshly HGOSC samples (*n* = 32), the same cohort as in Fig. 1F. Scale bars, 75 µm. And right panel showed correlation analysis between HIF-2α and spheroid formation number or USP9X. Data are shown as the mean ± s.d (**B**, **C**, **E**, **F**). *P* values were calculated by unpaired two-tailed Student’s *t* test (**B**, **C**, **E**). Correlation analysis was performed by Pearson correlation, *P* values (two-tailed) were calculated by Pearson *r* (**F**). *n* = 3 biological independent samples (**A**–**C**, **E**).

Based on the direct regulation of USP9X on HIF-2α stability, as well as the crucial role of HIF-2α in coordinating hypoxic tumor microenvironment and CSCs maintenance [7, 36, 37], we hypothesized that USP9X might enhance CSCs occurrence through maintaining HIF-2α. Indeed, knockdown of HIF-2α in ID8 cells reduced TGF-β-induced cell migration (Supplementary Fig. S5A, B). On the contrary, re-expressed HIF-2α efficiently recovered the tumor cell migration, spheroid formation efficiency and CSCs proportion defects caused by USP9X knockdown, respectively (Fig. 5A–C and Supplementary Fig. S5C, D). Expression of EMT and CSCs markers were also rescued (Fig. 5A, and Supplementary Fig. S5E). In vivo, the significance of USP9X-HIF-2α axis was evaluated by limiting dilution assays, conducted on ID8-luc mouse model. Consistent with the functions in vitro and in bulk cell population (Fig. 5A–C and Fig. 2E), within both in vivo imaging and anatomical assays, USP9X deprivation suppressed the tumor regeneration and intraperitoneal metastasis capability of CSCs. Notably, HIF-2α overexpression reversed the defects and led tumor formation to parental ID8 CSCs level (Fig. 5D). Even in the bioluminescence-responsive ovarian tissues from USP9X knockdown animals, the malignant nodules were shrunk to hardly detectable levels, as well as the expression of HIF-2α, p-Smad3, EMT/proliferation markers (Fig. 5E). However, re-introduction of HIF-2α significantly restored the tumor initiation and metastasis of USP9X-null CSCs, accompanied with elevated markers (Fig. 5D, E). Collectively, these data confirmed the critical function of HIF-2α in mediating USP9X-promoted OC development and CSCs maintenance through multiple cell and mouse models.

Clinically, HIF-2α abundance significantly correlates with the CSCs occurrence efficiency across different patients-derived primary OC samples, as well as with EMT tendency (Fig. 5F and Supplementary Fig. S5F), which reminisces USP9X function (Figs. 1 and 2). Correlation analyses further showed that USP9X and HIF-2α were both highly expressed and positively correlated with malignancy grade and ascites metastasis (Fig. 4G). Moreover, high levels of HIF-2α transcripts were associated with decreased OS (HR = 1.23, *P* = 0.012) and PFS (HR = 1.19, P = 0.035) in serous ovarian cancer, even other types of ovarian cancers, based on the Kaplan–Meier plotter (www.kmplot.com) analysis (Supplementary Fig. S5G). Similar as previously reported [7], HIF-2α may account for the chemoresistance, since reduction of HIF-2α positive proportion of CSCs occurred simultaneously with regained cell apoptosis so as to sensitivities to chemotherapeutic drugs within USP9X-knockdown CDDP and PTX-resistant cancer cells (Fig. 2, and Supplementary Fig. S5H). From in vitro to clinical validation, we conclude that USP9X-HIF-2α regulation defines CSCs state during OC development, thus may predict poor prognosis and chemoresistance.

### USP9X coordinates TGF-β and hypoxia-induced CSCs functions

**Fig. 6:**
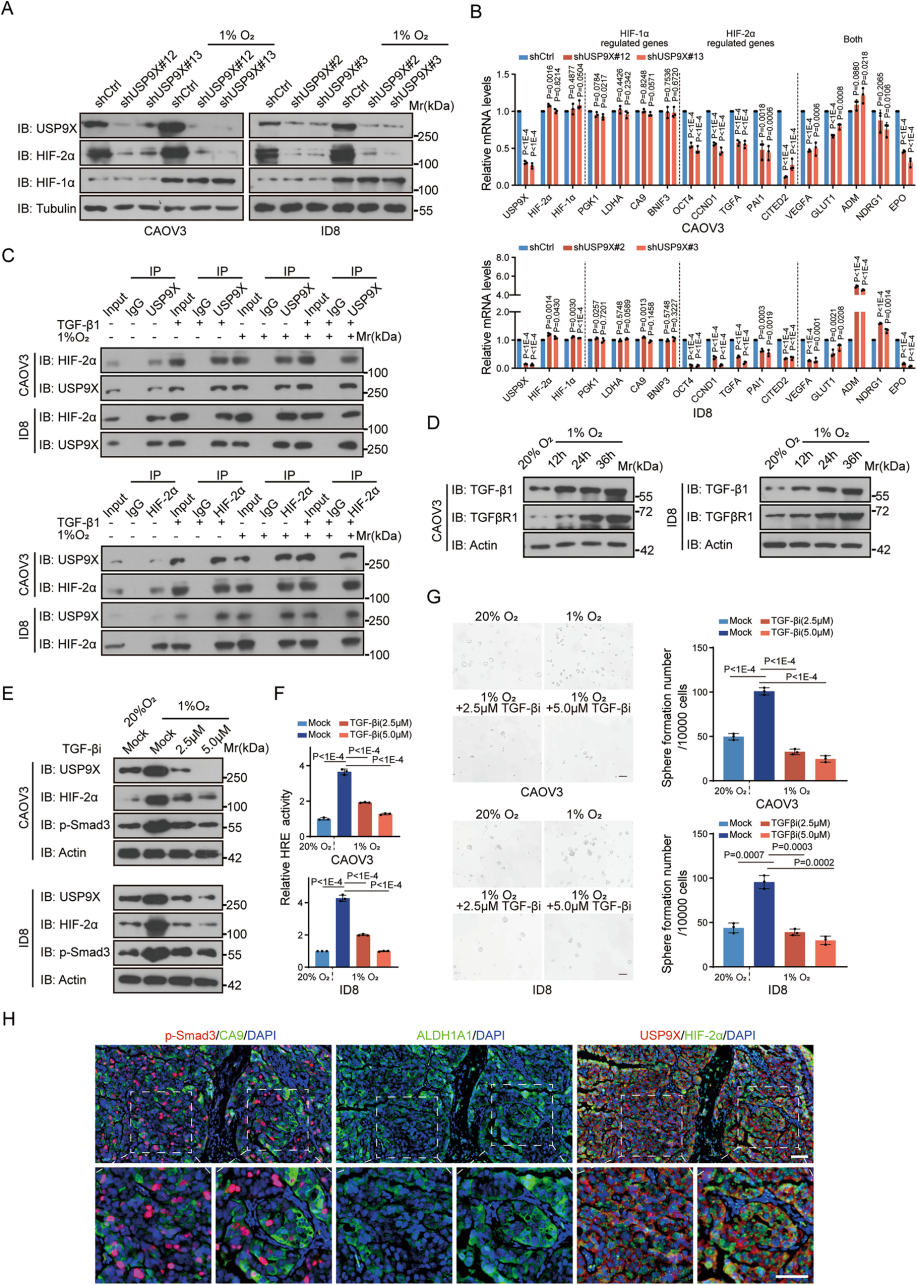
USP9X-HIF-2α axis mediates TGF-β and hypoxia-induced CSCs function. **A** IB analysis of USP9X, HIF-2α and HIF-1α in USP9X knocked-down CAOV3 or ID8 cells incubated under 1% O_2_ for 24 h. **B** HIF-1α or HIF-2α-specific target genes (PGK1, LDHA, CA9, BNIF3 for HIF-1α. OCT4, CCND1, TGFA, PAI1, CITED2 for HIF-2α), as well as common target genes (VEGFA, GLUT1, ADM, NDRG1, EPO) mRNA expression was detected by qRT-PCR in CAOV3 or ID8 cells incubated under 1% O_2_ for 24 h. **C** Co-IPs were performed to validate endogenous binding of USP9X and HIF-2α in CAOV3 and ID8 cells treated with TGF-β1 (5 ng/mL) and incubated under 1% O_2_ for 24 h. **D** IB analysis of TGF-β1 and TGFβR1 in CAOV3 or ID8 cells incubated under 1% O_2_ for different time (0, 12, 24 or 36 h). **E** IB analysis of USP9X, HIF-2α and p-Smad3 in CAOV3 or ID8 cells incubated under 1% O_2_ with or without TGF-β inhibitor (SB431542, 2.5 µM or 5.0 µM) for 24 h. **F** Hypoxia-related elements (HRE) luciferase assays were performed to analyze the activity of hypoxic signaling pathways in CAOV3 or ID8 cells incubated under 1% O_2_ with or without TGF-β inhibitor (2.5 µM or 5.0 µM) for 24 h. (**G**) Spheroid formation assay of CAOV3 or ID8 cells incubated under 1% O_2_ with or without TGF-β inhibitor (2.5 µM or 5.0 µM) for 48 h. Scale bars, 100 µm. The right panels were the quantization chart. **H** IF staining of the hypoxia marker CA9, p-Smad3, ALDH1A1, USP9X and HIF-2α on serial HGSOC tissue sections. Scale bars, 50 µm. Data are shown as the mean ± s.d (**B**, **F**, **G**). P values were calculated by unpaired two-tailed Student’s *t* test (**B**, **F**, **G**). *n* = 3 biological independent samples (**A**–**H**).

As we know, hypoxic microenvironment achieves high HIF-2α stability via PHD-dependent hydroxylation, thus escape from VHL-mediated ubiquitylation, to promote CSCs development [7, 36, 37]. Here, we define USP9X as the deubiquitinase of HIF-2α through CSCs behaviors, we therefore wonder whether USP9X-mediated stabilization of HIF-2α is a hypoxia-responsive event. Interestingly, both the mRNA and protein level of USP9X was significantly induced by oxygen deprivation (1% oxygen), in parallel with increased HIF-1α and HIF-2α (Supplementary Fig. S6A). In contrast, protein level of HIF-2α could no longer be induced by hypoxic condition once USP9X was knocked-down, which blocked hypoxia signaling activity (Fig. 6A and Supplementary Fig. S6B). Consistent with aforementioned findings (Fig. 4A and Supplementary Fig. S4A), the protein level of HIF-1α was not significantly affected by USP9X loss. Even HIF-2α and HIF-1α share the same DNA consensus motifs for transactivation, they do show preference in terms of target recognition. We actually noticed reduced HIF-regulated genes upon USP9X loss concentrated more on HIF-2α targets, apart from a small number of common targets **(**Fig. 6B). Therefore, in response to hypoxia, USP9X participates in hypoxic signaling through, mainly, stabilizing HIF-2α.

Considering the above findings under hypoxia, with the USP9X function under TGF-β-induced CSCs occurrence, the coming question would be how these two pivotal signalings converge. In fact, TGF-β augmented HIF-2α level dramatically in addition to hypoxia single treatment, with slight synergistic effect though (Fig. 6C). Consequently, the interaction between USP9X and HIF-2α was strengthened by either hypoxia or TGF-β or both, which may assist HIF-2α accumulation since high binding means high stability (Supplementary Fig. S6C). For a while, the high TGF-β in the tumor microenvironment of OC has been realized to be a pivotal driver for CSCs occurrence and metastasis [4, 10], but the origin of TGF-β is not quite clear. Interestingly, we found that hypoxia led to TGF-β increase cross different OC cell lines at both the mRNA and protein level, as well as the signaling components such as TGF-β type I receptor (TGFβRI), p-Smad3, and USP9X in a time-dependent manner (Fig. 6D and Supplementary Fig. S6D). Followed motif and function analyses of promoter region actually confirmed that hypoxia pathway transcriptionally activates the TGF-β1 expression (Supplementary Fig. S6E, F). Thus, hypoxic microenvironment activates autocrine TGF-β pathway to up-regulate USP9X, the DUB required for HIF-2α stabilization. Antagonizing the regulatory axis by USP9X knockdown or TGFβRI inhibitor blocked hypoxia-induced HIF-2α expression, so as to downstream transactivation (Fig. 6E, F). Moreover, TGF-β1 expression and signaling activity (represented with Smad Element Response activity, SER) could no longer be induced by hypoxic condition once USP9X was knocked down (Supplementary Fig. S6G, H). Functionally, CSC spheroid formation rates failed to respond to hypoxia with the presence of TGFβRI inhibitor (Fig. 6G). Notably, the latest single-cell RNA sequencing studies of HGSOC showed that the hypoxia signaling pathway was significantly upregulated in tumor cells of patients undergoing chemotherapy in parallel with TGF-β signaling (Fig. 1C, Supplementary Fig. S6I, J) [14, 38]. Together, these evidences indicate that hypoxic tumor microenvironment of OC may be the underlying driver for TGF-β secretion and TGF-β signaling activation [39–41], which, in turn, leads to highly expressed USP9X, stabilized HIF-2α and activated CSCs.

To further verify the hypoxia-induced upregulation of TGF-β-USP9X-HIF-2α axis in CSCs of human HGSOC, we performed immunofluorescent staining of USP9X, the hypoxia marker CA9, and the CSCs marker ALDH1A1 on serial tissue sections. First, we observed largely co-localized USP9X and HIF-2α, especially in the CA9-marked hypoxic region (Fig. 6H). Even only a fraction of CA9- or ALDH1A1-positive cells showed USP9X staining, the majority of CA9/ALDH1A1 double-positive cells had strong USP9X as well as HIF-2α stainings, indicating that USP9X-HIF-2α was upregulated in those CSCs under hypoxia in human HGSOC (Fig. 6H). Also, tumor cells within hypoxic areas usually have activated TGF-β labeled by p-Smad3. These data indicate that hypoxia-activated TGF-β-USP9X-HIF-2α regulatory axis functions in clinical HGSOC CSCs maintenance.

### Targeting USP9X could be a promising therapeutic strategy against HGSOC

**Fig. 7:**
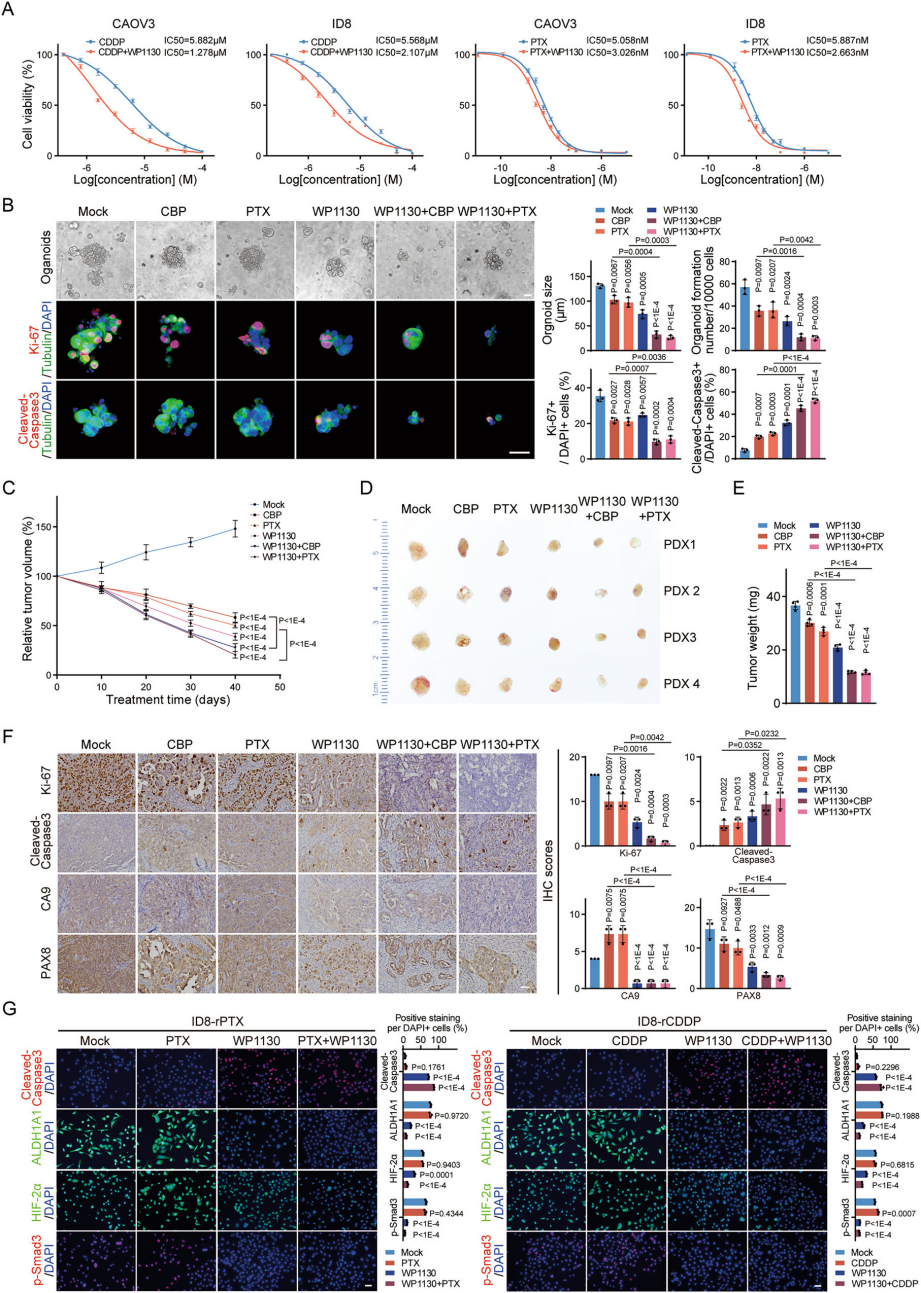
Targeting USP9X overcomes chemoresistance of HGSOC. **A** Dose–response curve and IC50 values of CAOV3 and ID8 cells treated with WP1130 (1 µM) and different concentrations of CDDP or PTX for 72 h. **B** Representative images of whole-mount IF stainings on primary HGSOC cells-derived organoids, treated with WP1130 (1 µM), CBP (1 µM) or PTX (1 nM) for 72 h. The right panels were the quantization chart. Scale bars, 100 µm. **C** Tumor growth curves for HGSOC PDX models treated with WP1130 (30 mg/kg), CBP (60 mg/kg) or PTX (20 mg/kg) through intraperitoneal injection after palpable tumor formed. 1 time/3 days, 12 cycles, n = 4 mice per group. **D**, **E** Tumors of PDX models in (**C**) were harvested and weighted (**E**) after therapies with CBP, PTX or WP1130. *n* = 4 mice per group. **F** Representative images of Ki-67, Cleaved-Caspase3, CA9 and PAX8 (HGSOC marker) IHC stainings on tumors of (**D**). Scale bars, 50 µm. The right panels were the quantization chart. **G** Representative images of Cleaved-Caspase3, ALDH1A1, HIF-2α and p-Smad3 IF stainings on ID8-rPTX and ID8-rCDDP cells treated with WP1130 (1 µM), CDDP (1 µM) or PTX (1 nM) for 24 h. Scale bars, 50 µm. The right panels were the quantization chart. Data are shown as the mean ± s.d (**A**–**C,** **E**–**G**). *P* values were calculated by unpaired two-tailed Student’s *t* test (**A**–**C,** **E**–**G**). *n* = 3 biological independent samples (**A**, **B**, **F**, **G**).

Based on the function of USP9X across TGF-β and hypoxia signalings, CSCs maintenance and resultant chemoresistance, we wonder whether targeting USP9X would meliorate the therapeutic effect and drug response of first-line chemotherapy agents in HGSOC. CAOV3 and ID8 cells were treated with cisplatin (CDDP) or paclitaxel (PTX) alone or with USP9X inhibitor WP1130 simultaneously. Encouragingly, USP9X inhibitor conferred the ovarian cancer cells higher sensitivity to chemotherapeutic agents, and reduced the IC50 significantly (CDDP: IC50: 5.882 μM vs. 1.278 μM for CAOV3; 5.568 μM vs. 2.107 μM for ID8; PTX: IC50: 5.058 nM vs. 3.026 nM for CAOV3, 5.887 nM vs. 2.663 nM for ID8.) (Fig. 7A and Supplementary Fig. S7A). Repression of CSCs function represented with reduced EMT and CSCs markers may account for improved chemosensitivity (Supplementary Fig. S7B). Thus, antagonizing USP9X improves HGSOC cell chemosensitivity efficiently via restricting CSCs contents and function.

To further validate our findings from a translational perspective, a patient-derived organoid culture system was employed to investigate the therapeutic potential of a USP9X inhibitor in the treatment of HGSOC. Compared to carboplatin (CBP) or PTX single treatment, WP1130 suppressed the organoids growth effectively (Fig. 7B and Supplementary Fig. S7C). More encouragingly, combined therapies (WP1130 together with CBP or with PTX) inhibited organoids growth much more efficiently than any drug alone (Fig. 7B and Supplementary Fig. S7C). By analyzing the molecule expressions from different treatment groups by whole-mount IF, we observed reduced Ki-67 positive cells and increased Cleaved-Caspase 3 cells in each therapy group. The USP9X and HIF2α positive cells were significantly decreased in WP1130 and combined therapy groups, rather than CBP or PTX single treatment groups (Fig. 7B and Supplementary Fig. S7C). These results indicate that USP9X inhibitor may effectively promote HGSOC cell apoptosis and sensitivity to clinical first-line chemotherapy agents, presumably through interfering USP9X-HIF2α-CSCs regulation axis.

The inspiring therapy effect of USP9X inhibitor in vitro promoted us to explore the in vivo efficacy. Firstly, we constructed HGSOC patient-derived xenograft (PDX) nude mice models and employed similar therapy strategies as for organoids by subcutaneously injections. Very excitingly, combined therapy inhibited tumor growth much more effectively than either WP1130, CBP or PTX alone (Fig. 7C–E). Molecular expression signatures were analyzed by IHC. Similar to the organoid therapies, repressed cell proliferation and increased cell apoptosis were achieved in all therapy groups, while diminished USP9X, CA9, and HIF2α positive cells mainly occurred in WP1130 or combined therapy groups (Fig. 7F and Supplementary Fig. S7D). Consistent with the extremely elevated sensitivity to PTX in vitro, WP1130 combined with PTX treatment prevented the tumors from expansion and resulted in hardly detectable nodules. PAX8, as a clinical pathological marker for HGSOC determination, showed neglectable expression as well as cellular localization switch under efficacious combined therapies (Fig. 7F and Supplementary Fig. S7D). Collectively, targeting of USP9X may potentially be an effective treatment for HGSOC, which could also improve chemotherapy response in vivo.

**Fig. 8:**
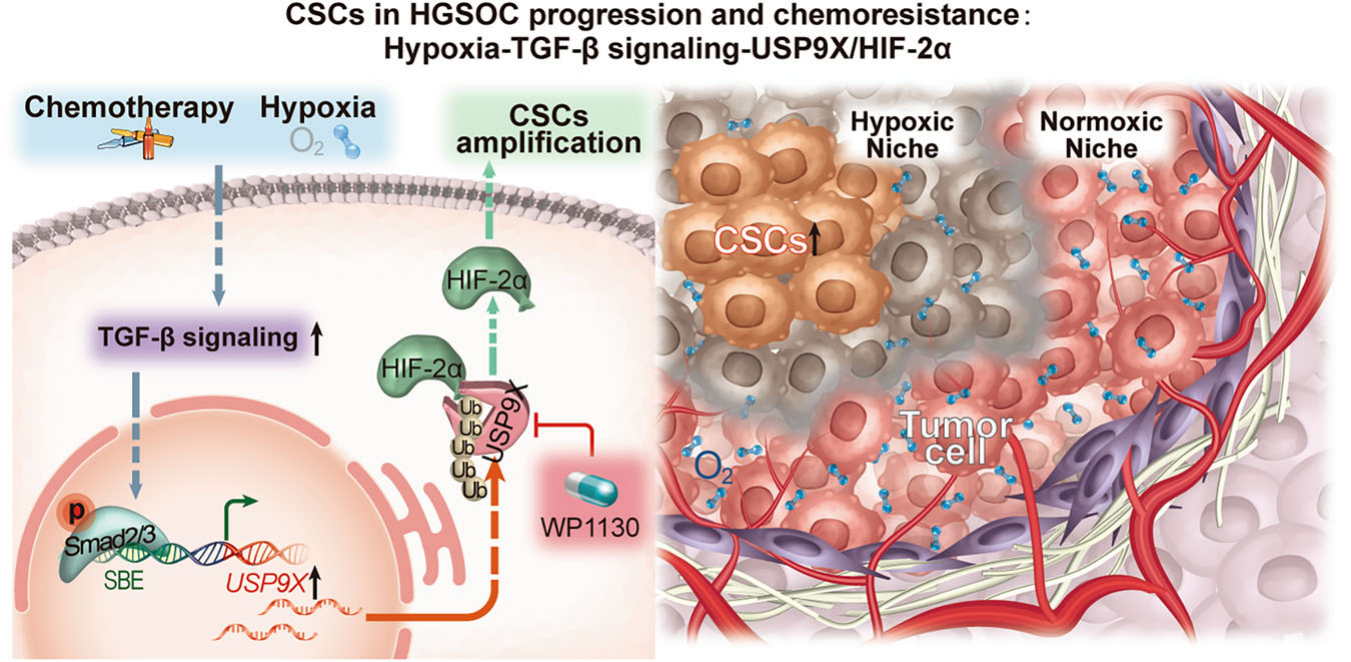
Scheme depicting the molecular mechanisms of USP9X-HIF-2ɑ proteostatic regulation in priming the HGSOC stemness downstream of hypoxic and TGF-β signaling pathway. USP9X, as an important downstream effector, mediates TGF-β-induced stemness and chemoresistance of HGSOC via stabilizing HIF-2ɑ. Meanwhile, the hypoxia tumor microenvironment activates the TGF-β-USP9X-HIF-2α CSCs regulatory axis. WP1130, a USP9X inhibitor, represses tumor formation, and overcomes paclitaxel or platinum resistance. Antagonizing USP9X maybe a promising strategy to conquer clinical chemoresistance problem in HGSOC, even other cancers.

The above results based on HGSOC cell line, patient-derived organoids and PDX model all indicate that USP9X inhibitor can significantly improve bulk cancer cells chemosensitivity. Then we were curious whether the inhibitor could meliorate the response of chemoresistant cancer cells to chemotherapy drugs and offer a new adjuvant treatment strategy to clinical chemo-resistant patient. We took advantage of CDDP-resistant and PTX-resistant cell lines and compared the treatment efficacy of WP1130 or CDDP or PTX alone or in combination. Similar as USP9X knockdown in the same cell lines, the IC50 values, TGF-β signaling activities, EMT and CSCs markers, as well as HIF-2α expression were all significantly reduced by WP1130, even more effective in combination with CDDP or PTX (Fig. 7G and Supplementary Fig. S7E). More excitingly, increased numbers of apoptotic CDDP-resistant and PTX-resistant cells were observed in the combined treatments (Fig. 7G). These results highlight that inhibiting USP9X may be a promising therapeutic strategy to clinical chemoresistant HGSOC patients through disrupting the TGF-β and hypoxia-induced CSCs behaviors (Fig. 8).

## Discussion

TGF-β signaling has been recognized to be the key trigger for CSCs-driven EMT and chemoresistance through various cancers [9–12, 16–19]. However, how is TGF-β signaling activated in tumor microenvironment? How could we target TGF-β signaling more effectively and precisely? These challenging issues have hindered the translational application of TGF-β antagonists into clinic. Herein, we define the hypoxic tumor microenvironment as the chief stimulator of autocrined TGF-β signaling, which further promotes CSCs occurrence through USP9X-HIF-2α proteostatic regulation. Furthermore, by using multiple clinical models, especially therapies on patients-derived organoids or xenografts, we certify that antagonizing USP9X efficiently represses tumor formation, metastasis, CSCs occurrence, while increases chemosensitivity. The current study demonstrates the key oncogenic role of USP9X that coordinates hypoxia and TGF-β signaling in promoting HGSOC CSCs function, and provides a promising strategy to meliorate tumor relapse, metastasis, chemoresistance, and other tough clinical problems of HGSOC.

Post-translational modifications on core signaling components, especially reversible ubiquitylation of type I TGF-β receptor (TGFβR1) kinases and Smads, play critical roles in determining the outcome of TGF-β signaling. USP9X participates in TGF-β signaling by deubiquitylating Smad4 and TGFβR2, or by inducing TGF-β2 transcription, as shown in granulosa cells apoptosis and lung cancer radioresistance [22, 24, 42]. On the other hand, TGF-β signaling elevates the deubiquitinase activity of USP9X by phosphorylation in neurodevelopment and breast cancer metastasis [23, 25]. In the current study, we notice that there is an internal positive regulation loop between USP9X and TGF-β signaling. Not only is USP9X subjected to transcription-dependent upregulation by TGF-β-Smad2/3, but also phosphorylated Smad2/3 level is reduced upon USP9X deprival. Therefore, the mutual positive regulatory relationship between USP9X and TGF-β signaling occurs through various physiological and pathological processes, which makes USP9X an appropriate target of TGF-β signaling in HGSOC or other cancers.

USP9X, as a DUB, functions in promoting stemness of CSCs and contributes to the radioresistance/chemoresistance of several types of cancers, mainly by deubiquitylating different substrates. In glioblastoma, USP9X maintains mesenchymal identity of CSCs and promotes ionizing radiation or temozolomide resistance through deubiquitylating ALDH1A3 [31]. In breast cancer, aggressive B-cell lymphoma and glioblastoma, USP9X alleviates tumor cell survival and confers chemoresistance through YAP1, XIAP or Mcl-1 stabilization [43–45]. Here, we found that USP9X promotes CSCs-driven chemoresistance by deubiquitylating HIF-2α in HGSOC. Our findings thus solidify the oncogenic role of USP9X in terms of cell fate determination, and reinforce the function mechanism in mediating TGF-β signaling within CSCs-driven chemoresistance.

Besides HIF-2α, USP9X has been reported to inhibit degradation of the other key hypoxia factor, HIF-1α, in breast cancer [35]. Whereas, in our study, the protein level of HIF-1α was not significantly affected by USP9X in HGSOC. There are two possible reasons for this discrepancy. One reason could be that the substrates of USP9X may vary in different contexts. On the other hand, the varied N-terminal transactivation domains confer target gene specificity as well as interactome difference between HIF-2α and HIF-1α [46]. Under hypoxic tumor microenvironment, HIF-1α is more likely to regulate the expression of glycolysis and cell survival genes. While HIF-2α is apt to be involved in maintaining stem cell pluripotency and promoting tumor growth, more dramatic than HIF-1α [46–48]. Recently, Zhang et al identified USP33 to be the principle deubiquitinase induced by hypoxic condition and responsible for HIF-2α protein stability regulation within GSCs development [36]. While in the situation of HGSOC, it seems the USP9X-HIF-2α axis plays dominant role and mediates the function of hypoxia signaling in the CSCs maintenance and chemoresistance.

Accumulating evidences have shown that selective antagonism of DUBs could be a promising treatment strategy for cancer therapy or resolving clinical chemoresistance problems, thus a number of DUB inhibitors are actually under preclinical studies and clinical trials [49, 50]. For instance, USP7 inhibitor USP7i/compound 41 re-sensitize chemoresistant MYCN-overexpressing PDX models to chemotherapy in vivo [51]. USP8 inhibitor aids in overcoming hepatocellular carcinoma resistance via suppressing receptor tyrosine kinases [52]. In the current study, antagonizing USP9X by inhibitor WP1130 efficiently represses tumor formation, CSCs content, TGF-β signaling thus increases chemosensitivity through organoids, PDX, and chemoresistant cell models. The similar efficiency of WP1130 was also showed in glioblastoma which resolve the ALDH1A3+ CSCs-driven chemoresistance problem [31]. In breast cancer, WP1130 can also enhance cellular sensitivity to cisplatin and paclitaxel by downregulating endogenous Snail1 protein level [34]. Therefore, adjuvant application of DUBs inhibitors shed some light on conquering the clinical challenge of chemoresistance. Certainly, more specific inhibitors, such as those designed against protein protein interaction inhibitors (PPI) of DUBs and related substrates might give more precise and specific efficiency.

In conclusion, we found that hypoxic tumor microenvironment is the chief culprit to induce CSCs-driven chemoresistance by activating TGF-β signaling in HGSOC. USP9X is the critical downstream effector that converges hypoxia and TGF-β signaling function in CSCs maintenance and chemoresistance (Fig. 8). Antagonizing USP9X maybe a promising strategy to conquer clinical chemoresistance problem in HGSOC even other malignancies.

## Supplementary information

Supplementary information

Supplementary figure 1

Supplementary figure 1

Supplementary figure 2

Supplementary figure 3

Supplementary figure 4

Supplementary figure 5

Supplementary figure 6

Supplementary figure 7

Supplementary Movie S1

Supplementary Movie S2

Supplementary Movie S3

Supplementary Movie S4

Supplementary Movie S5

Supplementary Movie S6

Supplementary Movie S7

Supplementary Movie S8

Supplementary Movie S9

Supplementary Movie S10

Uncropped Blots

## Data Availability

All data are available in the main text or the supplementary materials. Raw mass spectrometry proteomics data have been deposited at ProteomeXchange Consortium via the iProX partner repository with the dataset identifier PXD050476. The RNA-seq data generated in this study has been deposited in the Gene Expression Omnibus (GEO) under the accession GSE286397 available from GEO datasets. GEO Accession Number: GSE241221, GSE51373, GSE131978, GSE25191, GSE235329 and TCGA-OV dataset (https://portal.gdc.cancer.gov/projects/TCGA-OV) were used for analyses in this study. All datasets will be publicly available upon publication.
