# Supplementary material for: USP9X integrates TGF-β and hypoxia signalings to promote ovarian cancer chemoresistance via HIF-2α-maintained stemness
Source: Cell Death Dis. 2025 Apr 18;16(1):312. doi: 10.1038/s41419-025-07646-5 (PMC12006517; doi:10.1038/s41419-025-07646-5)
Supplement: Supplementary file 1 — Supplementary information [file 41419_2025_7646_MOESM1_ESM.docx]

**Supplementary Data for**

**USP9X integrates TGF-β and** **hypoxia signalings to promote ovarian cancer chemoresistance via HIF-2α-maintained stemness**

**Authors:** Zhenlei Zhang^1, *^, Xiujie Yu^2, *^, Liqi Wen^1^, Jia’nan Wang^1^, Zhufeng Li^3^, Yu Zhang^1^, Jiayu Cheng^1^, Ronglin Kan^1^, Wanting Zhang^1^, Yan Shen^2, #^, Shukai Yuan^1, #^, Li Zhao^1, #^

**This PDF file includes:**

Legends for Figures S1 to S7

Legends for Movies S1 to S10

Table S1

# Fig. S1. USP9X correlates with TGF-β signaling function in mediating HGOSC development and chemoresistance.

(**A**) Survival analyses in HGSOC patients were performed based on high or low expressions of key TGF-β signaling factors, TGFB1, TGFBR1, SAMD2, and SMAD3, in TCGA and GEO databases. (**B**) The pathological information of patients with clinical ovarian tumors (HGSOC tissues (n = 83), including benign (n = 17), borderline (n = 25), and malignant tumors (n = 41)), were statistically analyzed, and the correlation between the expression of p-Smad3 or USP9X and the age, tissue classification, malignancy, migration and ascites formation of patients was analyzed based on **Fig. 1A**. Correlation analysis was performed by Pearson's chi squared test (χ^2^-test). (**C**) IB analysis of p-Smad3, Smad2/3 and USP9X in ID8, CAOV3 or SKOV3 cells after short treatment (24 h) of CDDP (5 μM or 10 μM), CBP (5 μM or 10 μM) or PTX (2.5 nM or 5 nM). (**D**) Dose-response curve and IC50 of ID8 cells, CDDP-resistant (ID8-rCDDP) or PTX-resistant (ID8-rPTX) ID8 cells in response to CDDP or PTX for 72 h. (**E**) mRNA expressions of CSCs (OCT4, NANOG, CD133, and EPAS1) and drug resistance genes (ABCB1, ABCG2, and ABCC1) were detected by qRT-PCR in ID8 cells, ID8-rCDDP or ID8-rPTX cells. (**F**) Expression of USP9X were plotted between Grade low (Grade 1 and 2) and high (Grade 3 and 4) group based on HGSOC from TCGA. (**G**) Survival analyses in PTX or CDDP treatment HGSOC patients were performed based on high or low expressions of USP9X in TCGA and GEO databases. (**H**) Correlation analysis between USP9X and TGF-β signature in OC based on TCGA. (**I**) GSEA analysis of TGF-β signaling pathway based on GSE25191. **(J)** IB analysis of p-Smad3 and USP9X in CSCs spheroids formed from fresh HGOSC samples. (**K**) Spheroid formation number of freshly HGOSC samples. Spheroids larger than 50 μm in diameter were used for analysis. (**L**) mRNA expressions of CSCs genes (ALDH1A1, CD133, CD44, OCT4, and NANOG) were detected by qRT-PCR in CSCs spheroids from (**K**). (**M**) Correlation analysis between Spheroid formation number and CSCs maakers based on (**L** and **K**). (**N-Q**) p-Smad3 and USP9X protein and mRNA expression was detected by IB (**N** and **P**) or qRT-PCR (**O** and **Q**) in ID8, CAOV3 or SKOV3 cells treated by TGF-β1 (5 ng/mL) or TGF-β inhibitor (SB431542) (5 µM) at different times. (**R**) USP9X mRNA expression was detected by qRT-PCR in CAOV3 or SKOV3 cells transfected by Smad2 or Smad3 overexpression plasmid for 48 h. (**S** and **T**) Dual luciferase assays were performed to analyze the transcriptional activation of USP9X promoter with different doses of Smad2 or Smad3 (**S**) and USP9X-promoter-wild-type and mutant (USP9X-promoter-M, -1121bp site was mutated from CCCCAGACGG to CCCTTTACGG.) by Smad2 (**T**) in HEK293T cells. Data are shown as the mean ± s.d (**D, E, F, K, L, M, O, Q-T**). P values were calculated by unpaired two-tailed Student’s t test (**F, E, O, Q-T**). Correlation analysis was performed by Pearson correlation, P values (two-tailed) were calculated by Pearson r (**H, M**). n = 3 biological independent samples (**C-E**, **J**, **N**-**T**).

# Fig. S2. USP9X mediates CSCs-dependent OC metastasis and chemoresistance.

(**A** and **B**) USP9X knocked-down CAOV3 or ID8 cells treated with or without TGF-β1 (5 ng/mL) were subjected to cell migration assays of wound-healing (**A**) or transwell (**B**). Scale bars, 50 µm. The right panels were the quantization chart. (**C**) Stemness genes mRNA expression was detected by qRT-PCR in USP9X knocked-down CAOV3 or ID8 cells treated with or without TGF-β1 (5 ng/mL) from **Fig. 2B**. (**D**) The quantization chart of ALDH1A1^+^/CD44^+^ cells from **Fig. 2C**. (**E**) Metastasis to the peritoneum and intestines in USP9X knocked-down or control luciferase-expressing ID8 cells (ID8-luc) transplanted mice. n = 4 mice per group. The right panels were the quantization chart. (**F**) IB analysis of USP9X and p-Smad3 in USP9X knocked-down ID8-rPTX or ID8-rCDDP cells. (**G**) USP9X and stemness genes mRNA expression was detected by qRT-PCR in USP9X knocked-down ID8-rPTX or ID8-rCDDP cells. (**H** and **I**) Representative images of p-Smad3 and USP9X immunofluorescence (IF) on USP9X knocked-down ID8-rPTX or ID8-rCDDP cells (**H**). Scale bars, 50 µm. Positive cell statistics in **(I)**. Data are shown as the mean ± s.d (**A**-**E**, **G**, **I**). P values were calculated by unpaired two-tailed Student’s t test (**A**-**E**, **G**, **I**). n = 3 biological independent samples (**A-D, G-I**).

# Fig. S3. Hif-2α is a potent interaction partner of USP9X.

(**A**) The diagram showed the possible interacting proteins of USP9X or HIF-2α from affinity purification and mass spectrometry (AP-MS) analysis in HEK293T cells. (**B**) IP were performed to examine the interaction between over-expressed Flag-USP9X and endogenous HIF-2α in HEK293T cells. (**C** and **D**) Immunoprecipitations (IP) were performed to validate endogenous binding of USP9X and HIF-2α in SKOV3 cells. (**E**) IF was performed to validate colocalization of USP9X and HIF-2α in CAOV3 and ID8 cells. Scale bars, 20 µm. The right panels showed fluorescence intensities. n = 3 biological independent samples (**B**-**E**).

# Fig. S4. USP9X maintains Hif-2α stability by deubiquitylating.

(**A**) HIF-2α expression was detected after over-expression of different doses of Flag-tagged USP9X (1 µg, 2 µg or 3 µg) in HEK293T cells. (**B**) HIF-2α expression was examined in CAOV3 or ID8 cells with or without WP1130 (4 μM, 8 μM or 12 μM), 20 μM MG132 treatment for 12 hours. (**C** and **D**) Half-lives of HIF-2α were analyzed after over-expression of USP9X^WT^ or USP9X^C1566S^ in HEK293T and ID8 cells treated with Cycloheximide (CHX, 100 μg/ml) for indicated time (0 h, 4 h, 8 h or 12 h) before harvesting. (**E**) Half-lives of HIF-2α were analyzed after USP9X knocked-down in ID8 cells treated with CHX (100 μg/ml) for indicated time (0 h, 4 h, 8 h or 12 h) before harvesting. (**F**) HIF-2α expression was examined in CAOV3 or ID8 cells with or without WP1130 (4 μM), 100 µg/ml (CHX) treatment for 0 h, 4 h, 8 h or 12 h. (**G**) The pathological information of patients with clinical ovarian tumors (HGSOC tissues (n = 83), including benign (n = 17), borderline (n = 25), and malignant tumors (n = 41)), were statistically analyzed, and the correlation between the expression of HIF-2α and the age, tissue classification, malignancy, migration and ascites formation of patients was analyzed based on **Fig. 4K**. Correlation analysis was performed by Pearson's chi squared test (χ^2^-test). (**H**) IB analysis of HIF-2α in freshly collected tumor tissues in serous ovarian cancer with different progression, including benign (n = 9), borderline (n = 9), and malignant tumor (n = 9), the same cohort as in **Figure 1A**, and the expression of HIF-2α was counted, statistically analysis was performed by χ^2^-test. n = 3 biological independent samples (**A-F**).

# Fig. S5. Hif-2α mediates USP9X-promoted CSCs function downstream of TGF-β.

(**A** and **B**) HIF-2α knocked-down CAOV3 or ID8 cells treated with or without TGF-β1 (5 ng/mL) were subjected to cell migration assays of wound-healing (**A**) or transwell (**B**). Scale bars, 50 µm. The right panels were the quantization chart. (**C** and **D**) USP9X knocked-down and HIF-2α overexpressed CAOV3 or ID8 cells treated with or without TGF-β1 (5 ng/mL) were subjected to cell migration assays of wound-healing **(C)** or transwell (**D**). Scale bars, 50 µm. The right panels were the quantization chart. (**E**) Stemness genes mRNA expression was detected by qRT-PCR in USP9X knocked-down and HIF-2α overexpressed CAOV3 or ID8 cells treated with or without TGF-β1 (5 ng/mL) for 2 weeks from **Fig. 5B**. (**F**) IB analysis of HIF-2α in stem cell spheroids formed from fresh HGOSC samples (n = 32), the same cohort as in **Fig. 1F**. (**G**) Survival analyses in all types of ovarian cancer or serous ovarian cancer of OC patients were performed based on high or low expressions of HIF-2α, in TCGA and GEO databases. (**H**) Representative images of HIF-2α immunofluorescence (IF) on stainings on ID8, ID8-rPTX, ID8-rCDDP, USP9X knocked-down ID8-rPTX or ID8-rCDDP cells. Scale bars, 50 µm. The right panels were the quantization chart. Data are shown as the mean ± s.d (**A-E**, **H**). P values were calculated by unpaired two-tailed Student’s t test (**A-E**, **H**). n = 3 biological independent samples (**A**-**E**, **H**).

# Fig. S6. TGF-β and hypoxia signalings converge on USP9X stabilized Hif-2α.

(**A**) USP9X, HIF-2α, and HIF-1α protein and mRNA expression was detected by IB (left panel) or qRT-PCR (right panel) in CAOV3, ID8, and SKOV3 cells incubated under 1% O_2_ for different time (0 h, 12 h, 24 h or 36 h). (**B**) Hypoxia-related elements (HRE) luciferase assays were performed to analyze the activity of hypoxic signaling pathways in USP9X knocked-down CAOV3 and ID8 cell incubated under 1% O_2_ for 24 h. (**C**) USP9X and HIF-2α protein and mRNA expression was detected by IB (left panel) or qRT-PCR (right panel) in SKOV3 cells treated with TGF-β1 (5 ng/mL) and incubated under 1% O_2_ for 24 h. (**D**) TGF-β1 mRNA expression was detected by qRT-PCR in CAOV3 and ID8 cells incubated under 1% O_2_ for different time. (**E**) HIF-1α and HIF-2α binding motif, and potential HRE on TGF-β1 promoter based on Jasper database. (**F**) Dual luciferase assays were performed to analyze the transcriptional activation of TGF-β1-promoter-wild-type and mutant (TGF-β1-promoter-M, -967bp site was mutated from CCACGTGGG to CAAAATGGG.) by Hypoxia (incubated under 1% O_2_), HIF-1α, HIF-2α, or HIF-1α and HIF-2α. (**G**) IB analysis of TGF-β1 in USP9X knocked-down CAOV3 or ID8 cells incubated under 1% O_2_ for 24 h. (**H**) SRE luciferase assays were performed to analyze the activity of hypoxic signaling pathways in USP9X knocked-down CAOV3 and ID8 cell incubated under 1% O_2_ for 24 h. (**I**) Bar plots of normalized enrichment scores (NES) from GSEA for the differentially expressed genes (DEGs) between epithelial cells of post-and pre-chemotherapy patients based on GSE235329 (NES > 1.5, P < 0.05). (**J**) GSEA of hypoxia and TGF-β signaling pathway in post-or pre-chemotherapy patients based on GSE235329 and GSE241221 (scRNA-sequencing). Data are shown as the mean ± s.d (**A-D, F, H**). P values were calculated by unpaired two-tailed Student’s t test (**A-D, F, H**). n = 3 biological independent samples (**A-D, F, H**).

# Fig. S7. USP9X inhibitor treatment sensitizes HGSOC to chemotherapy.

(**A**) Dose-response curve and IC50 values of CAOV3 and ID8 cells treated with different concentrations of WP1130 for 72 h, respectively. (**B**) CSCs and EMT markers expression was detected by qRT-PCR in USP9X knocked-down CAOV3 and ID8 cells treated with WP1130 (1 µM), CDDP (1 µM) or PTX (1 nM) for 24 h. (**C**) Representative images of USP9X and HIF-2α whole-mount IF stainings on primary HGSOC cells derived organoids, treated with WP1130 (1 µM), CBP (1 µM) or PTX (1 nM) for 72 h. Organoids larger than 75 μm in diameter were used for analysis. The right panels were the quantization chart. Scale bars, 100 µm. (**D**) Representative images of HIF-2α and USP9X IHC stainings on tumors of (**Fig. 7D**). The right panels were the quantization chart. Scale bars, 50 µm. (**E**) Dose-response curve and IC50 values of ID8-rPTX or ID8-rCDDP cells treated with WP1130 (1 µM) and different concentrations of PTX or CDDP for 72 h, respectively. (**F**) CSCs (OCT4, NANOG, and CD133), drug resistance (ABCB1, ABCG2, and ABCC1) and EMT markers expression was detected by qRT-PCR in ID8-rPTX and ID8-rCDDP cells, treated with WP1130 (1 µM), CDDP (1 µM) or PTX (1 nM) for 24 h. Data are shown as the mean ± s.d (**A-F**). P values were calculated by unpaired two-tailed Student’s t test (**A-F**). n = 3 biological independent samples (**A-F**).

# Movies S1 to S10. PLA was performed to validate colocalization of USP9X and HIF-2α.

**Movie S1.** Representative movie showing 3D image of Duolink negative control in CAOV3 cells, related to **Fig. 3F**.mp4

**Movie S2.** Representative movie showing 3D image of Duolink USP9X in CAOV3 cells, related to **Fig. 3F**.mp4

**Movie S3.** Representative movie showing 3D image of Duolink HIF-2α in CAOV3 cells, related to **Fig. 3F**.mp4

**Movie S4.** Representative movie showing 3D image of Duolink USP9X-HIF-2α in CAOV3 cells, related to **Fig. 3F**.mp4

**Movie S5.** Representative movie showing 3D image of Duolink USP9X-HIF-2α in CAOV3 cells treated with TGF-β1 (5 ng/mL) for 24 h, related to **Fig. 3F**.mp4

**Movie S6.** Representative movie showing 3D image of Duolink negative control in ID8 cells, related to **Fig. 3F**.mp4

**Movie S7.** Representative movie showing 3D image of Duolink USP9X in ID8 cells, related to **Fig. 3F**.mp4

**Movie S8.** Representative movie showing 3D image of Duolink HIF-2α in ID8 cells, related to **Fig. 3F**.mp4

**Movie S9.** Representative movie showing 3D image of Duolink USP9X-HIF-2α in ID8 cells, related to **Fig. 3F**.mp4

**Movie S10.** Representative movie showing 3D image of Duolink USP9X-HIF-2α in ID8 cells treated with TGF-β1 (5 ng/mL) for 24 h, related to **Fig. 3F**.mp4

**Supplementary Table 1.** shRNA, ChIP-qPCR and RT-qPCR primers sequences.

| **shRNA sequences** | |
| --- | --- |
| shUSP9X#12(human) | CGACCCTAAACGTAGACATTA |
| shUSP9X#13(human) | CGATTCTTCAAAGCTGTGAAT |
| shUSP9X#2(mouse) | GATAATTGCAGCCCTTATTAA |
| shUSP9X#3(mouse) | TCGTAATGTATGCCAATTTAG |
| shHIF-2α#2(mouse) | GTATCATGTGTGTCAACTATG |
| shHIF-2α#3(mouse) | GCAGCCCTGAGGACTACTATT |
| **ChIP-qPCR primers** | |
| **Smad2/3-USP9Xpromoter** | |
| SRE1-F | CTGGCAATGGAGGCTAATCTT |
| SRE1-R | GGCCATAGGAGCCCTTCAAA |
| SRE2-F | CCTGACGGGGGCGGAGAACTT |
| SRE2-R | CGGCGGCGGTAGCGGCGACG |
| SRE3-F | AGTCAAACTATGAGCGACGCG |
| SRE3-R | AAGATCTGGCACACCACTCG |
| SRE4-F | TTCCTTATCTAAAATAGAAAA |
| SRE4-R | TAATTATTTCCATGAATCAT |
| **RT-qPCR primers** | |
| **Human** | |
| OCT4-F | CCTGAAGCAGAAGAGGATCACC |
| OCT4-R | AAAGCGGCAGATGGTCGTTTGG |
| NANOG-F | CTCCAACATCCTGAACCTCAGC |
| NANOG-R | CGTCACACCATTGCTATTCTTCG |
| CD133-F | CACTACCAAGGACAAGGCGTTC |
| CD133-R | CAACGCCTCTTTGGTCTCCTTG |
| ALDH1A-F | CGGGAAAAGCAATCTGAAGAGGG |
| ALDH1A-R | GATGCGGCTATACAACACTGGC |
| HIF-2α-F | CTGTGTCTGAGAAGAGTAACTTCC |
| HIF-2α-R | TTGCCATAGGCTGAGGACTCCT |
| ABCB1-F | GCTGTCAAGGAAGCCAATGCCT |
| ABCB1-R | TGCAATGGCGATCCTCTGCTTC |
| ABCG2-F | GTTCTCAGCAGCTCTTCGGCTT |
| ABCG2-R | TCCTCCAGACACACCACGGATA |
| ABCC1-F | CCGTGTACTCCAACGCTGACAT |
| ABCC1-R | ATGCTGTGCGTGACCAAGATCC |
| CD117-F | CGTTCTGCTCCTACTGCTTCG |
| CD117-R | CCCACGCGGACTATTAAGTCT |
| CD44-F | CTGCCGCTTTGCAGGTGTA |
| CD44-R | CATTGTGGGCAAGGTGCTATT |
| SOX2-F | TACAGCATGTCCTACTCGCAG |
| SOX2-R | GAGGAAGAGGTAACCACAGGG |
| **Mouse** | |
| OCT4-F | CAGCAGATCACTCACATCGCCA |
| OCT4-R | GCCTCATACTCTTCTCGTTGGG |
| NANOG-F | GAACGCCTCATCAATGCCTGCA |
| NANOG-R | GAATCAGGGCTGCCTTGAAGAG |
| CD133-F | CTGCGATAGCATCAGACCAAGC |
| CD133-R | CTTTTGACGAGGCTCTCCAGATC |
| ALDH1A1-F | GGAATACCGTGGTTGTCAAGCC |
| ALDH1A1-R | CCAGGGACAATGTTTACCACGC |
| HIF-2α-F | GGACAGCAAGACTTTCCTGAGC |
| HIF-2α-R | GGTAGAACTCATAGGCAGAGCG |
| ABCB1-F | TCCTCACCAAGCGACTCCGATA |
| ABCB1-R | ACTTGAGCAGCATCGTTGGCGA |
| ABCG2-F | CAGTTCTCAGCAGCTCTTCGAC |
| ABCG2-R | TCCTCCAGAGATGCCACGGATA |
| ABCC1-F | CAGTGGTTCAGGGAAGGAGTCA |
| ABCC1-R | CACTGTGGGAAGACGAGTTGCT |
| N-Cadherin-F | CCTCCAGAGTTTACTGCCATGAC |
| N-Cadherin-R | CCACCACTGATTCTGTATGCCG |
| Slug-F | TCTGTGGCAAGGCTTTCTCCAG |
| Slug-R | TGCAGATGTGCCCTCAGGTTTG |
| Snai1-F | TGTCTGCACGACCTGTGGAAAG |
| Snai1-R | CTTCACATCCGAGTGGGTTTGG |
| Vimentin-R | CGGAAAGTGGAATCCTTGCAGG |
| Vimentin-F | AGCAGTGAGGTCAGGCTTGGAA |
| CD117-F | GCCTGACGTGCATTGATCC |
| CD117-R | AGTGGCCTCGGCTTTTTCC |
| CD44-F | TCGATTTGAATGTAACCTGCCG |
| CD44-R | CAGTCCGGGAGATACTGTAGC |
| SOX2-F | GCGGAGTGGAAACTTTTGTCC |
| SOX2-R | GGGAAGCGTGTACTTATCCTTCT |
| SCA1-F | AGGAGGCAGCAGTTATTGTGG |
| SCA1-R | CGTTGACCTTAGTACCCAGGA |
