# Supplementary figures and images for: USP9X integrates TGF-β and hypoxia signalings to promote ovarian cancer chemoresistance via HIF-2α-maintained stemness
Source: Cell Death Dis. 2025 Apr 18;16(1):312. doi: 10.1038/s41419-025-07646-5 (PMC12006517; doi:10.1038/s41419-025-07646-5)

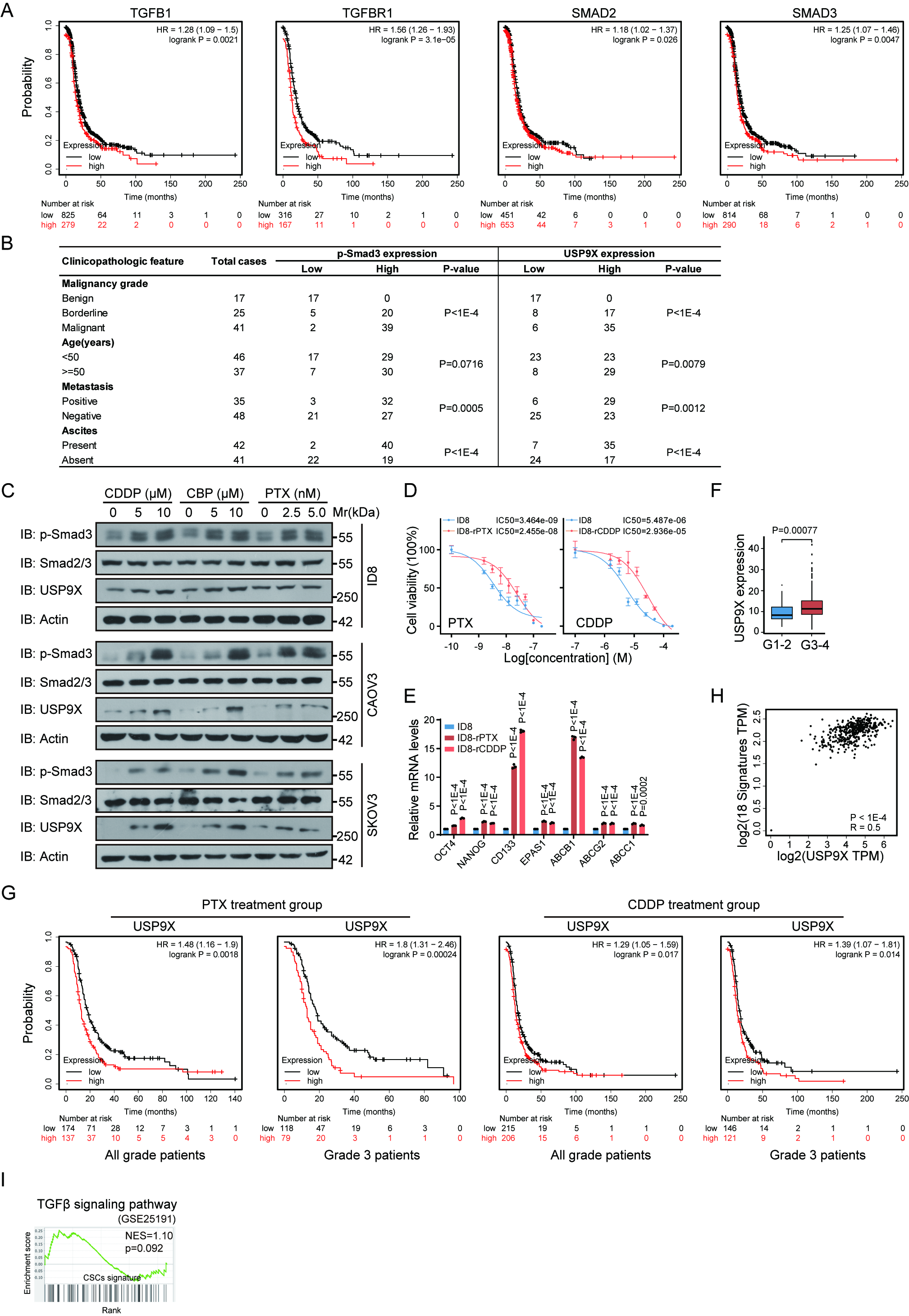

Supplement: Supplementary file 2 — Supplementary figure 1 [file 41419_2025_7646_MOESM2_ESM.tif]

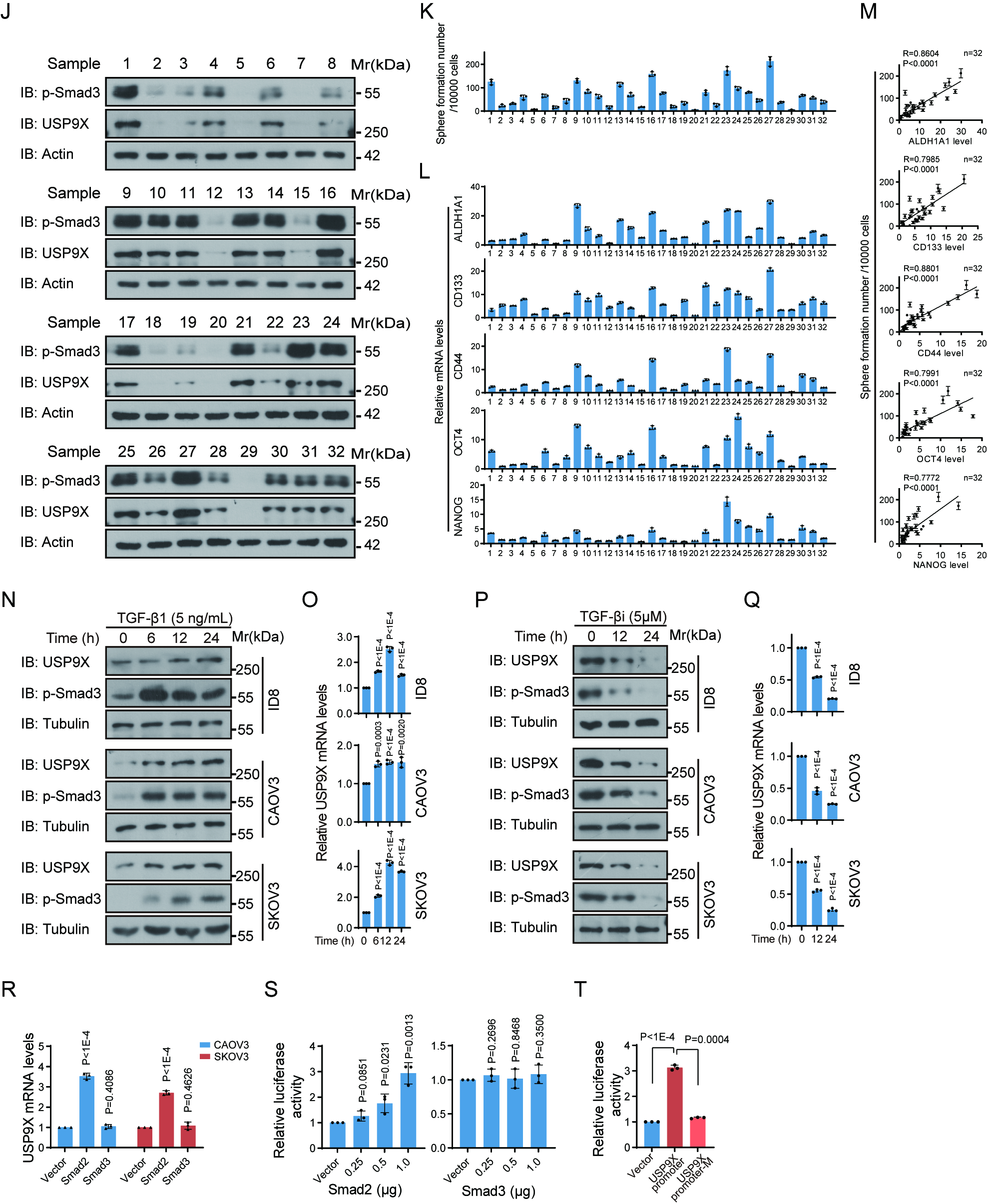

Supplement: Supplementary file 3 — Supplementary figure 1 [file 41419_2025_7646_MOESM3_ESM.tif]

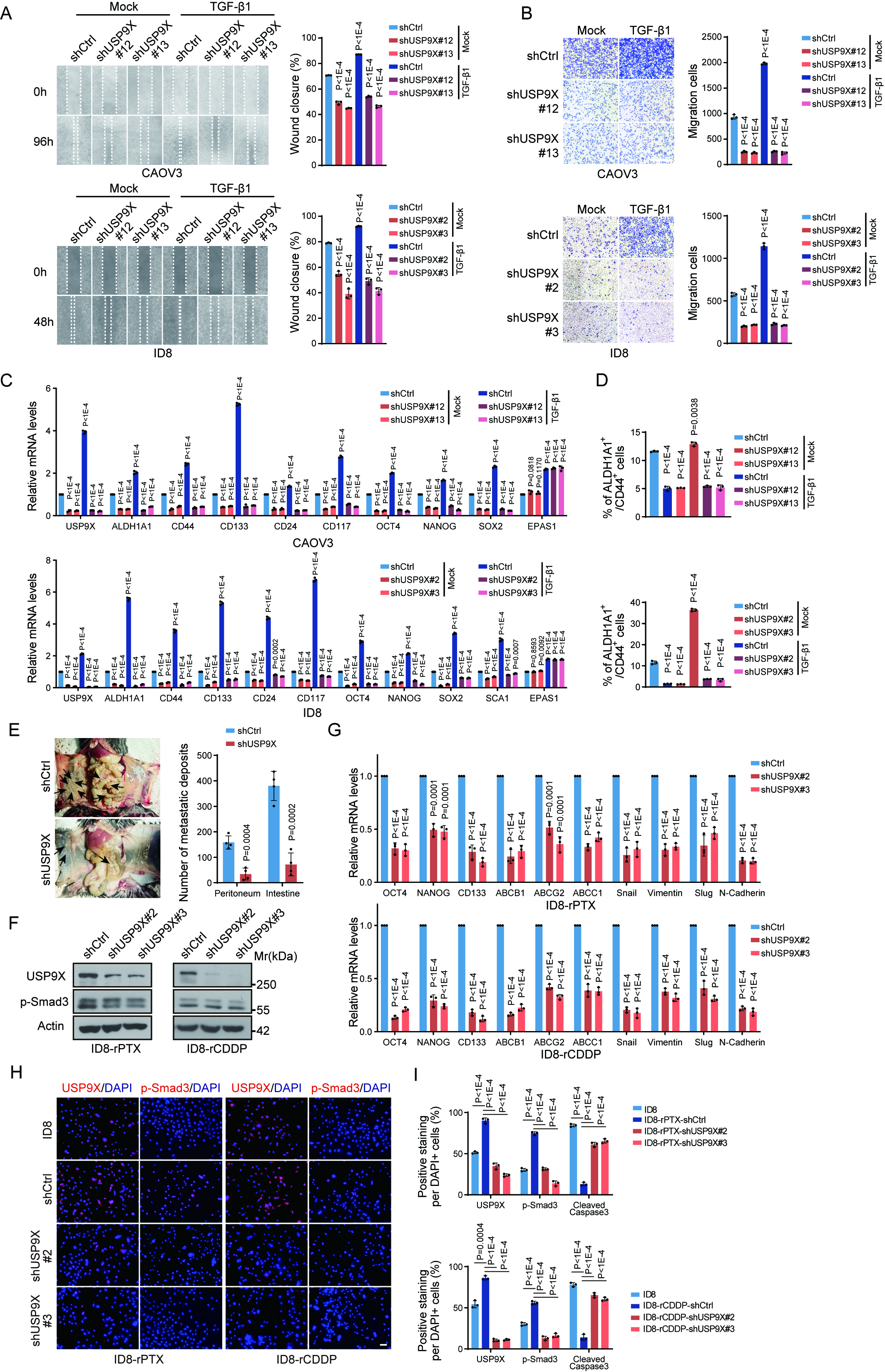

Supplement: Supplementary file 4 — Supplementary figure 2 [file 41419_2025_7646_MOESM4_ESM.tif]

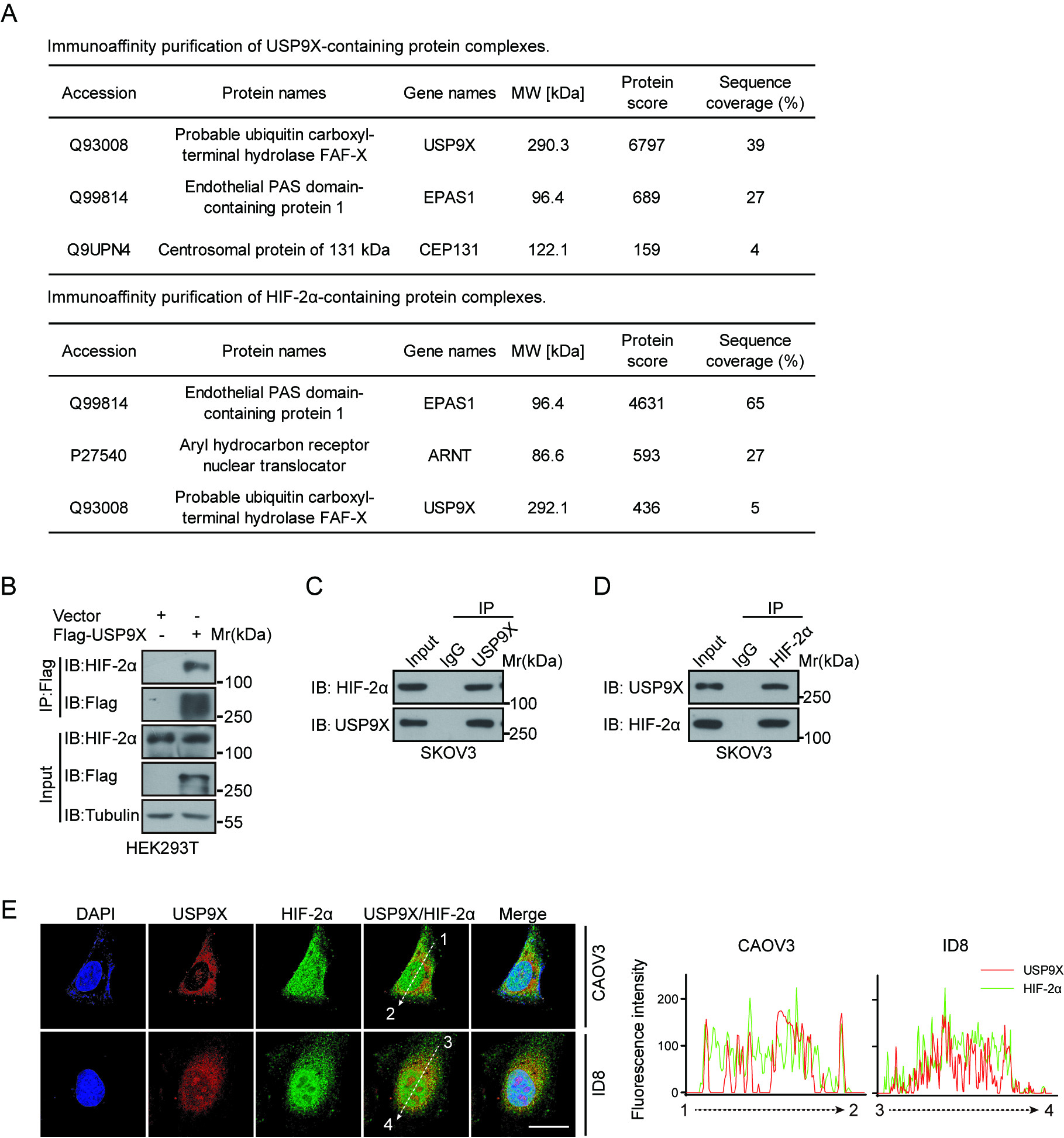

Supplement: Supplementary file 5 — Supplementary figure 3 [file 41419_2025_7646_MOESM5_ESM.tif]

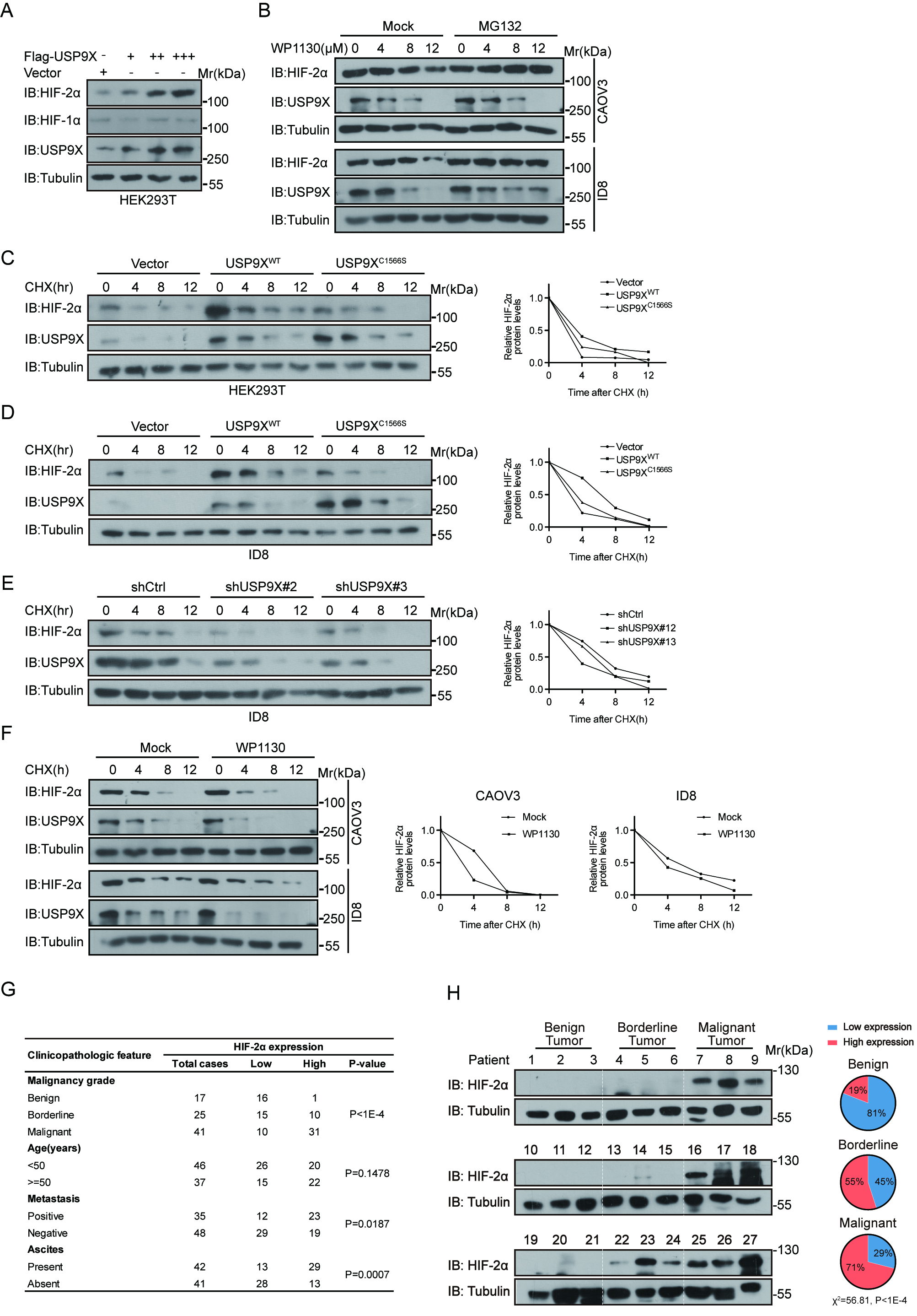

Supplement: Supplementary file 6 — Supplementary figure 4 [file 41419_2025_7646_MOESM6_ESM.tif]

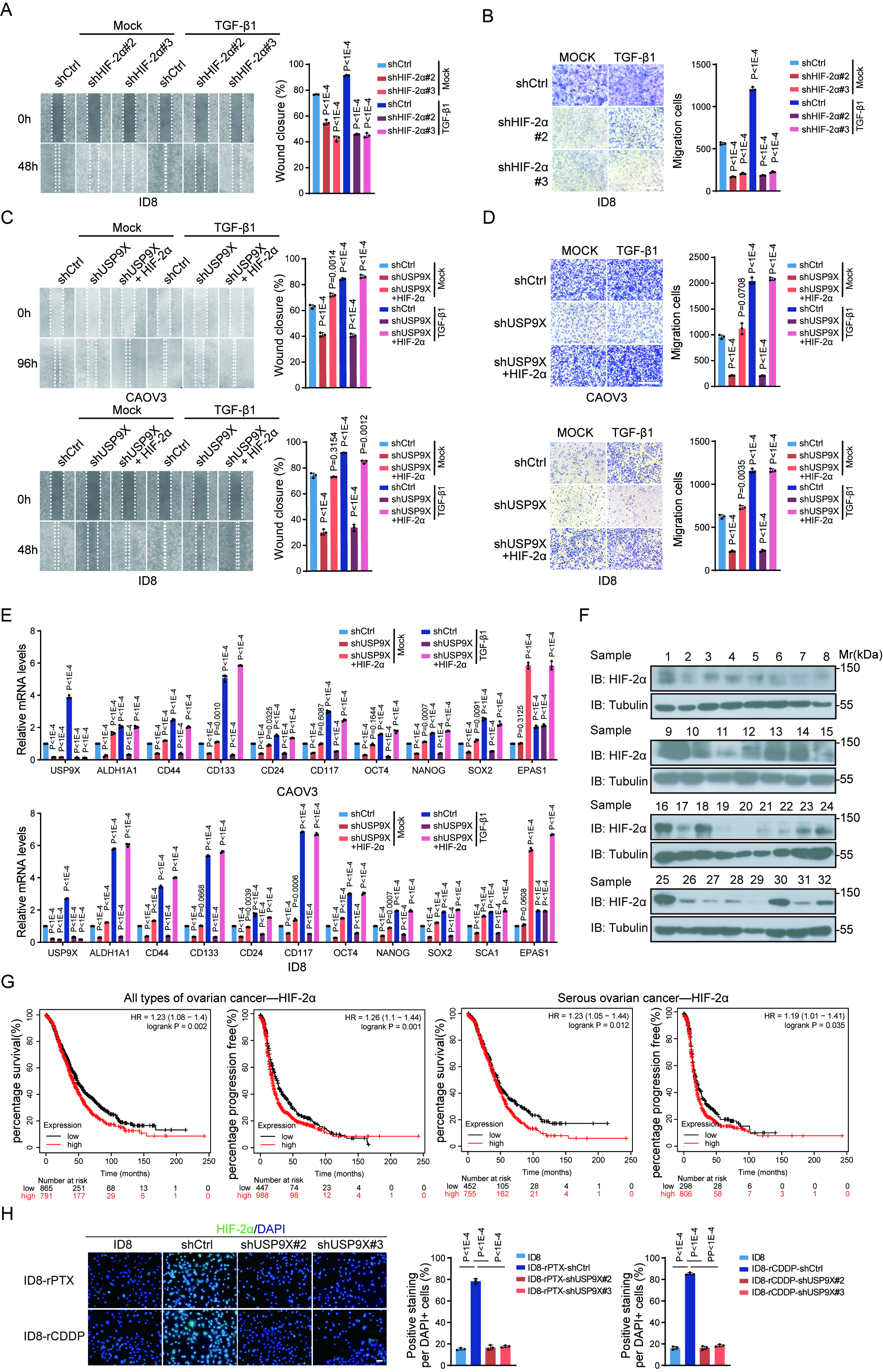

Supplement: Supplementary file 7 — Supplementary figure 5 [file 41419_2025_7646_MOESM7_ESM.tif]

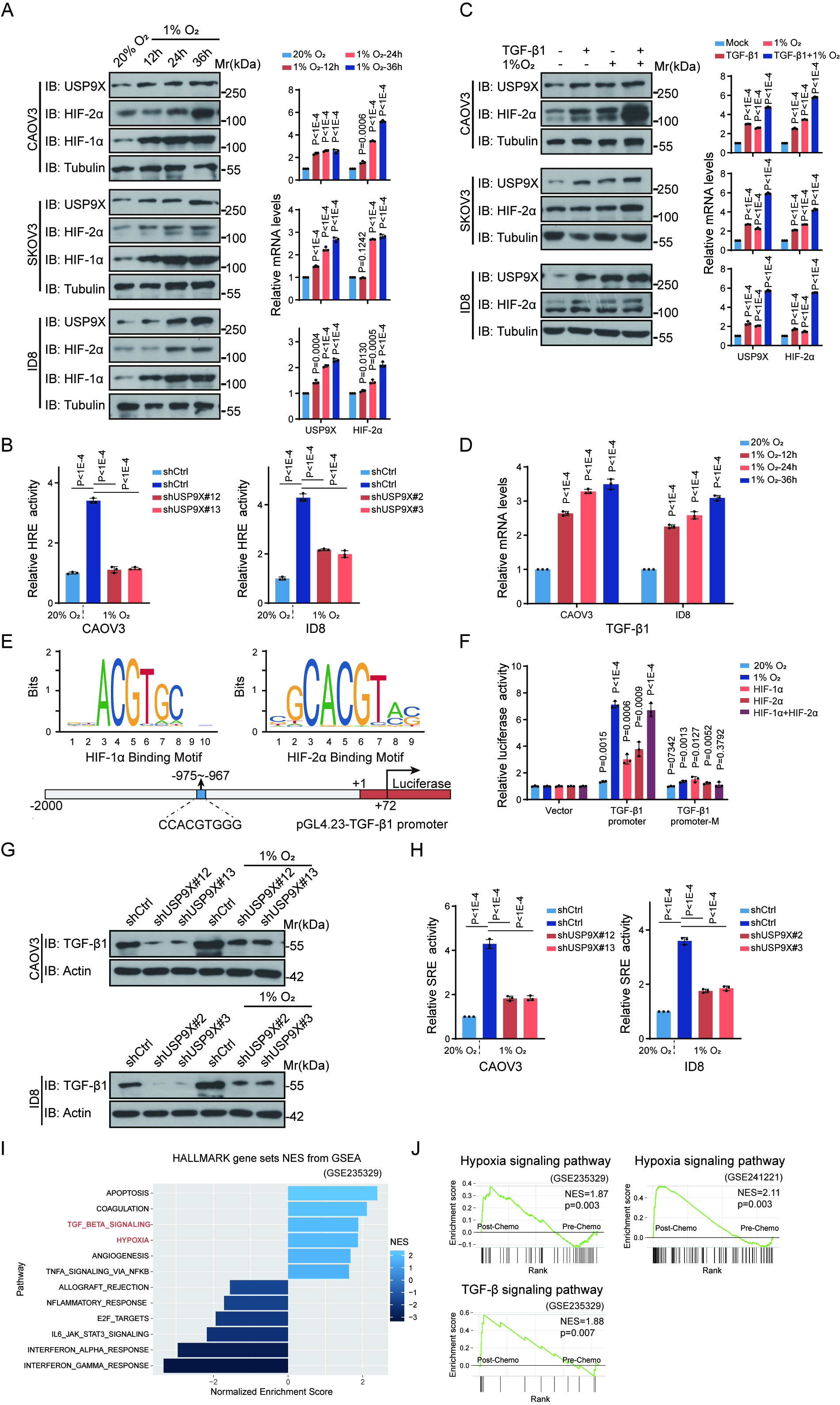

Supplement: Supplementary file 8 — Supplementary figure 6 [file 41419_2025_7646_MOESM8_ESM.tif]

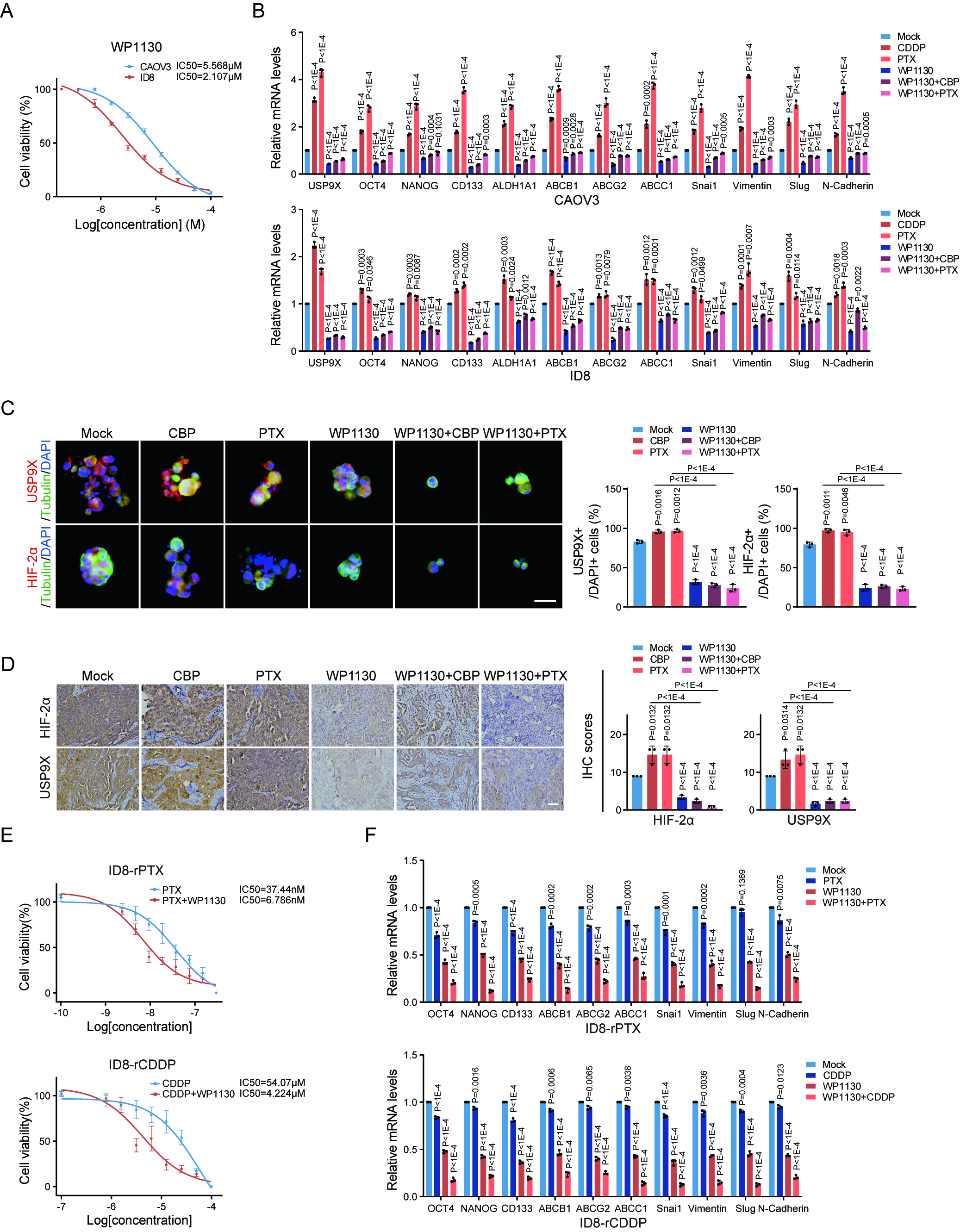

Supplement: Supplementary file 9 — Supplementary figure 7 [file 41419_2025_7646_MOESM9_ESM.tif]
