# Supplementary material for: USP9X integrates TGF-β and hypoxia signalings to promote ovarian cancer chemoresistance via HIF-2α-maintained stemness
Source: Cell Death Dis. 2025 Apr 18;16(1):312. doi: 10.1038/s41419-025-07646-5 (PMC12006517; doi:10.1038/s41419-025-07646-5)

Figure 1B

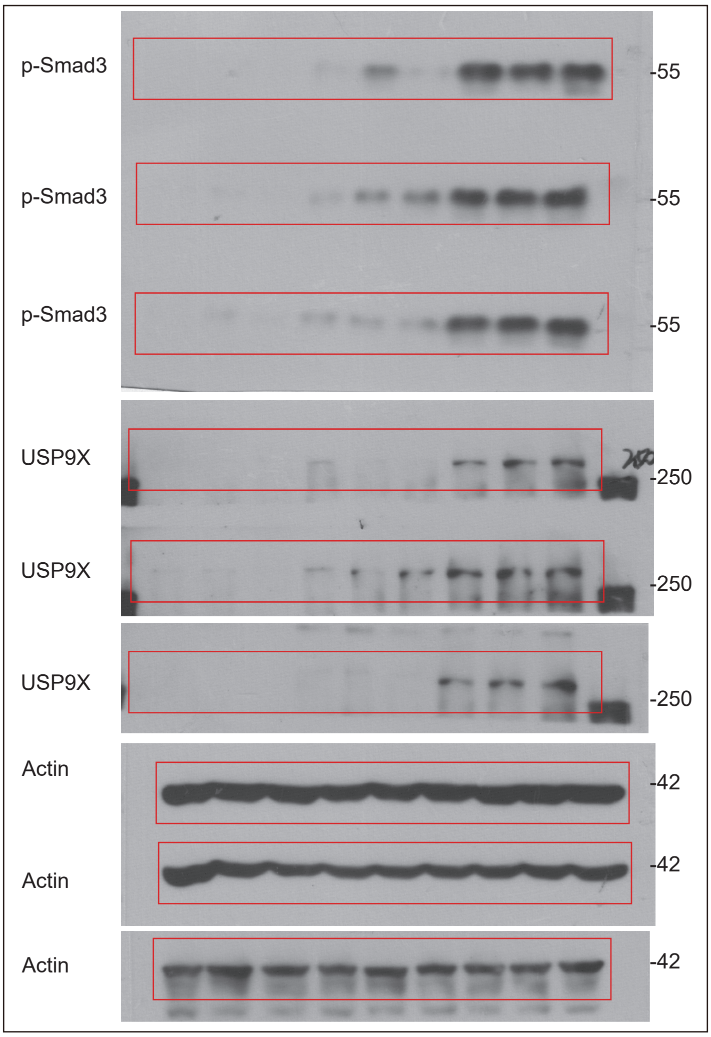

Figure 1D

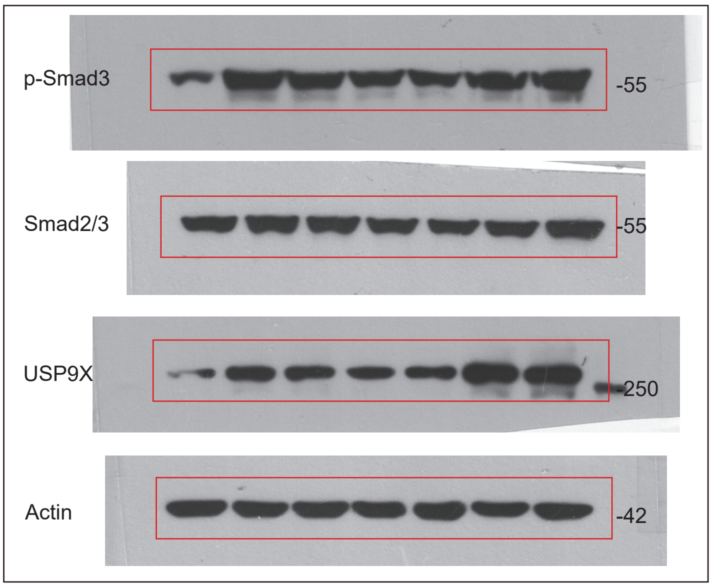

Figure 1M

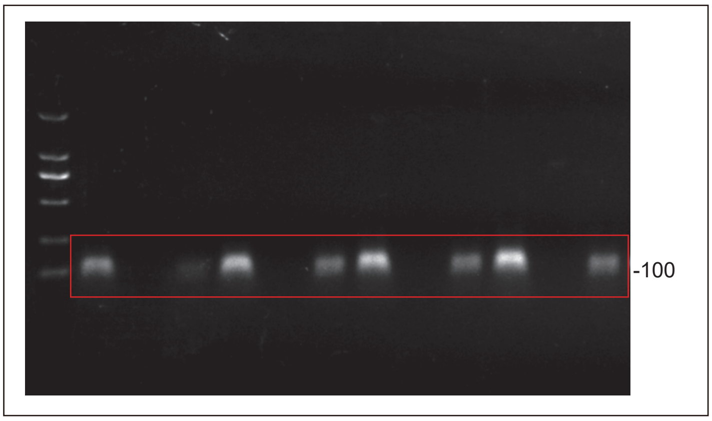

Figure 2A

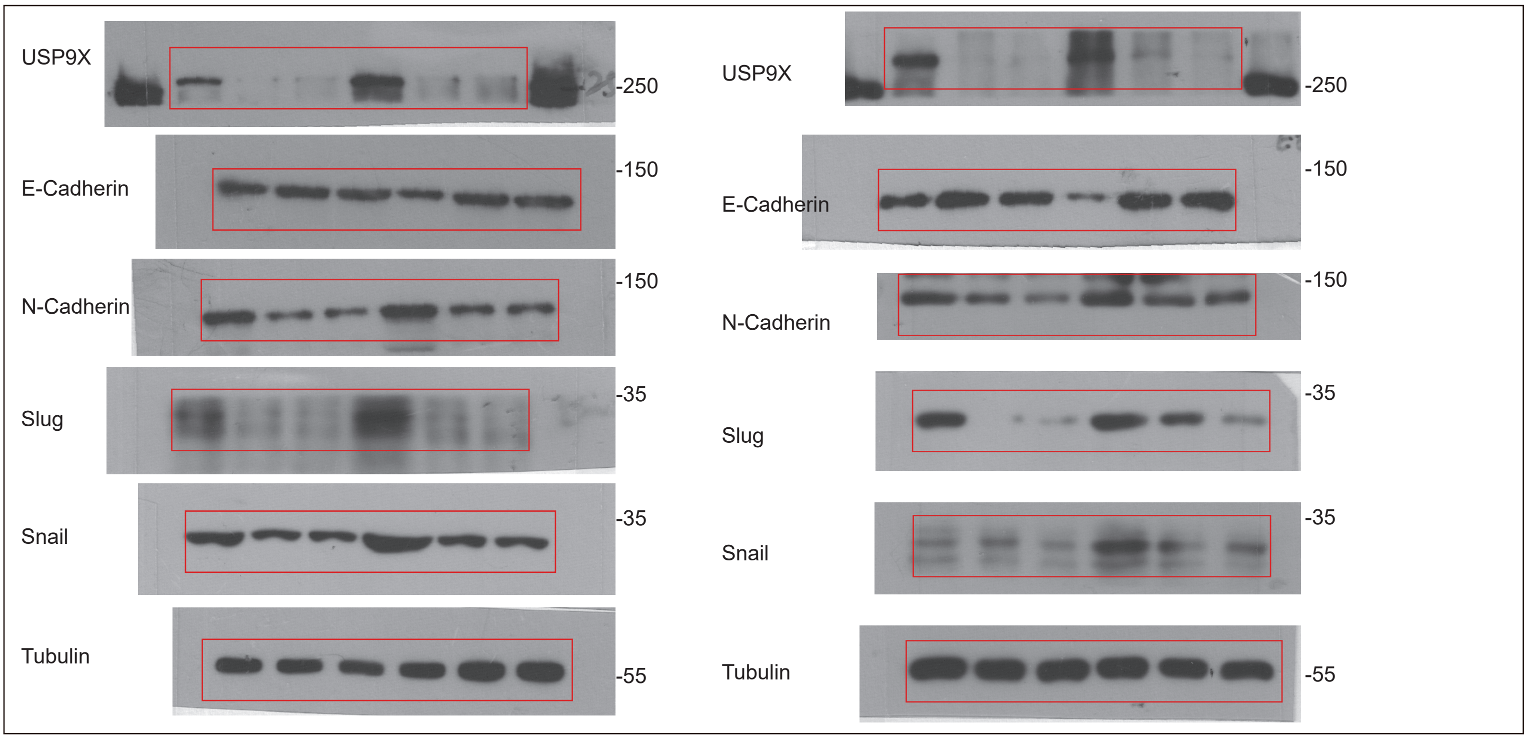
USP9X
-250
E-Cadherin
-150
N-Cadherin
-150
Slug
-35
Snail
-35
Tubulin
-55
USP9X
-250
E-Cadherin
-150
N-Cadherin
-150
Slug
-35
Snail
-35
Tubulin
-55

Figure 3A

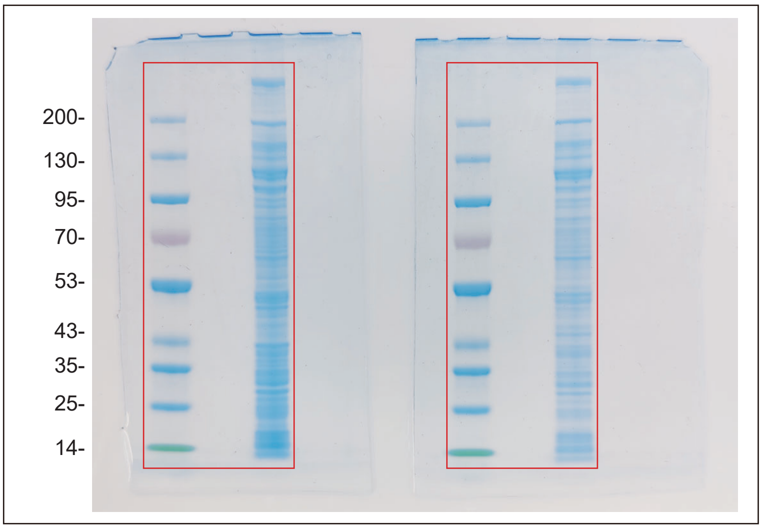

Figure 3B

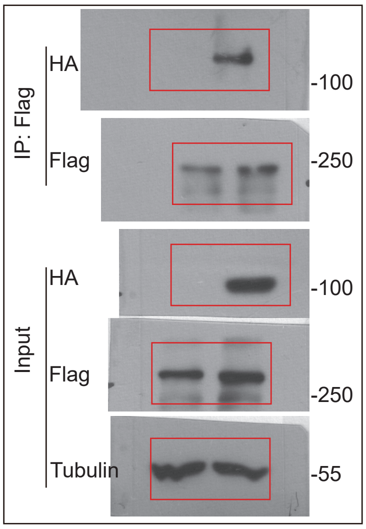

Figure 3C

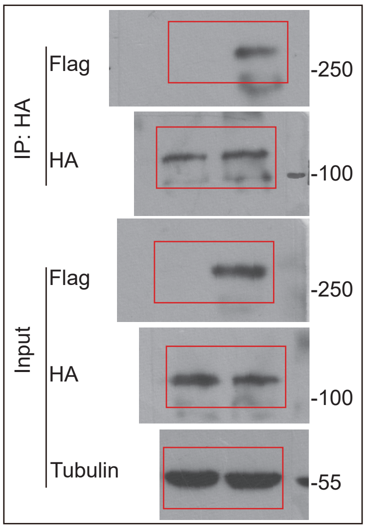

Figure 3D and 3E

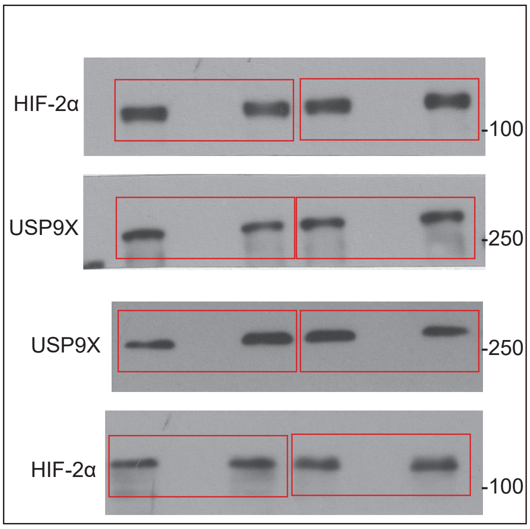

Figure 3G

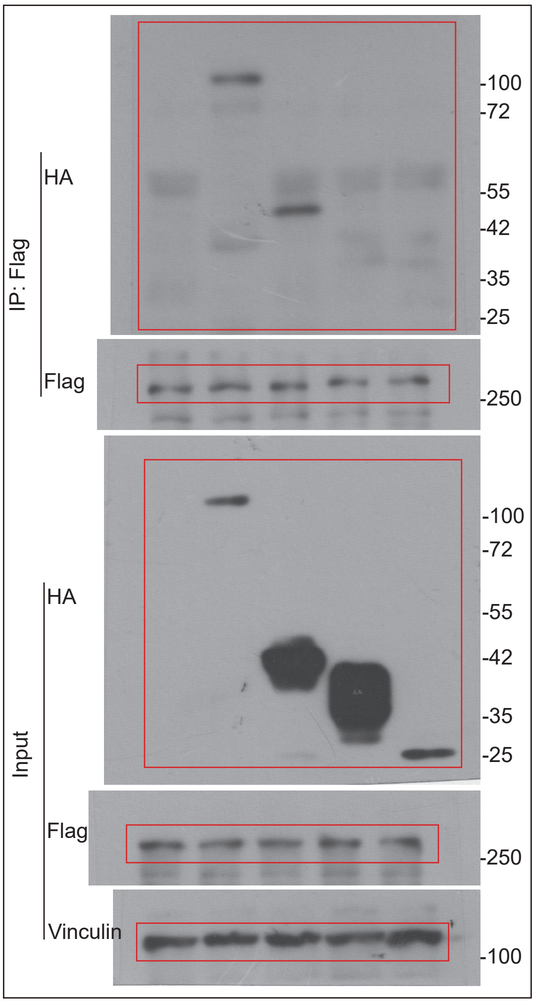

Figure 3H

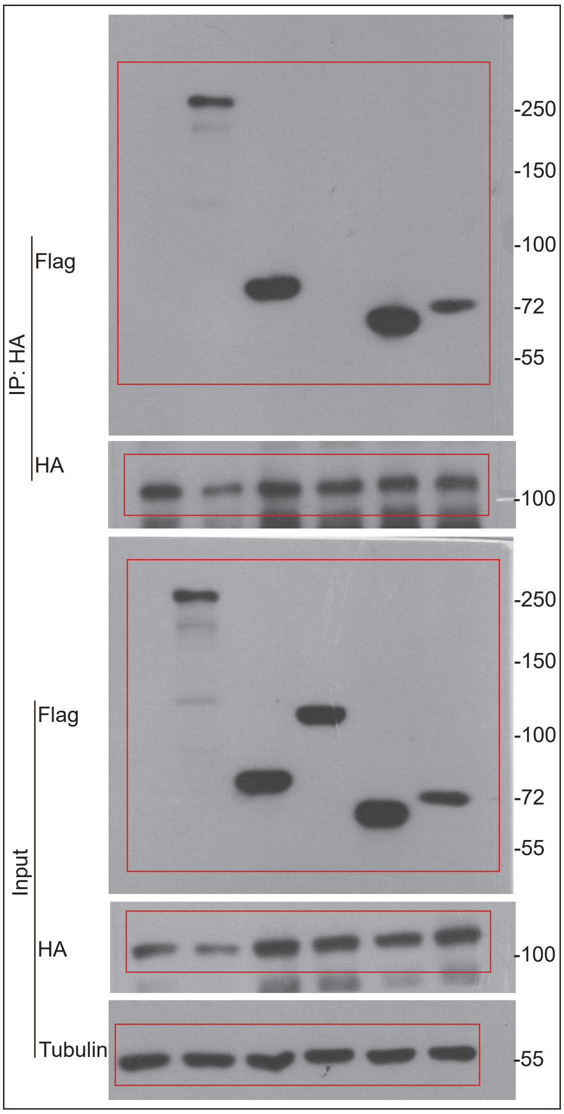

Figure 3I

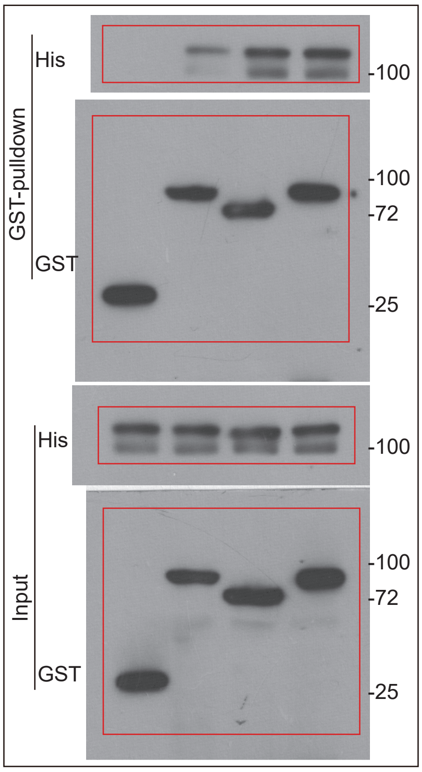

Figure 4A

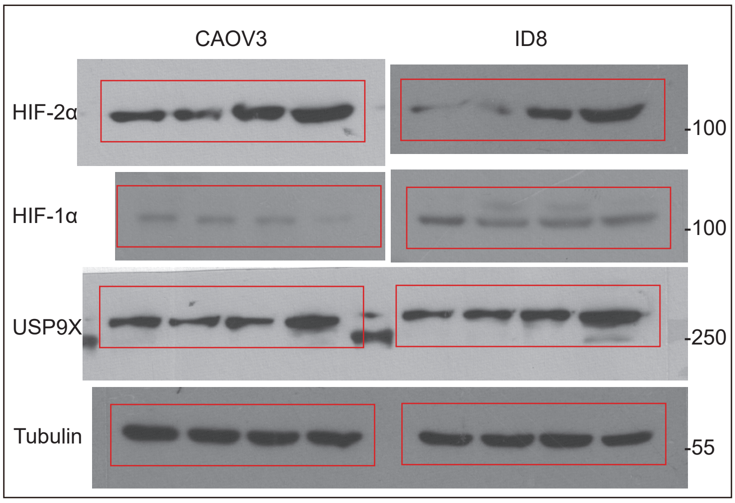

Figure 4B

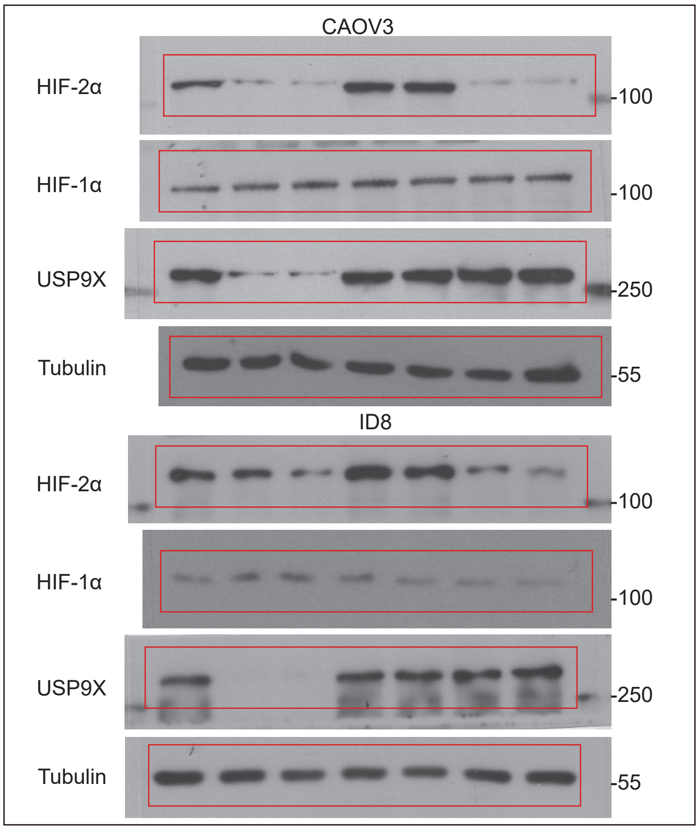

Figure 4C

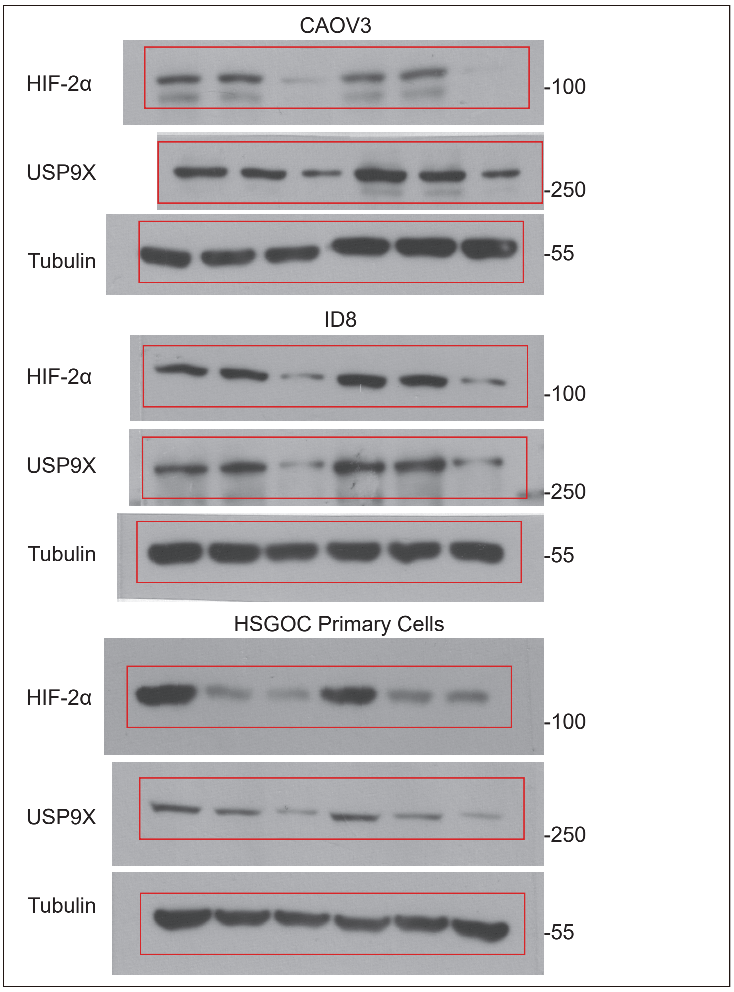

Figure 4D

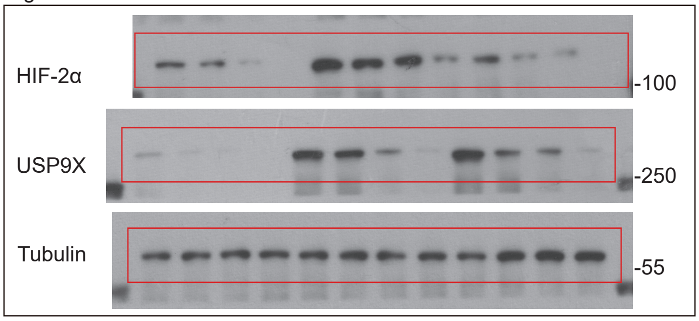

Figure 4E

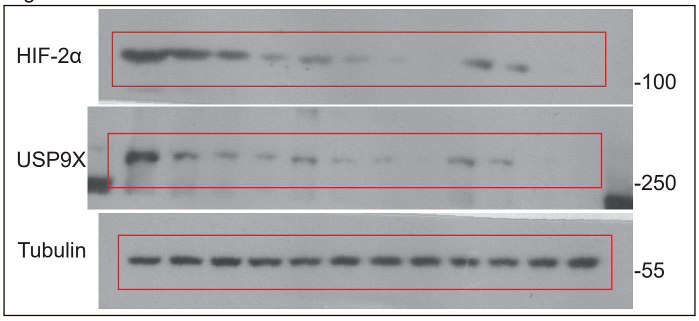

Figure 4F

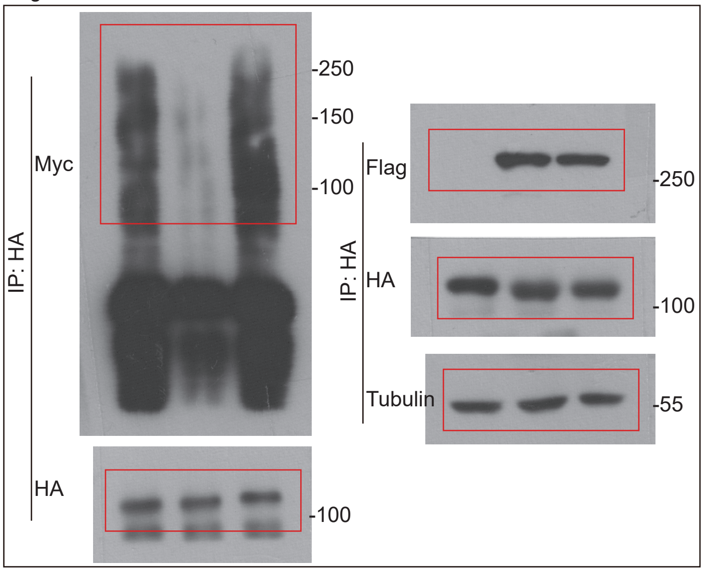

Figure 4G

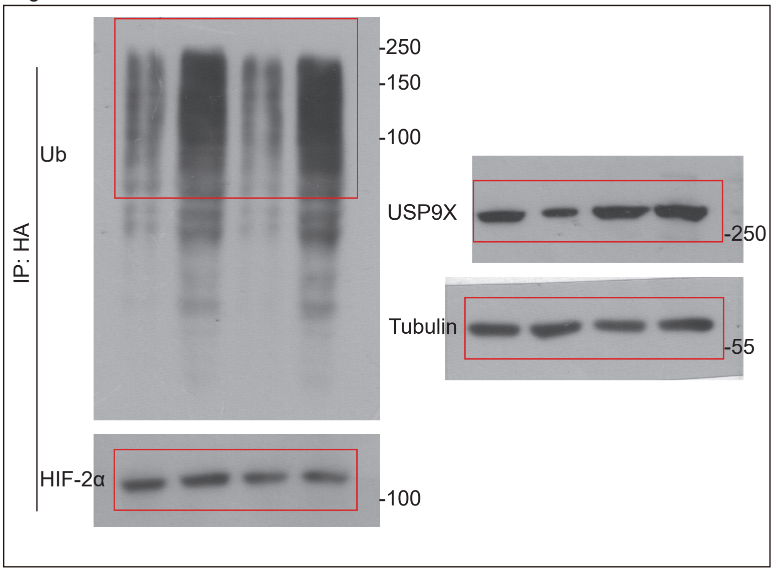

Figure 4H

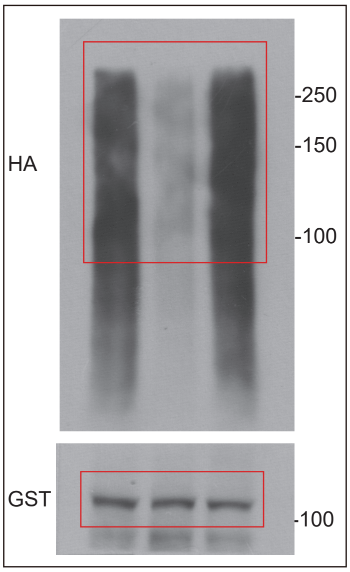

Figure 4I

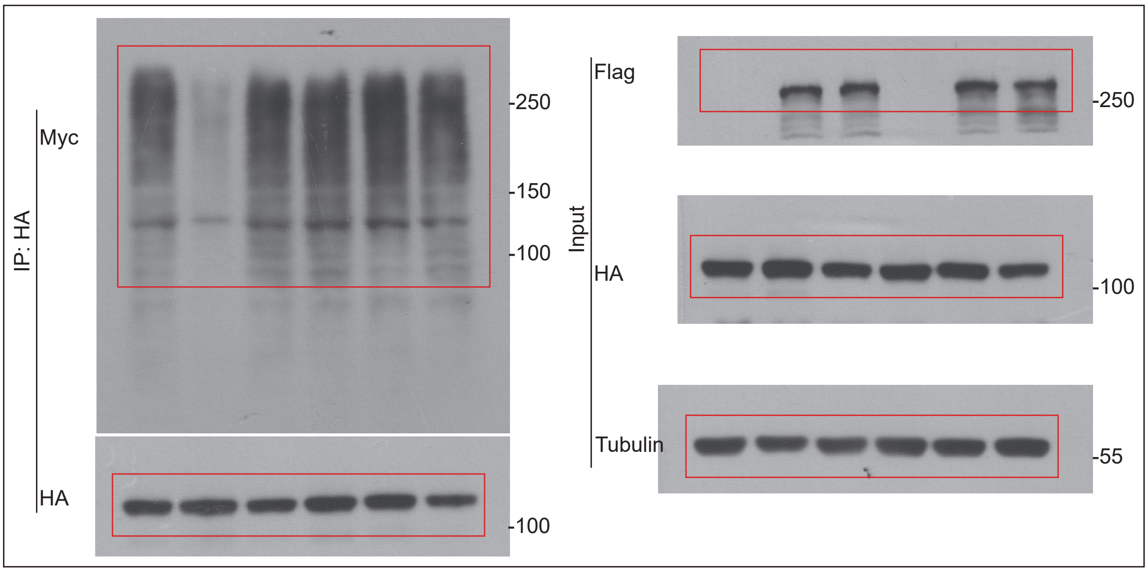

Figure 4J

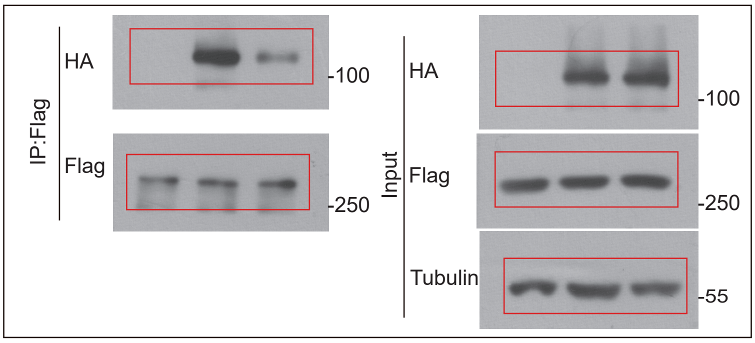

Figure 5A

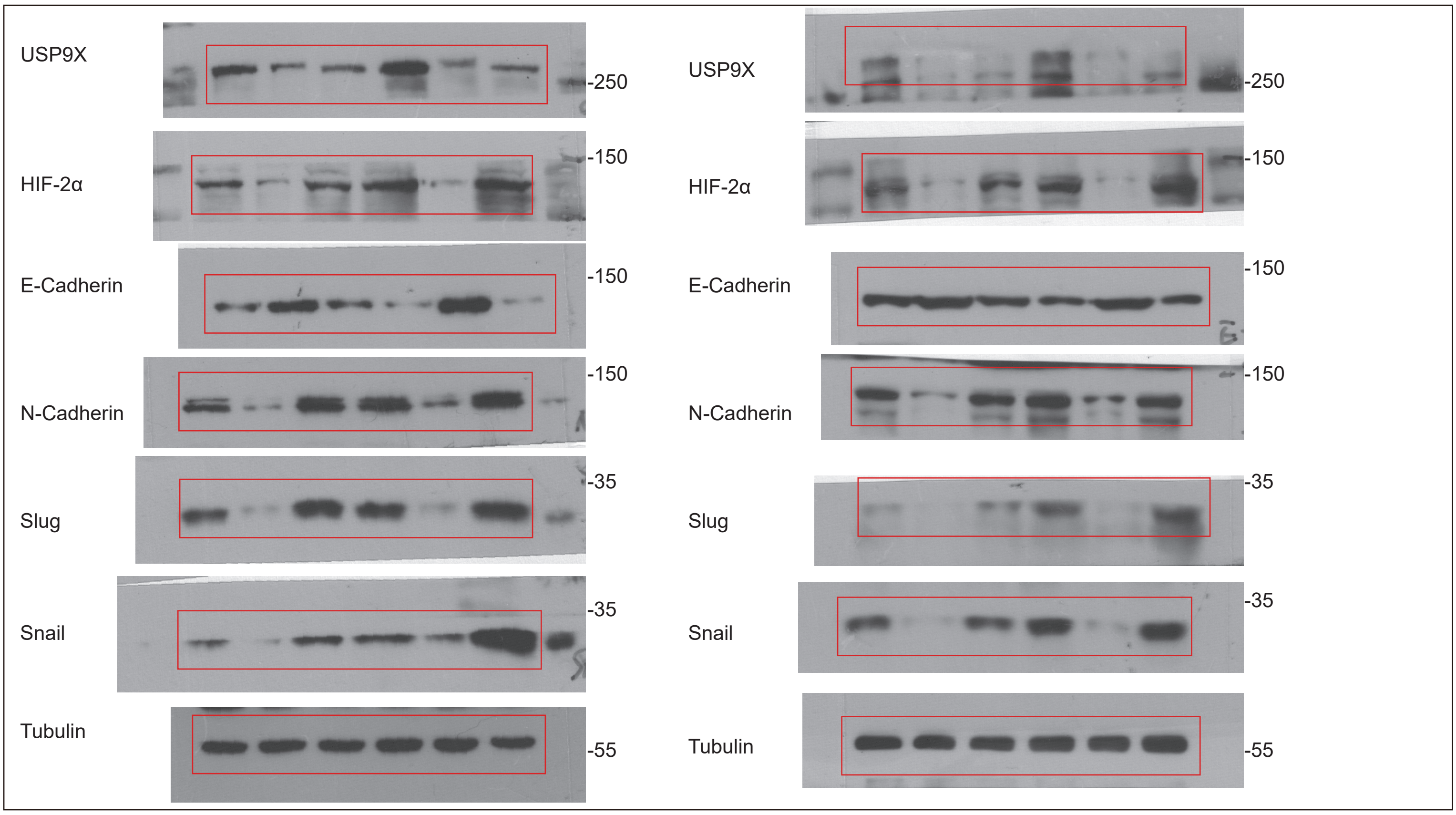
USP9X
-250
HIF-2α
-150
E-Cadherin
-150
N-Cadherin
-150
Slug
-35
Snail
-35
Tubulin
-55
USP9X
-250
HIF-2α
-150
E-Cadherin
-150
N-Cadherin
-150
Slug
-35
Snail
-35
Tubulin
-55

Figure 6A

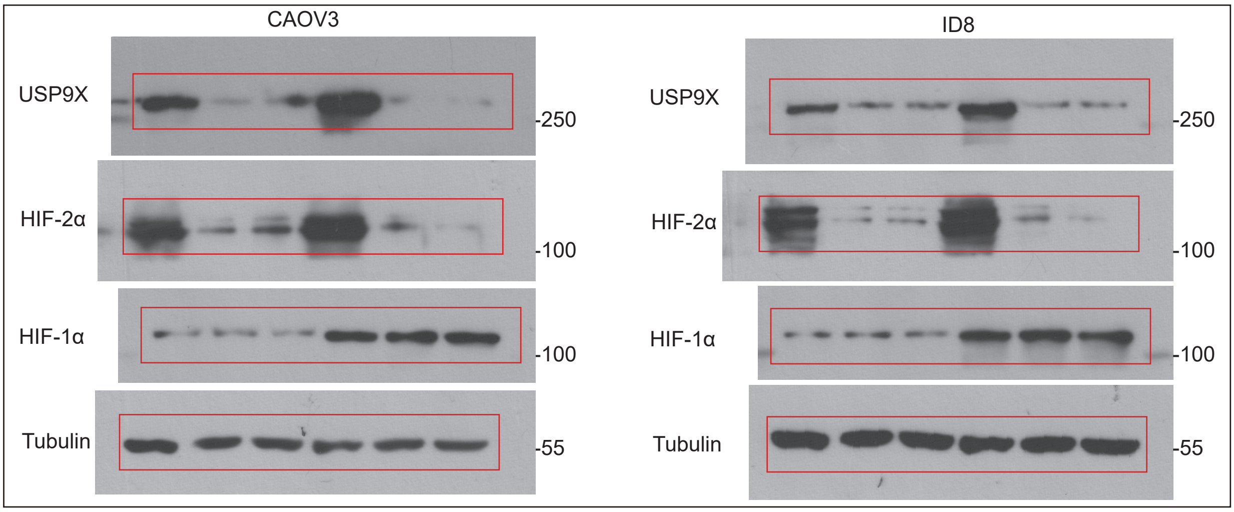

Figure 6C

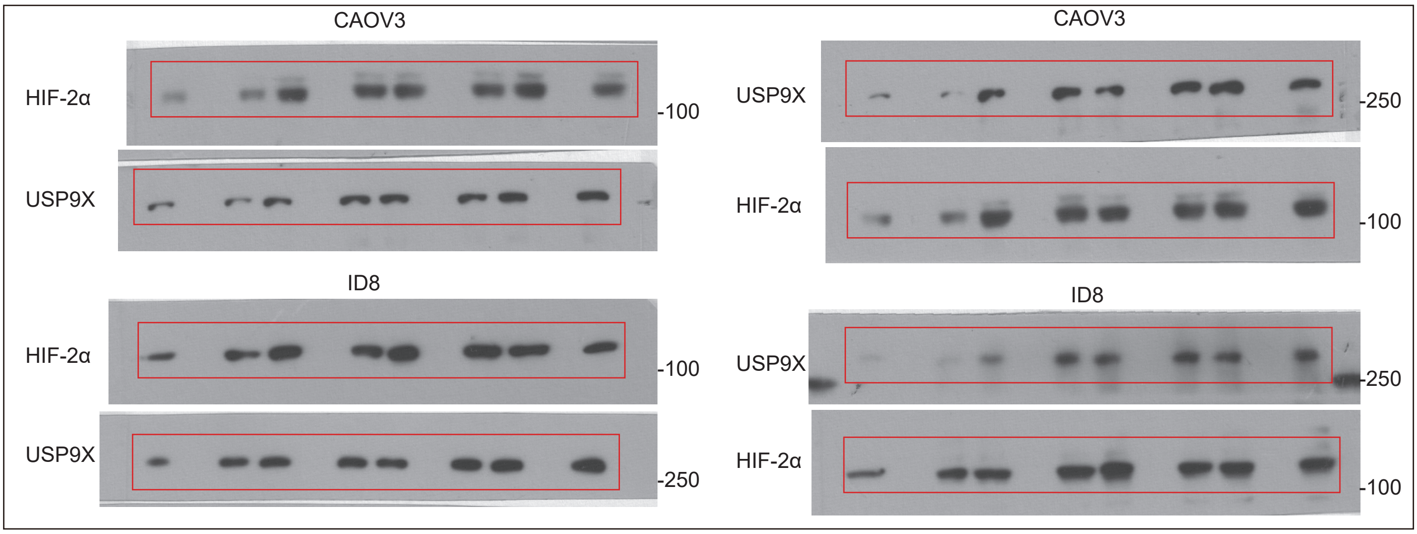

Figure 6D

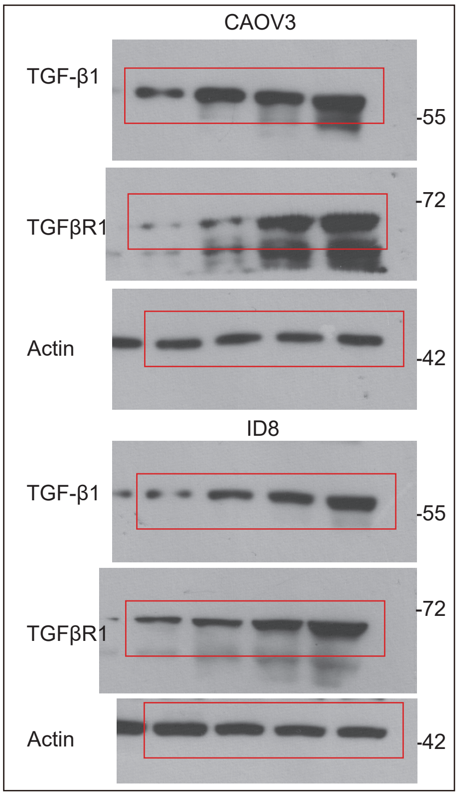

Figure 6E

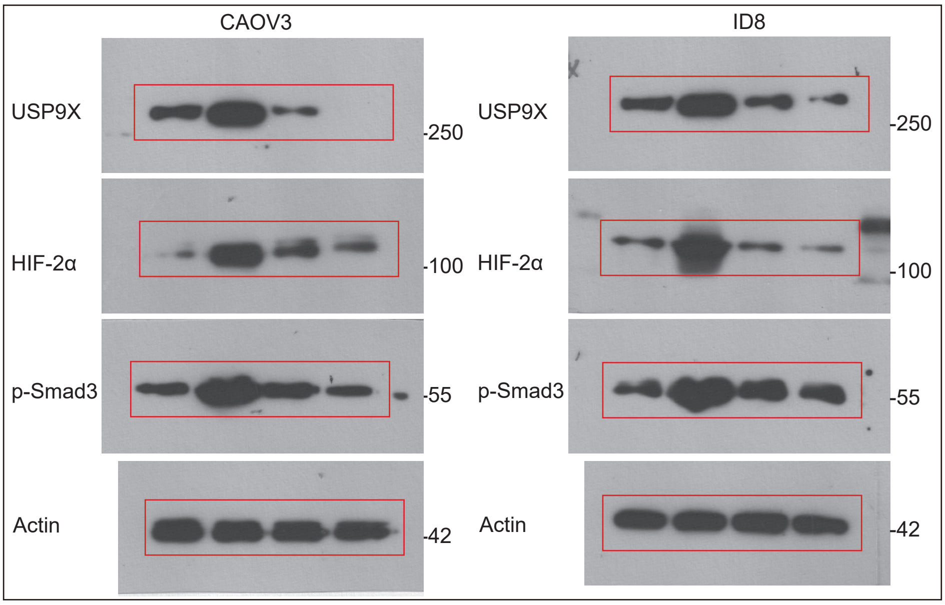

Supplementary Fig. S1C

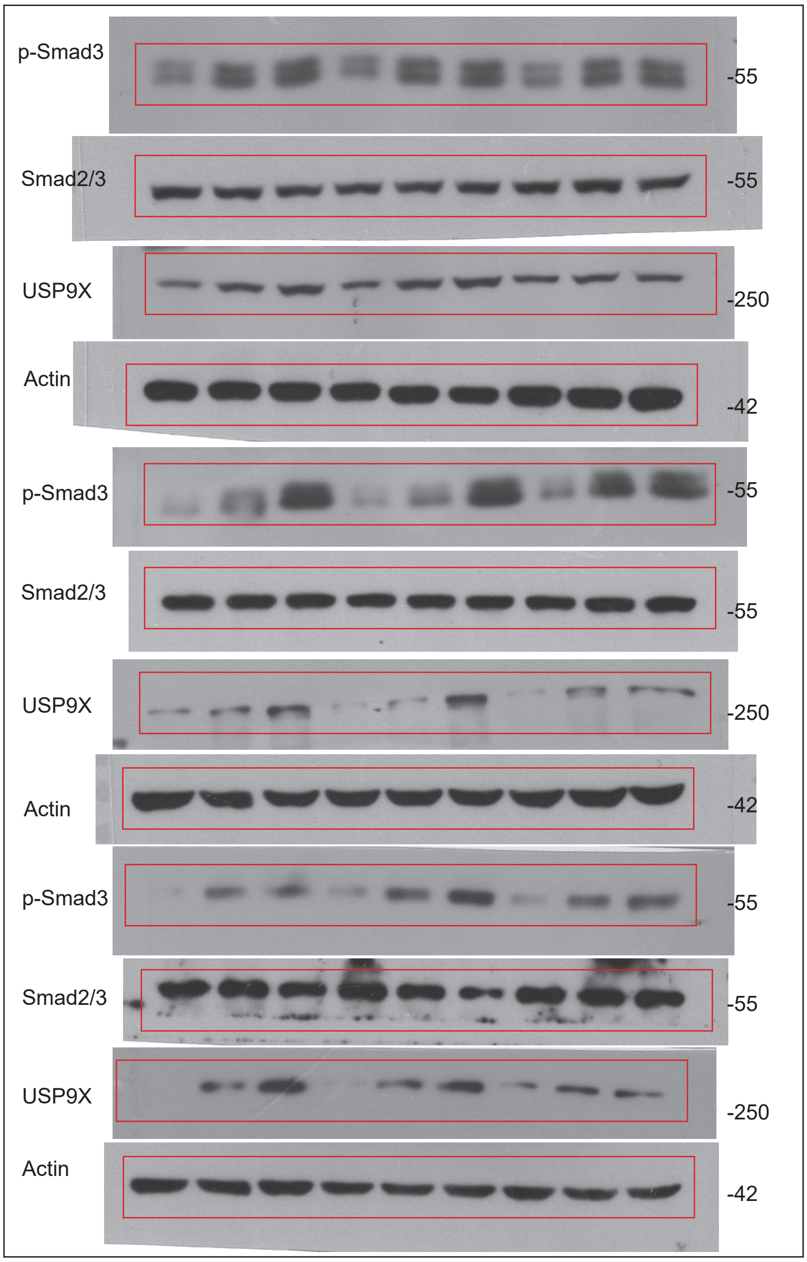

Supplementary Fig. S1J

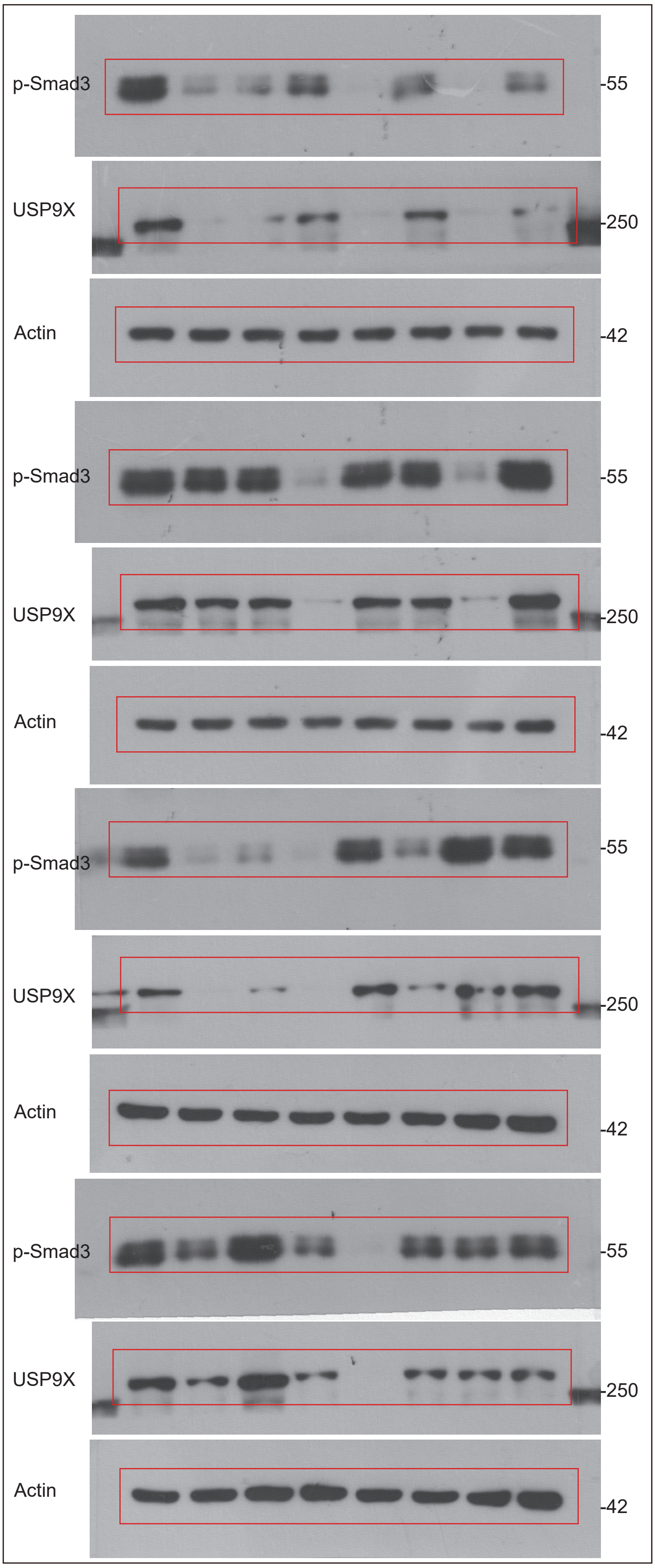

Supplementary Fig. S1N

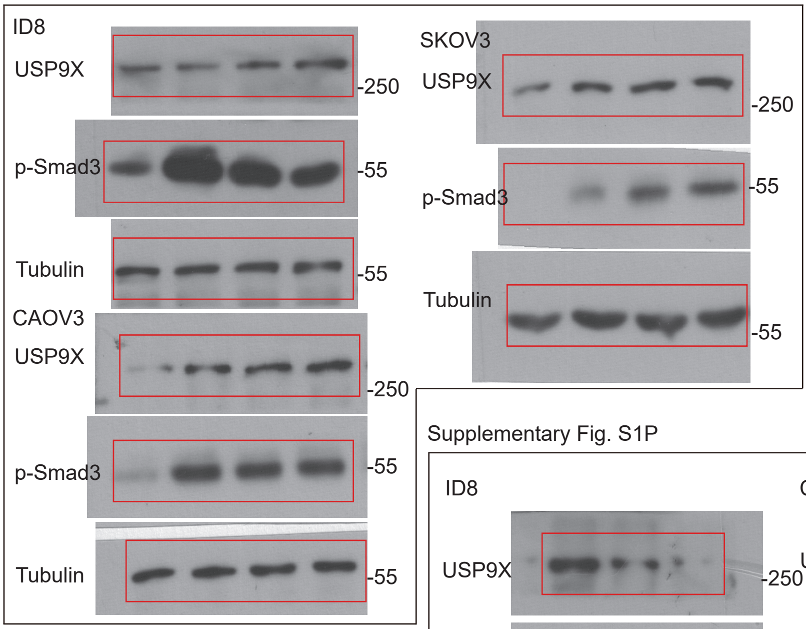

Supplementary Fig. S1P

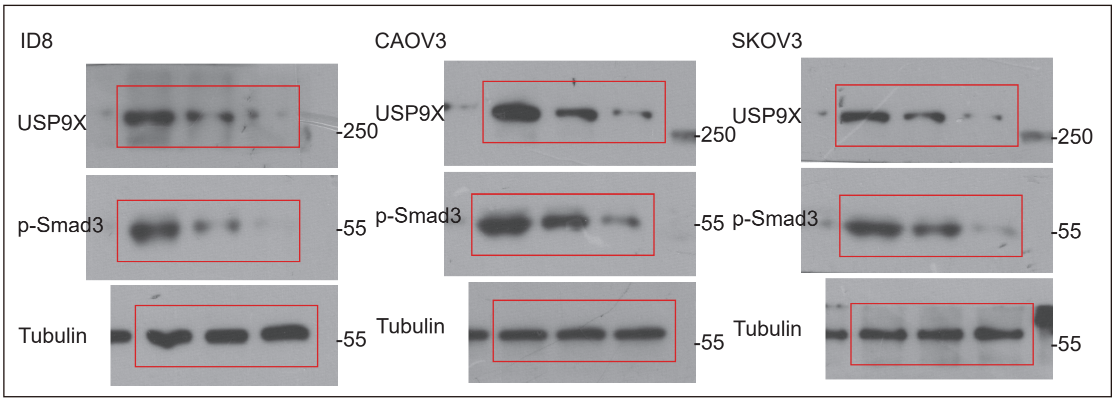

Supplementary Fig. S2E

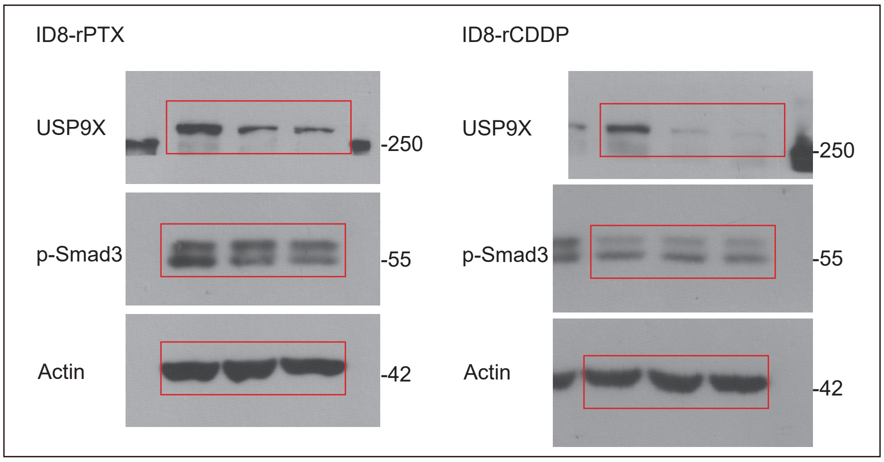

Supplementary Fig. S3B

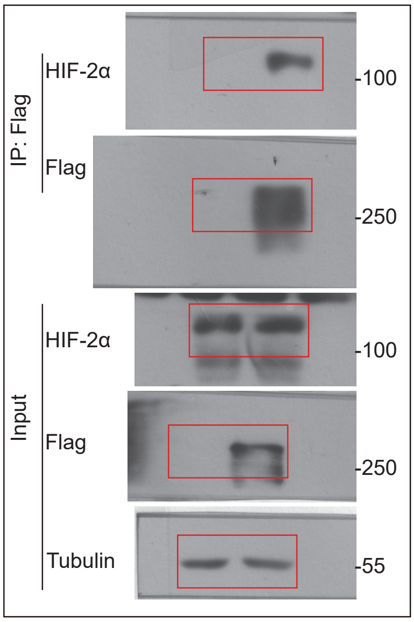

Supplementary Fig. S3C and D

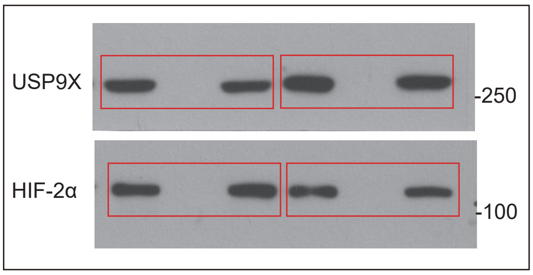

Supplementary Fig. S4A

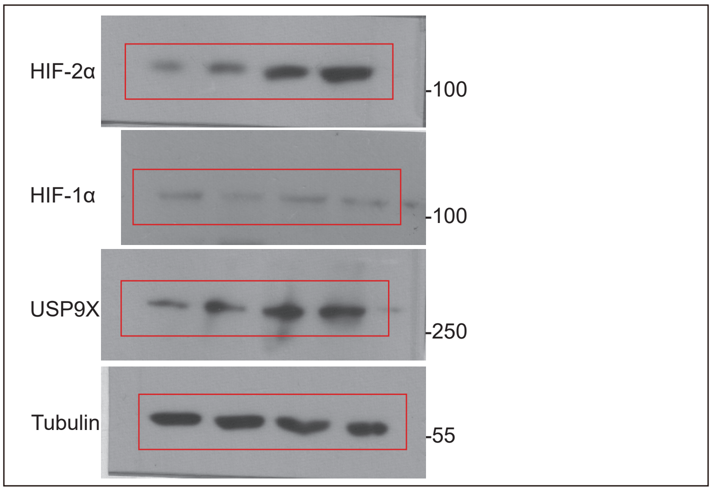

Supplementary Fig. S4B

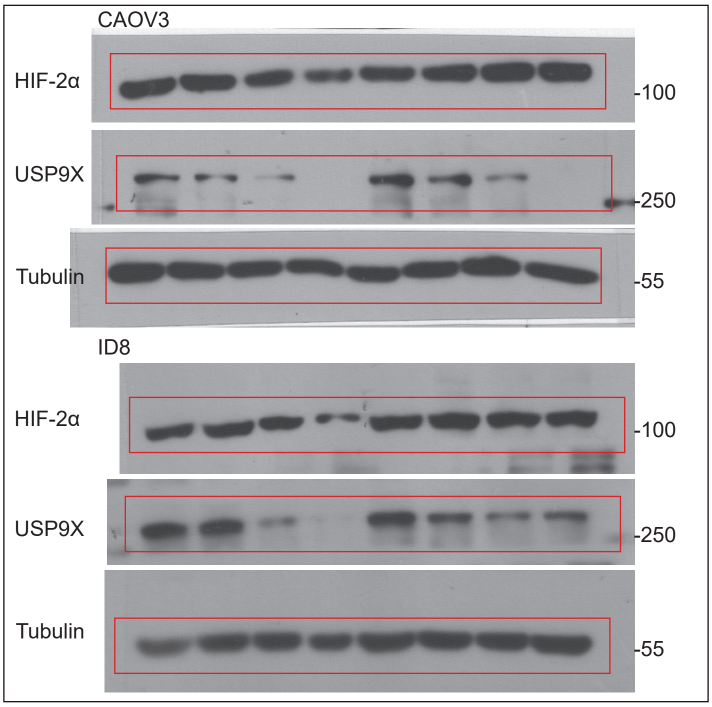

Supplementary Fig. S4C

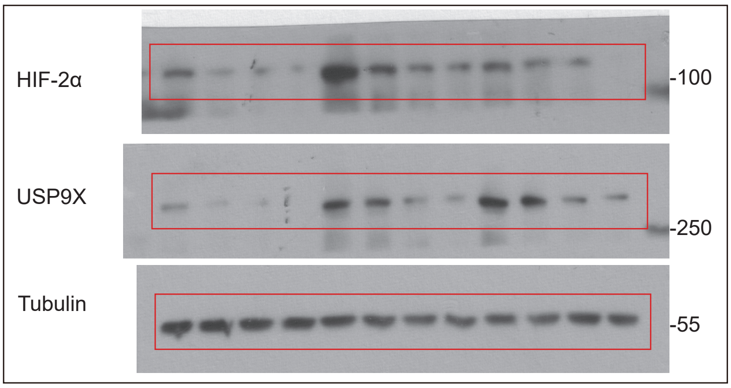

Supplementary Fig. S4D

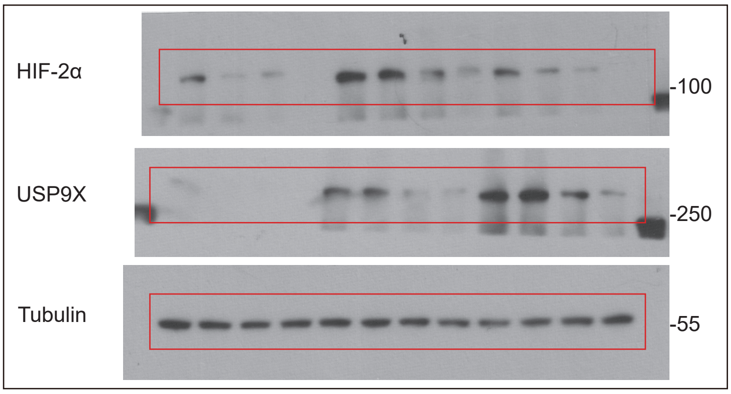

Supplementary Fig. S4E

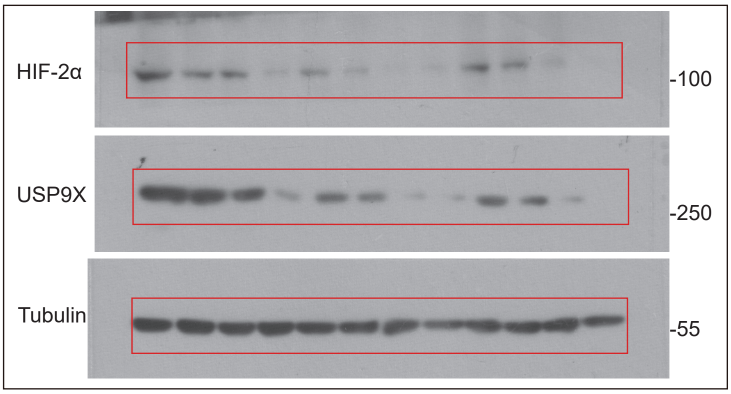

Supplementary Fig. S4F

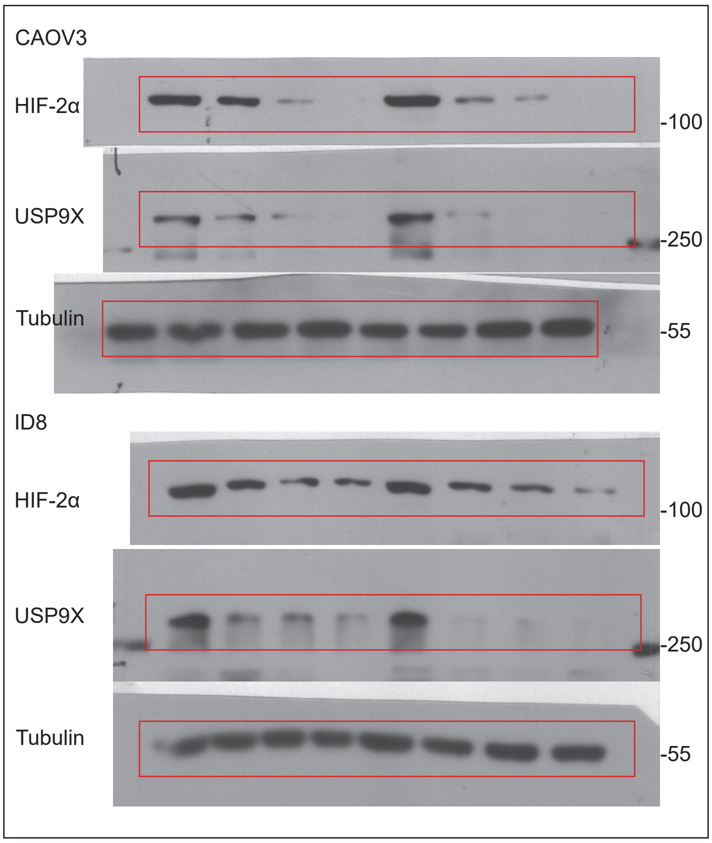

Supplementary Fig. S4H

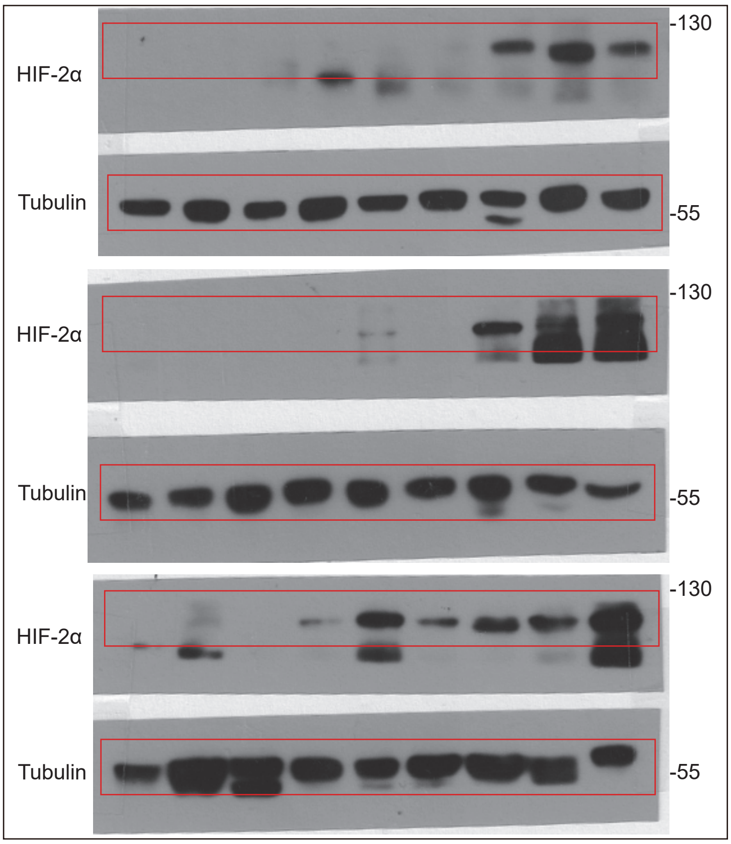

Supplementary Fig. S5F

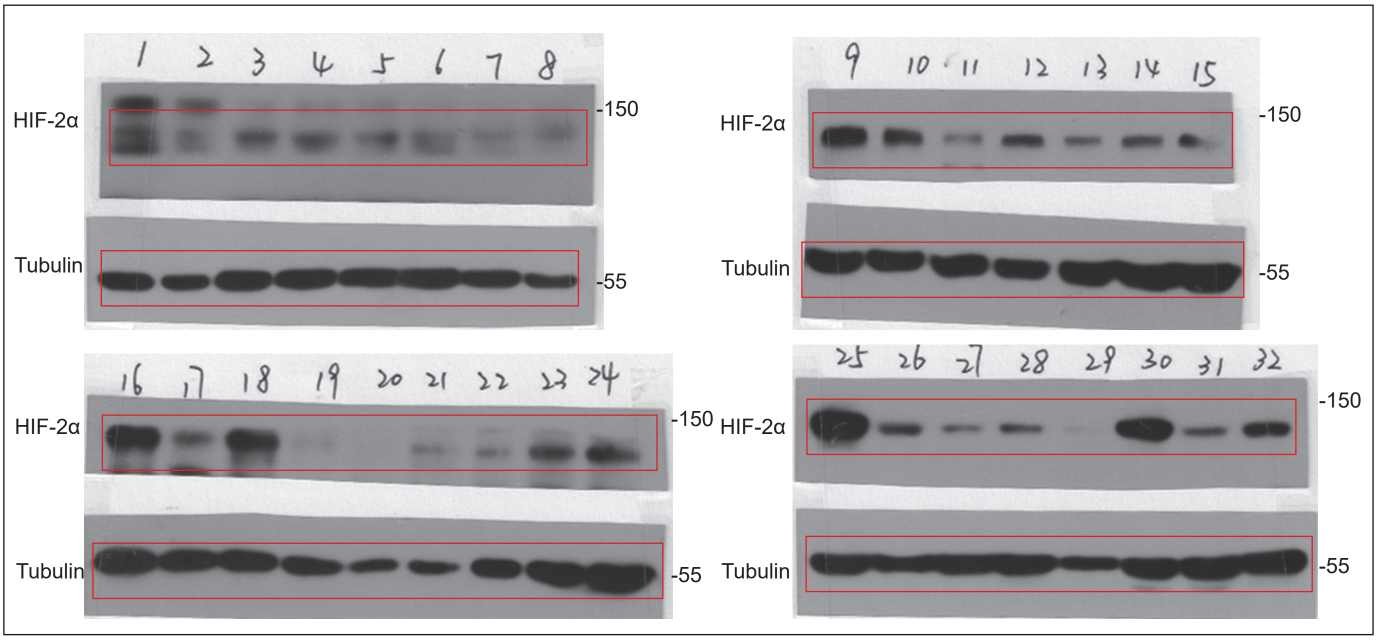

Supplementary Fig. S6A

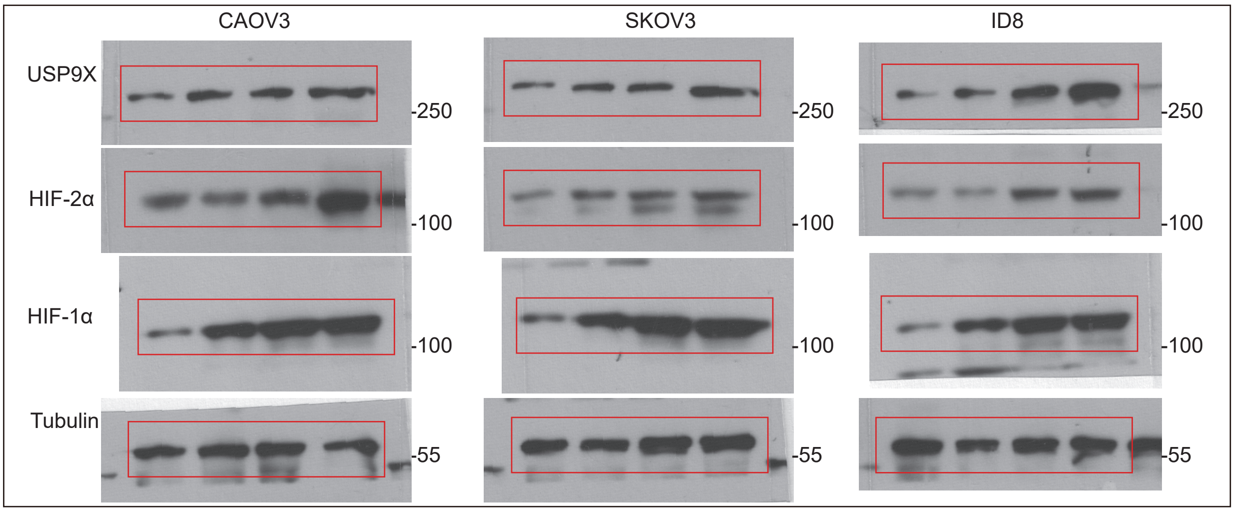

Supplementary Fig. S6C

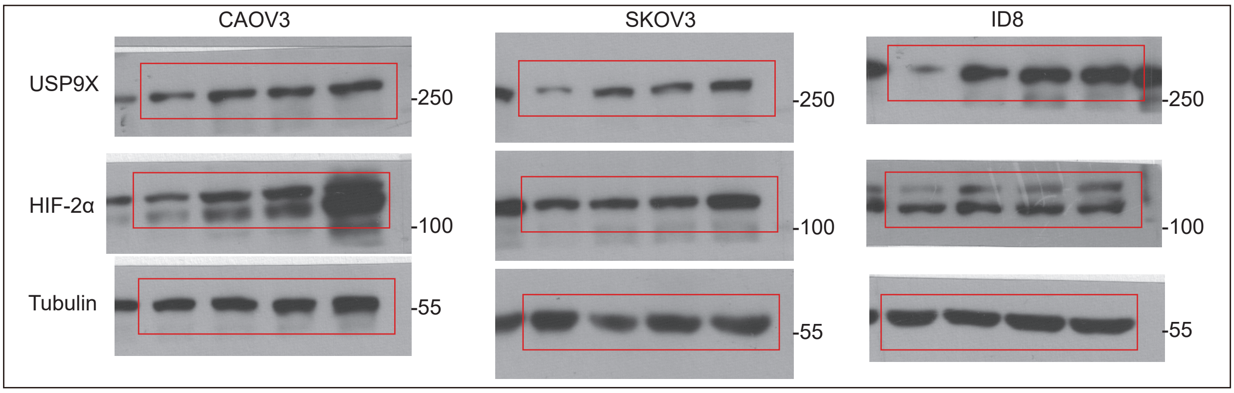

Supplementary Fig. S6G

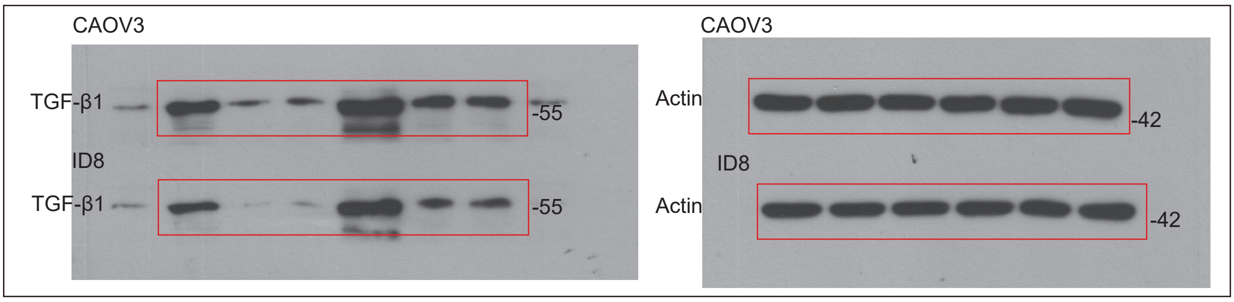

Supplement: Supplementary file 20 — Uncropped Blots [file 41419_2025_7646_MOESM20_ESM.pdf]
